# Data from a survey of the Philippines’ local governments on their risk management strategies to natural disasters

**DOI:** 10.1016/j.dib.2020.106548

**Published:** 2020-11-19

**Authors:** Majah-Leah V. Ravago, Claire Dennis S. Mapa, Jun Carlo Sunglao, Angelie Grace Aycardo

**Affiliations:** a University of the Philippines, Diliman, Quezon City, Philippines; b Philippine Statistics Authority, Quezon City, Philippines

**Keywords:** Disaster risk management, Coping strategies, Local government units, Philippines, Disaster risk management index, Natural hazards

## Abstract

This data is from a survey of Local Government Units Disaster Risk Reduction and Management (DRRM) Office in the Philippines. Conducted in 2016–2017, the survey was intended to assess the disaster risk reduction and mitigation programs and policies employed by the local government on types of disaster due to natural hazards. The survey data covers 47 provinces (including Metro Manila) with 193 municipalities and cities. The sampling design followed a multi-stage probability scheme taking into account the high-risk and low-risk disaster areas. This data article describes the framework and design of the survey and highlights the creation of indices and other outcome variables based on the survey. It also provides information on the field operations including data cleaning and processing that may be useful to those undertaking similar surveys. The dataset is in comma-separated values file (.csv) with accompanying data dictionary (.txt). The questionnaire is also included in the data supplementary appendix. This data article is an adjunct to the research article, “Localized disaster risk management index for the Philippines: Is your municipality ready for the next disaster?” Ravago, et al., 2020, where data interpretation and analysis can be found.

## Specifications Table

**Table:** 

Subject	Economics & Econometrics; Development
Specific subject area	Risk management and coping strategies to disasters caused by natural hazards
Type of data	Comma-separated values file (.csv) Data dictionary (.txt) and (.dct) Questionnaire (.pdf) Table Image Maps
How data were acquired	Face-to-face directed interview Survey questionnaire can also be accessed via Mendeley Data: Ravago, Majah-Leah; Mapa, Claire Dennis; Sunglao, Jun Carlo; Aycardo, Angelie Grace (2020), “Data from a Survey of the Philippines’ Local Governments on their Risk Management Strategies to Natural Disasters ”, Mendeley Data, V1, doi: 10.17632/9y2yhw45zt.1 [1] https://data.mendeley.com/datasets/9y2yhw45zt/draft?a=83106d39–3aee-4133-a4d4–07be3d55e0f3
Data format	Raw data in CSV format Survey data can also be accessed via Mendeley Data. Ravago, Majah-Leah; Mapa, Claire Dennis; Sunglao, Jun Carlo; Aycardo, Angelie Grace (2020), “Data from a Survey of the Philippines’ Local Governments on their Risk Management Strategies to Natural Disasters ”, Mendeley Data, V1, doi: 10.17632/9y2yhw45zt.1 [1] https://data.mendeley.com/datasets/9y2yhw45zt/draft?a=83106d39–3aee-4133-a4d4–07be3d55e0f3
Parameters for data collection	The data derives from a survey of Local Government Units (LGUs) Disaster Risk reduction and Management (DRRM) Office in the Philippines. The respondents were the DRRM officer(s) at the time when the survey was conducted from November 2016 to April 2017 and from September to October 2017.
Description of data collection	The default method of the survey was a face-to-face directed interview with the DRRM Officer(s) of the LGUs. The data contains the incidence of disaster, related damages and state of recovery; ex-ante public controls, ex-post loss reduction, and coping mechanisms the LGUs undertake to mitigate the adverse effects of disasters due to the natural hazard. It also includes data on their perception of risk. It has a specific information on action related to the agricultural sector. The data also contains general information on the LGU, DRRM officers, and DRRM office. It also has information on training of their personnel and assets related to disaster response. The conduct of this survey fulfilled the technical requirements necessary to demonstrate the use of ethical procedures in researching human participants. Implicit informed consent has been obtained from the respondents because they have agreed to be interviewed. The respondents are public officials and public servants. The information requested from them are related to their work and activities of the local public office. No personal identifiable information of the respondents is included in the data. Fig. 1 is a map of the risk classification and geographical distribution of sample LGUs. This is a secondary data from Manila Observatory [4]. Fig. 2 illustrates the components of the 19 Risk Management (DRM) Sub-indices developed in the study. Table 1 provides the number and geographical distribution of sampled municipalities included in the study. Table 2 provides a description of the coverage of the survey data. Table 3 provides a profile of the sample LGUs.
	Table 4 provides a profile of the local DRRM offices included in the study. Table 5 provides a summary of the number of times disaster has been experienced since 2009/1980 Table 6 provides a summary on the number of municipalities that experienced several disasters. Table 7 provides a list and description of the disaster risk management sub-indices developed in the study. Supplementary Appendix A provides the raw data in comma-separated values file (.csv) with accompanying data dictionary in (.txt) and (.dct) format [1]. Supplementary Appendix B [1] is the questionnaire. Supplementary Appendix C [1] provides the equations of various sub-indices developed in this study.
Data source location	The data covers 47 provinces (including Metro Manila) with 193 municipalities and cities of the Philippines. Attached in this data article. See also Ravago, Majah-Leah; Mapa, Claire Dennis; Sunglao, Jun Carlo; Aycardo, Angelie Grace (2020), “Data from a Survey of the Philippines’ Local Governments on their Risk Management Strategies to Natural Disasters ”, Mendeley Data, V1, doi: 10.17632/9y2yhw45zt.1 [1]
Data accessibility	Ravago, Majah-Leah; Mapa, Claire Dennis; Sunglao, Jun Carlo; Aycardo, Angelie Grace (2020), “Data from a Survey of the Philippines’ Local Governments on their Risk Management Strategies to Natural Disasters ”, Mendeley Data, V1, doi: 10.17632/9y2yhw45zt.1 https://data.mendeley.com/datasets/9y2yhw45zt/draft?a=83106d39–3aee-4133-a4d4–07be3d55e0f3 Instructions for accessing these data: Standard access via Mendeley Anonymized **raw data set** in comma-separated values file (.csv) with accompanying data dictionary (.txt or .dct): Supplementary Appendix A1 DIB Ravago et al. 2020.csv Supplementary Appendix A2 DIB Ravago et al. 2020.txt Supplementary Appendix A3 DIB Ravago et al. 2020.dct **Questionnaire** Supplementary Appendix B DIB Ravago et al. 2020 questionnaire.pdf **Sub-indices equations** Supplementary Appendix C DIB Ravago et al. 2020 sub-index equations.pdf
Related research article	M.V. Ravago, D. Mapa, A. Aycardo, and M. Abrigo, 2020, “Localized Disaster Risk Management Index for the Philippines: Is Your Municipality Ready for the Next Disaster?” International Journal of Disaster Risk Reduction, https://doi.org/10.1016/j.ijdrr.2020.101913 [2]

## Value of the Data

• The data and methodology are examples of instruments that allow researchers to evaluate the risk management and coping strategies of public offices at the local level.
• The survey instrument and the methodology offer potential for the Philippine government to scale the size of data collection to include all LGUs in the survey.
• They can be replicated in other countries for comparison of risk management and coping strategies employed by local public offices to mitigate the adverse effects of disasters.
• The data also include disasters due to geologic hazard that have low probability and less frequently occurring. Researchers can use the data to evaluate the difference on how public office responds to disaster caused by hydrometeorology or geologic hazards.
• The data may be used by researchers to develop longitudinal studies that would allow estimation of dynamic effect of disasters at the local level.
• The data may be combined with other datasets on disasters at the LGU level for further studies to include financing, insurance, among others.

## 1 Data Description

This data is from a survey of Local Government Units (LGUs) Disaster Risk Reduction and Management (DRRM) Office in the Philippines. Conducted in 2016–2017, the survey was intended to assess the disaster risk reduction and mitigation programs and policies employed by the local government on types of disaster due to natural hazards. The survey data covers 47 provinces (including Metro Manila) with 193 municipalities and cities. The sampling design followed a multi-stage probability scheme taking into account the high-risk and low-risk disaster areas. This data article describes the framework and design of the survey that allows assessment of the various disaster risk reduction and mitigation activities employed by the LGUs. It highlights the creation of indices and other outcome variables based on the survey. It also provides information on the field operations, data cleaning and processing that are useful for those undertaking similar surveys. Supplementary Appendix A provides the dataset in comma-separated values file (.csv) with accompanying data dictionary in (.txt) and (.dct) formats [1]. The full questionnaire is provided in Supplementary Appendix B [1]. This data article is related to the research article, “Localized Disaster Risk Management Index for the Philippines: Is Your Municipality Ready for the Next Disaster?,” Ravago, et al. [2] and “Coping with disasters due to natural hazards: Evidence from the Philippines,” Ravago, et al. [3], where data interpretation and analysis can be found.

## 2 Experimental Design, Materials and Methods

The actual impact of natural disasters, in terms of economic and human damages, is determined by the interaction of nature and society. Among other things, this includes: (a) susceptibility of infrastructure, food, housing, and economic condition; (b) risk reduction through the use of appropriate early warning system, healthcare, social, and material coverage; (c) coping strategies of both households and governments; and (d) adaptive capacities related to future natural hazards and the impacts of climate change. Moreover, it is recognized that the government has a vital role in reducing susceptibility and mitigating the impact of disasters through its various programs and policies.

Our data allows researchers to explore how LGUs in the Philippines respond to disasters caused by natural hazards. It provides information on the mix of ex-ante and ex-post risk management strategies that the LGUs implement to improve welfare of its constituents. The data is particularly focused on the initiatives of LGUs due to the following reasons: (a) the various ex-ante and ex-post programs at the local level can be easily investigated, particularly the variations in the type and level of programs that aim to mitigate the impact of the disasters and achieve full recovery; and (b) disaster impact is inherently local and a survey based on the municipal or city level would allow a higher resolution in terms of data generated where DRRM policies are fully implemented.

The data is useful for researchers who wish to investigate the economic dynamics of the country's disaster management system at the LGU level.

### 2.1 Sampling design

**Fig. 1:**
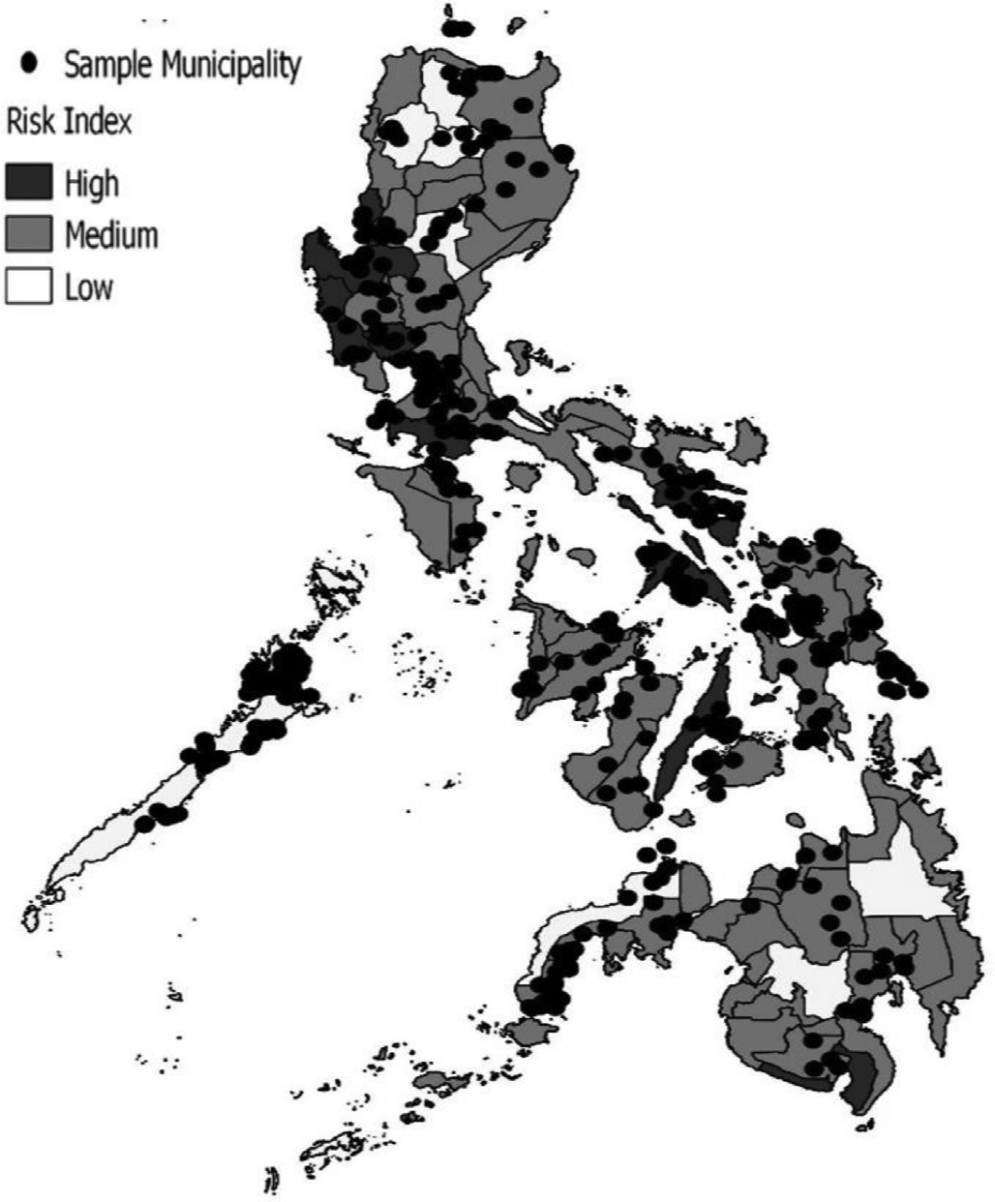
Risk classification and geographical distribution of sample LGUs.

The sampling design follows a multi-stage probability scheme. The domain of the analysis comprises all municipalities in the Philippines. The sampling frame for the high-risk and low-risk provinces was generated based on risk mapping from the Manila Observatory [4]. Meanwhile, high-risk and low-risk municipalities were determined using risk area incidence as reported by the Project NOAH [5] in 2015 indicating the relative level of risk among municipalities within the province. Fig. 1 shows the risk classification by province and the geographical distribution of 193 sample municipalities and cities.

Source of basic data on mapping: Manila Observatory [4].

The sampling weights are computed as follows. The selection probability $p_{\text{ij}}$ for the municipality in the $j^{\text{th}}$ province of the $i^{\text{th}}$ region is given by the formula:

$$p_{\text{ij}}=\frac{N_{i}^{P}}{N_{i}^{R}}\times \frac{n_{\text{ij}}^{P}}{N_{i}^{P}}\times \frac{n_{\text{ij}}^{M}}{N_{\text{ij}}^{P}}=\frac{n_{\text{ij}}^{P}}{N_{i}^{R}}\frac{n_{\text{ij}}^{M}}{N_{\text{ij}}^{P}}$$

where: $N_{\text{ij}}^{P}$and $N_{i}^{R}$ are the numbers of municipalities in the province and region in the country, respectively, $n_{\text{ij}\;}^{P}$is the number of municipalities in the selected province, and $n_{\text{ij}}^{M}$ is the number of municipalities in the sample in the *i^th^* province of the *j^th^* region. The basic sampling weight is the inverse of the selection probability and is given by:

$$w_{\text{ij}}=\frac{1}{p_{\text{ij}}}=\frac{N_{i}^{R}}{n_{i}^{P}}\frac{N_{\text{ij}}^{P}}{N_{\text{ij}}^{M}}$$

Adjusted sampling weight ($w_{\text{ij}}^{P}$) is given by:

$$w_{\text{ij}}^{P}=w_{\text{ij}}\frac{N}{\sum_{j}w_{\text{ij}}n_{\text{ij}}^{M}}$$

Finally, the final adjusted sampling weight ($w_{\text{ij}}^{F}$) is given by:

$$w_{\text{ij}}^{F}=w_{\text{ij}}^{P}\frac{N}{\sum_{i}\sum_{j}w_{\text{ij}}^{P}n_{\text{ij}}^{M}}$$

where N is 1488 – the total number of municipalities in the Philippines as reported by PSA [6].

Given the 1488 municipalities and 81 provinces within the Philippines, a sample size of 177 municipalities was initially selected for the nationally representative sample. This was distributed proportionately into five broad regions – 10 provinces and 40 cities for each of the four regions, along with the municipalities of Metro Manila. This was done to ensure a representative sample.

Proportionate sampling was also employed to all provinces for a more accurate coverage. A mix of relative high-risk and low-risk municipalities was chosen in order to adequately cover the range of disaster impacts that different municipalities may face.

Due to the imposition of Martial Law in Mindanao on May 23, 2017, three provinces (North Cotabato, Lanao del Sur, and Maguindanao) became ineligible for interview due to the increased security risk. For various reasons, two of the supposed samples in Metro Manila and Cavite have not been interviewed. These circumstances prompted the team to look for other provinces from Luzon and Visayas as replacements. Replacements were likewise based on regional and provincial risk, poverty incidence, household income, and population. Thirty-two (32) municipalities were identified as replacements to compensate for the required sample size in the study. These municipalities came from the four provinces of the Cordillera Administrative Region (CAR) and four provinces of the Eastern Visayas. To sum up, the study covers 47 provinces (including Metro Manila) with 193 municipalities and cities. Table 1 shows the disaggregation of sample size.

**Table 1:** Number and geographical distribution of sampled municipalities.

	Provincial-Municipal Risk Index						
Areas	Low-Low	Low-High	Med-Low	Med-High	High-Low	High-High	Total
National Capital Region	–	–	8	4	–	–	12
North and Central Luzon	9	7	12	11	7	10	56
South Luzon and Bicol	3	1	11	11	10	6	42
Visayas	1	3	19	25	2	2	52
Mindanao	2	2	16	11	0	0	31
**Total**	**15**	**13**	**66**	**62**	**19**	**18**	**193**

### 2.2 Development and design of survey instrument

#### 2.2.1 Concepts and definitions

Natural hazards are those elements of the physical environment that can harm the population. Natural hazards are categorized in this study as: (1) strong winds and rain; (2) floods due to continuous rain, storms, etc.; (3) landslide/mudslide; drought; (4) drought due to extreme heat; (5) big waves, including tsunami and storm surge; (6) biological hazards e.g. leptospirosis; (7) earthquakes; and (8) volcanic eruptions and/or lava/lahar flow. While natural hazards can be reasonably expected to occur, their full features cannot be predicted accurately.

A natural hazard is elevated to disaster status when it leads to a decrease in the welfare (well-being) of households or individuals. A reduction in welfare occurs when there is death, injury, and damage in infrastructure.

#### 2.2.2 The survey instrument

Guided by the general framework for disaster risk management developed in Ravago et al. [7] and Ravago et al. [8], we developed the instrument for the Survey of Local Government Units DRRM Office. We refer to the questionnaire of the Philippine Center for Economic Development Social Protection Survey of households (Ravago et al. [9]) in designingour survey instrument and adapted the questions for the LGUs.

We focus on 8 shocks caused by natural hazards. These 8 shocks are disasters due to (1) strong winds and rains, (2) flood due to continuous rains, storms, (3) landslide/mudslide, (4) big waves including tsunami and storm surge, (5) drought, (6) biological hazards (i.e. leptospirosis), (7) earthquake, and (8) volcanic eruption. Shocks 1 to 6 are restricted to events on or after January 2009, and Shocks 7 and 8 cover events on or after January 1980. The latter captures a wider range of events due to the nature of the shocks, i.e. low-frequency and low-probability of occurring. In contrast, the six shocks are characterized as having a high-frequency and high-probability of occurring. The reference period also captures the changes brought by the formulation and implementation of the enacted law on DRRM [10].

The respondents were the local government units DRRM officers. Since most of the questions are retrospective, the local DRRM officers also recall the exact months and years when the disasters occurred. For the coping mechanisms employed by the LGUs, we examined the DRRM framework adopted and implemented in the country. We initially drew questions on these programs from the Philippine DRRM Act of 2010 [10], which focuses on four areas: disaster prevention and mitigation; disaster preparedness; disaster response; and disaster rehabilitation and recovery.

Important considerations for the questions are consistency, clarity, and awareness. Consistency and clarity are especially important with the target respondents, and the language of the survey catered to their knowledge base and training. Finally, responses regarding type, scope and impact of programs should indicate risk perception of the DRRM officers and thus allow for a comparison to documented risk attitudes.

The questionnaire was also reviewed by the technical staff of the National Economic and Development Authority (NEDA). The functions of the Presidential Assistant for Rehabilitation and Recovery (PARR) resides with NEDA [11]. Changes were made to include proxies that may capture performance DRRM offices.

#### 2.2.3 Structure of the survey instrument

The survey instrument is a questionnaire with seven blocks of questions. It asks for the profile and characteristics of the Local DRRM offices and their respective LGUs, about the general information on the municipality and the DRRM officer. A block of questions asks for trainings conducted and attended by the staff and the assets procured by the DRRM office. Questions for the incidence of shocks or disasters, related damages and state of recovery, ex-ante public controls, on ex-post loss reduction, and on coping mechanisms cover the policy tools available to the municipality in mitigating the adverse effects due to the natural hazard or shock. There are questions on risk perception, which aims to capture the risk profile of the disaster officer with regard to his municipality. The full questionnaire is provided in Supplementary Appendix B [1].

#### 2.2.4 Key features of the questionnaire

In terms of format, the questions were laid out in a matrix, with the shock types in the leftmost column and the specific questions listed across the column headers. This allows for ease of questioning and recording of responses, especially for cases where multiple disaster types were experienced. The questions are also generally formulated to be close-ended whenever possible. In particular, questions involving timelines such as length of implementation or timing of a certain response were coded into ranges and scales. Information on the appropriate scales were derived from various administrative issuances along with key interviews. Overall, careful attention was placed into formulating the questions to ensure ease of encoding and quality control.

The interviews were conducted by two enumerators face-to-face using this questionnaire and aided with show cards. The ratings and scales were also displayed separately using show cards to aid the respondent. See Supplementary Appendix B [1] for the questionnaire.

### 2.3 Pre-testing and conduct of the survey

The survey instrument was pre-tested by interviewing two Barangay Captains in Quezon City. Pretesting helped evaluate how prospective respondents would answer the survey questions and if the sequence of the blocks can be easily followed. This was followed by the pilot interview in Marikina City. Marikina was chosen as the site of the pilot survey because of proximity and their extensive experience with natural disasters. Marikina is also recognized to have one of the best disaster risk reduction and management offices in Metro Manila. The pre-testing and pilot interview highlighted a number of important issues, which were utilized in finalizing the survey instrument as well as formulating the fieldwork manual for enumerators, encoders, and project staff.

From the consultations and the pilot interviews, the fieldwork procedure was formulated:

• Interviews were scheduled by the enumerators themselves, with full support and assistance from the project staff. This minimized miscommunication and allowed for a smoother scheduling of interviews.
• Interviews were coordinated in batches of 2–4 nearby provinces in order to maximize efficiency. Once coordinated, travel and accommodation were arranged.
• Normal interviews should take anywhere from over an hour to close to two hours, with the median at one and a half hours long. Avoiding long interviews is important.
• Blocking questions together and asking the appropriate order is crucial in question recall. There is a balance that needs to be established in asking questions horizontally (in order) and vertically (according to shock).
• There were some questions that the respondents found particularly difficult to answer. Follow-up and sending specific questions in advance are crucial in ensuring completeness of the responses.
• Two enumerators are ideal in the interview setup. One would be tasked with asking the questions with the right sequence in mind. The other is in charge of writing down responses, asking follow-up and clarifying questions, and ensuring completeness of the questionnaire.
• Show cards with the codes for responses are very important in ensuring proper comprehension and recall. Even with extensive deliberation, each DRRM official may have a distinct interpretation of the question. Readily providing the available responses would mitigate differences in interpretation.

### 2.4 Reading the data

Supplementary Appendix A [1] provides the data file with accompanying data dictionary (in .txt or .dct format, the contents are the same). From the results of the face-to-face survey, an encoding program was developed using MS Access to electronically capture the data from the survey. The encoding program looks exactly the same as the paper survey questionnaire to mitigate errors in encoding. The encoded data via MS Access were then exported into Microsoft Excel. Finally, data output from the different encoders were merged using the Stata software. The data is converted as comma-separated values file (.csv) for general accessibility.

The user of the data is guided by the data dictionary in navigating the data file that is organized in seven blocks (Table 2). Block A are data on the profile and characteristics of the DRRM officers and their respective local government units. Blocks A1 and A2 on the general information on the municipality, DRRM officer, and DRRM office. Blocks A3 and A4 are on training and assets, respectively, which are specific to the municipality and the disaster officer. Block B covers the incidence of shocks, related damages and state of recovery. Block C on ex-ante public controls, block D on ex-post loss reduction, and block E on coping mechanisms or the policy tools available to the municipality in mitigating the adverse effects due to the natural hazard or shock. Block F on risk perception, which aims to capture the risk profile of the disaster officer with regard to his municipality. Block G covers agriculture related questions, the primary sector in many municipalities and cities in the country. The last set of variables are the computed sub-indices.

**Table 2:** Coverage of the survey questionnaire and data.

	BLOCK	DESCRIPTION/COVERAGE
A1	Profile	- Profile of the municipality and the DRRM office - Geographical and economic characteristics of municipality - Demographic characteristics of officer
A2	DRRM Plan and Budget	- Information on the DRRM office and budget - Date of establishment, personnel, programmed budget, presence of contingency and land-use plans
A3	DRRM Trainings	- Disaster-related trainings received and conducted by the DRRM - Number, type, and source of DRRM trainings
A4	Assets	- Inventory of disaster management assets - By usage: vehicles, equipment, and supplies
B1	Incidence of Shocks	- Incidence of shock(s) - Incidence of experiencing a shock (since January 2009, since 1980), number of times, details on most severe shock: dates, severity, number of families affected - Covers climatological, metrological, geophysical, hydrological, and biological hazards
B2	Damages	- Damages from the shock(s) that hit the area - Amount segregated by sectors, types of damage
B3	State of Recovery	- Recovery from the shock(s) that hit the area - Scale, description, and length of recovery
C1	Controls: Ex-Ante Reduction of Exposure	- Short-term, mid-term, and long-term harm mitigation activities implemented by the city/municipality - Activity, importance to recovery, time and length of implementation
C2	Early Warning and Response	- Warnings received and issued by the city/municipality in relation to the shock(s) - Time, source, medium, checklist of preparatory measures
D1	Ex-Post Loss Reduction: Evacuation	- Conduct of evacuations - Coverage and time, compliance - Evacuation center number, facilities, and original use
D2	Ex-Post Loss Reduction: Search and Rescue	- Conduct of search and rescue operations - Time and length of search and rescue - Death, illnesses, and injuries
D3	Ex-Post Loss Reduction: State of Calamity	- Activities carried out by the DRMM office after declaration of State of Calamity - Declaration, usage of quick response fund and calamity funds, source agencies, insurance, price freeze
D4	Ex-Post Loss Reduction: Relief	- Assistance provided by the DRMM office to the constituents to help cope with the shock(s) - Type, time, and length of relief given
D5	Ex-Post Loss Reduction: Response from Others	- Assistance provided by government agencies and other LGUs in response to the shock(s) - Type, time, and length of assistance provided.
E1	Coping: Clean-Up Operations	- Conduct of cleanup operations - Time, length and amount used in cleanup
E2	Coping: Employment	- Effect of the shock(s) on employment and housing - Cash-for-work and food-for-work programs
E3	Coping: Loans	- Loans applied for as additional funding to cope with the shock(s) - Amount, source, and timing of loans
E4	Coping: Rehabilitation of Lifeline Services	- Impact of the shock(s) on electricity, water, and telecommunication services. - Time, length, and coping activities
E5	Coping: Rebuilding and reconstruction	- Spending of the city/municipality as a result of the shock(s) - Type, and amount of infrastructure damaged - Loss of records, files, or data
E6	Coping: Housing and Relocation	- Effect of the shock(s) on housing - Temporary and permanent movement - Housing programs started after shock(s)
F	Risk Perception	- Perception on the likelihood of shock(s) happening in the future - Perception of incidence, exposure, and preparation of municipality to future shocks
G	Harm Mitigation: Agriculture and Fisheries	- Impact of the shock(s) on agriculture - Amount and type of product or asset - Agricultural support programs
	Sub-indices	- Sub-indices as described in Fig. 2, Table 7, Supplementary Appendix C [1].

### 2.5 General results

Table 3 provides the profile of the 193 municipalities sampled, in terms of industry. Note that the primary industries are agriculture, fisheries, tourism, and construction. The majority (62%) of the municipalities and cities interviewed also have a sister city, and 68% of the municipalities have DRRM activities with their sister municipalities. Sister city/municipalities means that two LGUs have some form of agreement that they will be supporting each other in time of needs or their respective undertakings. This indicates a high level of implementation with regard to DRRM activities.

**Table 3:** General profile of sampled municipalities and cities.

		Yes	No	TOTAL
Do you have the following industries in your city/municipality?	Agriculture	178	15	193
		(92.23)	(7.77)	(100)
	Fisheries	140	53	193
		(72.54)	(27.46)	(100)
	Construction	112	81	193
		(58.03)	(41.97)	(100)
	Heavy Industry	47	146	193
		(24.35)	(75.65)	(100)
	Tourism	123	70	193
		(63.73)	(36.27)	(100)
	Mining	28	165	193
		(14.51)	(85.49)	(100)
	Services	96	97	193
		(49.74)	(50.26)	(100)
	Others (1)	12	181	193
		(6.22)	(93.78)	(100)
	Others (2)	1	192	193
		(0.52)	(99.48)	(100)
Does your city/municipality have a sister-city/town arrangement, either formal or informal, with another city/municipality/province?		119	74	193
		(61.66)	(38.34)	(100)
Is DRRM assistance included in that partnership?		81	38	119
		(68.07)	(31.93)	(100)

Table 4 presents the profile of the DRRM offices included in the survey. The majority of the respondents have a DRRM office established, although some of them are informally housed in different departments. A number of municipalities have developed a proper DRRM plan according to the provided guidelines within the law. Only 6 out of 193 municipalities have no Land Use Plan, and only 10 have no contingency plan in case of a severe natural hazard. Table 5 presents the incidence of experiencing disasters.

**Table 4:** Profile of local DRRM offices.

		Yes	No	TOTAL
**DRRM Information**	Does your city/municipality have a DRRM office?	183	10	193
		(94.82)	(5.18)	(100)
**DRRM Plan**	Does your city/municipality have a DRRM plan?	191	2	193
		(98.96)	(1.04)	(100)
	Conducted vulnerability assessments	185	6	191
		(96.86)	(3.14)	(100)
	Presented vulnerability assessments	172	19	191
		(90.05)	(9.95)	(100)
	Consolidated programs and projects related to DRRM	184	7	191
		(96.34)	(3.66)	(100)
	Created a roadmap of planned activities	176	15	191
		(92.15)	(7.85)	(100)
	Conducted a review of the financial viability of plan	159	32	191
		(83.25)	(16.75)	(100)
	Does your city/municipality have a Comprehensive Land-use Plan?	187	6	193
		(96.89)	(3.11)	(100)
	Does your city/municipality have a Contingency Plan?	183	10	193
		(94.82)	(5.18)	(100)
**DRRM Budget**	Does your city/municipality have an annual DRRM fund?	186	7	193
		(96.37)	(3.63)	(100)
**City/ Municipality Capacity Bond or Insurance**	Are you aware of any catastrophe bond or insurance for your city/ municipality?	42	151	193
		(21.76)	(78.24)	(100)
	Has your LGU availed of any catastrophe bond or insurance?	17	25	42
		(40.48)	(59.52)	(100)
	Are you considering availing any catastrophe bond or insurance	63	107	170
		(37.06)	(62.94)	(100)

**Table 5:** Number of times disaster has been experienced since 2009/1980.

Disaster	Obser-vation	Mean	SD	Min	Max
**Caused by hydrometeorogical hazards**					
Combined hydro-meteorological	189	3.667	6.870	1	60
Strong winds and rain	167	3.605	6.620	1	60
Flood due to continuous rain, storms, etc.	147	3.340	5.745	1	42
Landslide/mudslide	46	2.630	4.720	1	27
Big waves (including tsunami and storm surge)	31	1.677	1.759	1	8
**Others**					
Drought (El Niño)	65	1.292	0.824	1	5
Biological hazards (i.e. leptospirosis)	16	1.438	1.750	1	8
Pest infestation, crop diseases	28	1.571	1.476	1	7
Earthquake (shaking of earth)	41	1.122	0.400	1	3
Volcanic eruption	20	1.200	0.523	1	3

Different municipalities experience different sets of shocks, and not all municipalities face the same shocks. Table 6 illustrates this for the four hydrometeorological hazards – typhoons, floods, landslides, and tsunamis. Many hydrometeorological hazards are frequently connected to the same disaster event – e.g. a typhoon in low-lying areas typically causes floods, and if they are near the coast, tsunamis. This is also reflected in the survey responses, wherein most municipalities have the same set of responses due to typhoons, floods, landslides, and tsunamis when the disaster event behind the different shocks coincides. In order to capture this in the dataset, a variable for multiple shocks were created based on the prevalence and severity of each type of shock. The combined variable prioritized the inclusion of strong winds and rains due to the relative completeness of the responses in this section.

**Table 6:** Number of municipalities experiencing several disasters.

Shocks/Disasters	Observation	Percent
1,2,3,6	10	5.18
1,2,3	26	13.47
1,2,6	15	7.77
1,2	76	39.38
1,3,6	1	0.52
1,3	5	2.59
1,6	5	2.59
1	29	15.03
2	20	10.36
3	2	1.04
(none)	4	2.07
Total	193	100.00

Note: Shocks/Disasters code (1) strong winds and rains, (2) flood due to continuous rains, storms, (3) landslide/mudslide, (4) big waves including tsunami and storm surge, (5) drought, (6) biological hazards.

### 2.6 Disaster risk management sub-indices

To capture the various aspects of related information in one single variable, we develop sub-indices based on the blocks in the questionnaire presented in Table 2. The sub-indices serve two important functions. One, they serve as the primary variables in analyzing how various ex-post and ex-ante programs affect different post-disaster outcomes in the city/municipality. Two, they can highlight any anomalies in the dataset, and thus preserve the integrity and consistency of the dataset.

We focus on the hydrometeorological hazards, namely: strong winds and rains, floods, landslides, and big waves, that have elevated into a disaster from the perspective of the DRRM officers. Using these four disaster indicators in the dataset, we identify LGUs that experienced at least one of the hydrometeorological hazards, which became a disaster. This *combined disaster* indicator variable serves as the important identifier in our index computations. Users of this data, can follow the same methodology we presented here to create indices related to geologic hazards.

The different ex-post and ex-ante activities were grouped to form Weighted Disaster Risk Management (DRM) Sub-indices (and Non-weighted DRM Sub-indices). The former, which consists of 17 indices, is developed based on the survey questions on ex-ante reduction of exposure, ex-post loss reduction, and coping activities of the LGUs. Meanwhile, the latter, which comprises 2 indices, is based on the LGUs’ ownership of assets related to disaster work and trainings received by the DRM officials and staff. All in all, there are 19 sub-indices. These are the indices utilized in the research article [2] and [3].

• *Weighted Disaster Risk Management (DRM) Sub-indices*

We discuss the formulation of the weights assigned to the four hydrometeorological disasters. We then show how the DRM sub-indices were computed using an example. Formulas for all the 19 sub-indices are provided in Supplementary Appendix C [1].

Among the 193 sample municipalities, 189 experienced disasters caused by severe hydrometeorological hazards. We use unequal weights based on the number of LGUs that experienced disasters from a particular hydrometeorological hazards to give a greater weight towards preparedness on the disaster that many local government units experienced. Among the 189 LGUs that experience the most severe hydrometeorological disasters, the distribution of those affected are as follows: Strong winds & rain – 167; Floods – 20; Landslide – 2; and Big waves – 0. Hence, the weight assignment of each disaster are as follows:

$$w_{1}=\frac{167}{189},\;w_{2}=\frac{20}{189},\;w_{3}=\frac{2}{189}\;\text{and}\;w_{4}=0$$

where $w_{1}$ is the weight for strong winds & rain;$\;w_{2}$ is the weight for floods; $w_{3}$ is the weight for landslide; and $w_{4}$ is the weight for big waves. This weighting method gives more importance on the preparedness of the local government units on the hazards that many of them experienced, i.e. strong winds and rain.

In computing for the DRM sub-indices, we first identify the sub-component indices from the blocks on ex-ante reduction of exposure, ex-post loss reduction, and coping in the questionnaire. Next, we combine the sub-component indices to form the component index of each hydrometeorological disaster. We notice that the variables under consideration have compounding effect. Take note that in cases like this, while it is convenient, the arithmetic mean is inaccurate in this case. The geometric mean should be employed to account for compounding effects over time and it is “not overly influenced by the very large values in a skewed distribution (Spizman & Weinstein [12]; Kirkwood & Sterne [13]). Using geometric mean, we combine the component indices to form the DRM sub-indices of each hydrometeorological disaster. When the need for scale effects are taken into account, the index is computed using geometric means.

Finally, using a weighted simple averaging method, we combine the component indices of the four hydrometeorological disaster to form the weighted DRM sub-indices. Note that adjusting for scale effects is not necessary since the components are calibrated between 0 and 1.

To illustrate how the weighted DRM sub-indices are computed, we show the computation for the Weighted Precautionary Measures Index - Short-Term ($\bar{PMIST}$), which captures short-term precautionary activities, such as class suspension, gale warning, road closures undertaken by the LGUs.

First, we identified the following sub-components for the $\bar{PMIST}$, namely: 1) time of implementation; and 2) length of implementation. Denote $PMIST_{i,s}$ as Precautionary Measure Index - Short-Term of $i^{\text{th}}$ LGU for $s^{\text{th}}$ type of hydrometeorological disaster.

Next, we calculate the index for each hydrometeorological hazard (strong winds & rain, flood, landslide and big waves).

(1) $$PMIST_{i,s}=\sqrt{\frac{\sum_{j=1}^{3}PMST_{ij,s}\times TI_{ij,s}}{{\underset{︸}{3\;\times \;5}}_{time\;of\;implementation}}\times \frac{\sum_{j=1}^{3}PMST_{ij,s}\times LI_{ij,s}}{{\underset{︸}{3\;\times \;4}}_{length\;of\;implementation}}}\times 100\%$$

where:

$PMST_{ij,s}$≡ Indicator variable for the $j^{\text{th}}\;$type of short-term precautionary measure activities conducted by $i^{\text{th}}$ LGU for $s^{\text{th}}$ type of hydrometeorological disaster (1 – Yes, 0 – No);
$TI_{ij,s}$≡ Ordinal variable for the time of implementation of $j^{\text{th}}\;$type of short-term precautionary measure conducted by $i^{\text{th}}$ LGU for $s^{\text{th}}$ type of hydrometeorological disaster ≡ {1 - more than 24 h before the disaster, 2- 24 h or less before the disaster, 3 – during the disaster, 4 - less than 24 h after the disaster, 5 - more than 24 h after disaster};
$LI_{ij,s}$≡ Ordinal variable for the length of implementation of $j^{\text{th}}\;$type of short-term precautionary measure conducted by $i^{\text{th}}$ city/municipality for $s^{\text{th}}$ type of hydrometeorological disasters ≡ {1 – less than 1 day, 2 – 1 to 3 days, 3 – 4 days to 1 week, 4 – more than 1 week to 1 month};
*i*≡ LGU ≡ 1, 2,…189;
*j*≡ types of short-term precautionary measure ≡ {1- class suspension, 2- gale warning, 3- road closures};
*s*≡ type of hydrometeorological hazard ≡ {1- Strong winds & rain, 2 - Flood, 3 - Landslide, 4 – Big waves}

Finally, we obtain the $\bar{PMIST}$ using weighted simple averaging method of the four hydrometeorological hazards,

(2) $${\bar{PMIST}}_{i}=\left(w_{1}\;PMIST_{i,1}+w_{2\;}PMIST_{i,2}+w_{3\;}PMIST_{i,3}+w_{4\;}PMIST_{i,4}\right)\;\times \;100\%$$

$\bar{PMIST}$ is the weighted DRM sub-index used to measure an LGUs ex-ante reduction exposure. This index is calibrated between 0 and 1, where perfect compliance of an LGU to all short-term measures activities before a disaster occurs is indicated by a value of 1. A value of 0 means a municipality does not conduct any short-term precautionary measure activities.

• *Non-Weighted Disaster Risk Management (DRM) Sub-indices*

Non-disaster specific indices are created to measure the DRRM trainings received and conducted by LGUs and their inventory of disaster management assets that help them prepare and respond to disasters caused by natural hazards. We created two Non-Weighted DRM sub-index, Trainings Index (TI) and Asset Index (AI). These Non-Weighted DRMS are only average of their components and computed similar to Eq. (1).

**Fig. 2:**
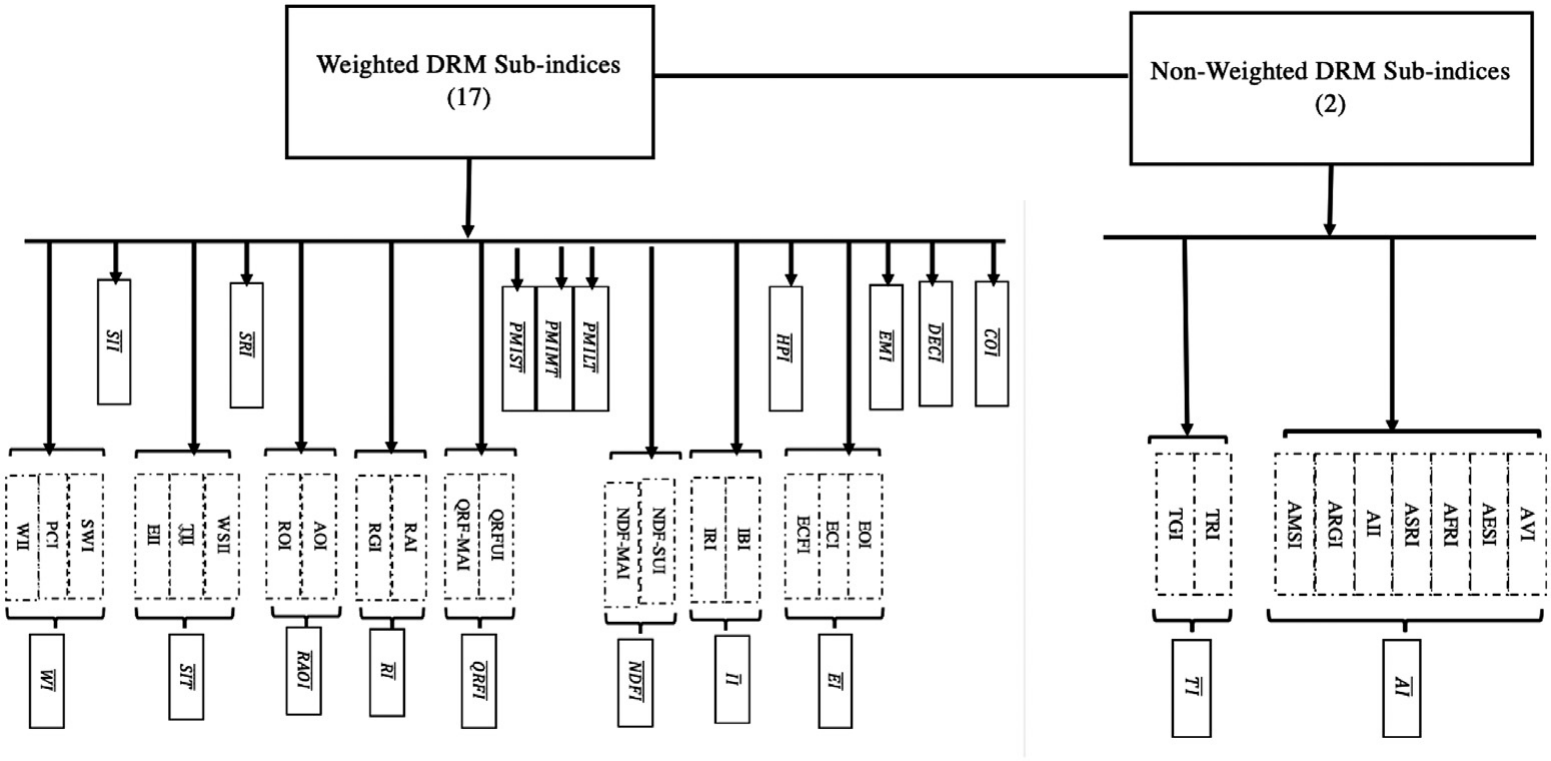
Development of Disaster Risk Management (DRM) Sub-Indices Note: Combined disaster indicator refers to the four hydrometeorological disasters due to strong winds and rain, flood, landslide, and big waves. See also Table 7 and Supplementary Appendix C [1].

The fourteen weighted DRM sub-indices and two non-weighted DRM sub-indices are summarized in Fig. 2 and described in Table 7. There are nineteen sub-indices utilized in the research article [2] and [3]. The full sets of equations are in Supplementary Appendix C [1].

**Table 7:** List of disaster risk management sub-indices.

No.	Index Name	Brief Description
1	Assets Index ($\bar{AI}$)	Geometric mean of 7 indices: 1.) Asset Vehicle Index (AVI), 2. Asset Emergency Shelter Index (AESI), 3. Asset Facilities and Resources Index (AFRI), 4. Asset Search and Rescue Index (ASRI), 5. Asset Information Index (AII), 6. Asset Relief Goods Index (ARGI), and 7.) Asset Medical Supplies Index (AMSI).
	*1.1. Asset Vehicle Index (AVI)*	Mean of the type of vehicles used by the municipality in times of hydrometeorological hazards (boats, vans, truck, bus, ambulance, amphibian, backhoe or scoop loader, dump truck, firetruck, crawler and tractor) and the frequency of use.
	*1.2. Asset Emergency Shelter Index (AESI)*	Mean of the type of emergency shelter supplies given by the municipality in times of hydrometeorological hazards (jackets or raincoats, clothes, beds, beddings (blankets, etc.), mosquito nets and kitchen supplies) and the frequency of distribution.
	*1.3. Asset Facilities and Resources Index (AFRI)*	Mean of the type of facilities and resources used by the municipality in times of hydrometeorological hazards (Portable/Solar-powered generator and Mobile water treatment) and the frequency of use.
	*1.4. Asset Search and Rescue Index (ASRI)*	Mean of the type of search and rescue equipment used by the municipality in times of hydrometeorological hazards (Siren, megaphone, or whistle, Two-way radio, GPS device, Ropes, Search light or flashlight, Ladders, Helmets, Life vest/ reflectorized vest, Extraction kit (spine board, shovel, jackhammer) and Caution tape) and the frequency of use.
	*1.5. Asset Information Index (AII)*	Mean of the type of information and awareness equipment used by the municipality in times of hydrometeorological hazards (Phones, Laptops, Internet connection, Batteries and Power banks) and the frequency of use.
	*1.6. Asset Relief Goods Index (ARGI)*	Mean of the type of relief goods distributed by the municipality in times of hydrometeorological hazards (Bottled water, Rice, Noodles, Canned goods, Ready-to-eat meals, Milk for infants, Toothbrush, Toothpaste, Soap, Shampoo, Sanitary pad and Diaper) and the frequency of distribution.
	*1.7. Asset Medical Supplies Index (AMSI)*	Mean of the type of medical supplies distributed by the municipality in times of hydrometeorological hazards (First-aid kits, Vaccines, Cadaver bags, Disinfectants and Antiseptics, Dressings (for wounds), Surgical Instruments, Thermometers, Stethoscope, Sphygmomanometer (for blood pressure), Gloves and Surgical Masks, Syringes and Needles, and Plastic bags) and the frequency of distribution.
2	Cleanup Operations Index ($\bar{COI}$)	Weighted geometric mean of these two components: 1. Product of the indicator variable if the LGU has undertaken clean-up operations and when it started, and 2. Product of the indicator variable if the LGU has undertaken clean-up operations and duration.
3	Disaster Effects to Constituents Index ($\bar{DECI}$)	Weighted arithmetic means of the product of the indicator variable if the hydrometeorological hazard resulted in death, illness or injury of the constituents and the types of effects (Death, Illness and Injury).
4	Employment Index ($\bar{EMI}$)	Weighted geometric mean of two components: 1.) Product of the indicator variable if the LGU has a cash-for-work program for the hydrometeorological hazard and the daily wage rate (Less than Php 150, Php 150–300, Php 301–450, Php 451–600 and More than Php 600); and 2.) Product of the indicator variable if the LGU has a food-for-work program for the hydrometeorological hazard and the value of the food for a day's work (Less than Php 150, Php 150–300, Php 301–450, Php 451–600 and More than Php 600).
5	Evacuation Index ($\bar{EI}$)	Weighted geometric mean of three indices: 1.) Evacuation Order Index (EOI), 2.) Evacuation Center Index (ECI), and 3.) Evacuation Center Facilities Index (ECFI).
	*5.1. Evacuation Order Index (EOI)*	Mean of product of presence of evacuation order issued to the constituents and time it was issued.
	*5.2. Evacuation Center Index (ECI)*	Mean of the product of presence of evacuation center designated for the hydrometeorological hazard and their original use (Public school building, Public gym/basketball court, etc., Municipal hall, Church, and Private building)
	*5.3. Evacuation Center Facilities Index (ECFI)*	Mean of the product of presence of evacuation center designated for the hydrometeorological hazard and the facilities available (Toilets, Generators, Common Kitchen, Health Station and Assembly Area)
6	Housing Program Index ($\bar{HPI}$)	Weighted geometric mean of the indicator variable if the LGU has any housing programs in response to the hydrometeorological hazard and when it was started.
7	Infrastructure Index ($\bar{II}$)	Weighted geometric mean of two indices: 1.) Infrastructure Breakdown Index (IBI), and 2.) Infrastructure Repair Index (IRI).
	*7.1. Infrastructure Breakdown Index (IBI)*	Mean of the product of the indicator variable if the LGU had infrastructure breakdowns during/ after the hydrometeorological hazard and the types of infrastructures that broke down (DRRM office, Municipal hall, Health office, health center, Public school, Public gym, Other government-owned buildings, Public equipment, Public vehicles, Bridges, roads, Water facilities, Electrical facilities and Communication facilities (e.g. cell sites)).
	*7.2. Infrastructure Repair Index (IRI)*	Weighted geometric mean of two components: 1. Product of the indicator variable if the LGU had infrastructure breakdowns during/ after the hydrometeorological hazard, indicator variable if the damage was fixed and length of repair; and 2. Product of the indicator variable if the LGU had infrastructure breakdowns during/ after the hydrometeorological hazard and the agencies that funded the repair (Own city/municipality, DSWD, DILG, DOH, DepEd, DA, DPWH, AFP-OCD, BFP, Coast Guard, and PNP).
9	National Disaster Fund Index ($\bar{NDFI}$)	Weighted geometric mean of two indices: 1.) National Disaster Fund Sources & Uses Index (NDFSUI); and 2.) National Disaster Fund Monetary Assistance Index (NDF-MAI).
	*9.1. NDF Sources and Uses Index (NDF-SUI)*	Geometric mean of two components: 1.) Product of the indicator variable if the LGU availed the NDF for the hydrometeorological hazard and the indicator variable for the agency that released the fund; and 2.) Product of the indicator variable if the LGU availed the NDF for the hydrometeorological hazard and the indicator variable if the LGU availed the NDF for the hydrometeorological hazard and the uses of the fund (Search and Rescue, Relief Goods Procurement, Soup Kitchen, Other Relief Operations, Clean-up Operations, Restoration of Lifeline Services, Employment and Livelihood, Housing & Relocation, Reconstruction of damaged buildings, Replacement & Repair of Lost Assets, Monetary Assistance, etc.)
	*9.2. NDF Monetary Assistance Index (NDF-MAI)*	Geometric mean of two components: 1. Product of the indicator variable if the LGU availed funding from the National Disaster Fund/ Calamity Fund and the indicator variable if the fund was used for monetary assistance; and 2. Product of the indicator variable if the fund was used for monetary assistance and types of monetary assistance offered (Emergency shelter, livelihood, health, unconditional, etc.).
9	Precautionary Measures Index - Long Term ($\bar{PMILT}$)	Weighted mean of the product of type of long-term precautionary measures conducted by an LGU and its length of implementation. These measures include: Build resilient housing units, Invest In stronger public facilities, Build (cement) dams, dikes and river embankments, Upgrade power and water lines, Major road repairs, Identify relocation areas, Rezoning and land-use regulations, Build drainage, among others.
10	Precautionary Measures Index - Mid-term ($\bar{PMIMT}$)	Weighted mean of the product of type of mid-term precautionary measures and its frequency of implementation. These measures include: Assess the safety of public buildings, Strengthen river embankments and dikes using sandbags, Clean sewers and canals, Conduct road assessment and repairs, Repair/rehabilitate classrooms, etc.
11	Precautionary Measures Index - Short Term ($\bar{PMIST}$)	Weighted geometric mean of the type of short-term precautionary measures, its time of implementation, and its length of implementation. These measures include the following: Class suspension, Gale warning, Road closures, etc.
12	QRF Index ($\bar{QRFI}$)	Weighted geometric mean of two indices: 1.) Quick Response Fund Uses Index (QRFUI); and 2.) Quick Response Fund Monetary Assistance Index (QRF-MAI).
	*12.1. QRF Uses Index (QRFUI)*	Average of the product of the indicator variable if the LGU used its Quick Response Fund (QRF) and the types of fund use (Search and Rescue, Relief Goods Procurement, Soup Kitchen, Other Relief Operations, Clean-up Operations, Restoration of Lifeline Services, Employment and Livelihood, Housing and Relocation, Reconstruction of damaged buildings, Replacement and Repair of Lost Assets, and Monetary Assistance)
	*12.2. QRF Monetary Assistance Index (QRF-MAI)*	Geometric mean of two components: 1. Product of the indicator variable if the LGU used its Quick Response Fund (QRF) and indicator variable for QRF monetary assistance; and 2. Product of the indicator variable if the LGU used its Quick Response Fund (QRF) and the types of monetary assistance given by the LGU (Emergency Shelter, Livelihood, Health and Unconditional)
13	Relief Index ($\bar{RI}$)	Weighted geometric mean of two indices: 1.) Relief Assistance Index (RAI); and 2.) Relief Goods Index (RGI).
	*10.1. Relief Assistance Index (RAI)*	Geometric mean of two components: 1. Product of the indicator variable for presence of relief assistance to the constituents extended by the LGU and the type of relief assistance provided (Soup kitchen, Emergency shelter kit, Relief goods (e.g. food pack and water), and Medical kit); and 2. Product of the indicator variable for presence of relief assistance to the constituents extended by the LGU and when it was provided.
	*10.2. Relief Goods Index (RGI)*	Geometric mean of two components: 1. Product of the indicator variable for presence of relief assistance to the constituents extended by the LGU and indicator variable for relief goods assistance; and 2. Product of the indicator variable for relief goods assistance and duration of relief provided.
14	Response & Assistance from Others Index ($\bar{RAOI}$)	Weighted geometric mean of two indices: 1. Response from Others Index (ROI); and 2. Assistance from Others Index (AOI).
	*14.1. Response From Others Index (ROI)*	Average of the product of the indicator variable for presence of assistance extended by other government agencies, LGUs or NGOs after the hydrometeorological hazard and the agencies that provided assistance (DSWD, DILG, DOH, DepEd, DA, DPWH, AFP-OCD, BFP, Coast Guard, PNP, Other agency, Other city, Other province, Local NGOs and Foreign NGOs)
	*14.2. Assistance from Others Index (AOI)*	Weighted geometric mean of two components: 1. Product of the indicator variable for presence of assistance extended by other government agencies, LGUs or NGOs after the hydrometeorological hazard and the types of assistance; and 2. the product of the types of assistance (Search and Rescue, Relief Goods, Soup Kitchen, Other Relief Operations, Clean-up Operations, Restoration of Lifeline Services, Employment and Livelihood, Housing and Relocation, Reconstruction of damaged buildings, Replacement and Repair of Lost Assets, and Monetary Assistance) and duration of assistance provided.
15	Search and Rescue Index ($\bar{SRI}$)	Weighted arithmetic mean of the product of the indicator variable if the LGU conducted search & rescue and the ordinal variable for no. of people rescued (Less than 100 people, 101 to 200 people, 201 to 300 people, 301 to 400 people, 401 to 500 people, More than 501 people)
16	Service Interruption Index - Type ($\bar{SIT}$)	Weighted geometric mean of three indices: 1.) Water Supply Interruption Index (WSII), 2.) Telecommunication Interruption Index (TII), and 3.) Electricity Interruption Index (EII).
	*16.1. Water Supply Interruption Index (WSII)*	Geometric mean of two components: 1. Product of the indicator variable if the LGU had water supply interruption during the hydrometeorological hazard and when the water supply was cut-off; 2. Geometric mean of the indicator variable if the LGU had water supply interruption during the hydrometeorological hazard, length of interruption and stop-gap measures utilized (Rationing, Water Wells and Mobile Water Treatment).
	*16.2. Telecommunication Interruption Index (TII)*	Geometric mean of two components: 1. Product of the indicator variable if the LGU had telecommunication interruption during the hydrometeorological hazard and when the telecommunication service was cut-off; and 2. Mean of the product of the indicator variable if the LGU had telecommunication interruption during the hydrometeorological hazard, length of interruption and stop-gap measures utilized (Satellite phone and Two-way radio).
	*16.3. Electricity Interruption Index (EII)*	Geometric mean of two components: 1. Product of the indicator variable if the LGU had electricity interruption during the hydrometeorological hazard, when the electricity service was cut-off; and 2. Mean of the indicator variable if the LGU had electricity interruption during the hydrometeorological hazard, length of interruption and stop-gap measures utilized (Gas or Diesel-powered generators and Solar panels).
17	Service Interruption Index ($\bar{SII}$)	Weighted geometric mean of the indicator variable if the LGU had any service interruption during the hydrometeorological hazard and the types of service interruption (Water, Telecommunication and Electricity).
18	Trainings Index ($\bar{TI}$)	Geometric mean of two indices: 1.) Trainings Given Index (TGI), and 2.) Trainings Received Index (TRI).
	*18.1. Trainings Received Index (TRI)*	Mean of the type of training received by different individuals/ institutions and the type of training received by the municipality. The type of trainings are as follows: Prevention and Mitigation, Information and Awareness, Evacuation, Early Warning, Search and Rescue, Relief and Recovery
	*18.2. Trainings Given Index (TGI)*	Mean of the type of training given to different individuals/ institutions and the type of training conducted by the municipality. The types of training are the same as in TRI.
19	Warning Index ($\bar{WI}$)	Weighted geometric mean of three indices: 1.) Source of Warnings Index (SWI), 2.) Preparatory Checks Index (PCI) and 3.) Warning Issued Index (WII).
	*19.1. Source of Warning Index (SWI)*	Mean of the product of presence of received warning before the hydrometeorological hazard occurred and the sources of warning, which includes: PAGASA/DOST, Provincial DRRMO, NDRRMC, Local media, Other government agency, etc.
	*19.2. Preparatory Checks Index (PCI)*	Mean of the product of presence of preparatory checks conducted after receiving / hearing the warning and the kinds of preparatory checks conducted, which are the following: Check inventory of supplies and equipment, Check capacity of critical facilities like hospitals, Organize DRRM teams and personnel, Enlist volunteers, Prepare evacuation centers, and Prepare and preposition relief goods.
	*19.3. Warnings Issued Index (WII)*	Geometric mean of two components: 1. Product of presence of warning to the constituents and when it was issued; and 2. Product of presence of warning to the constituents and via what medium (Television, Radio, SMS / Calls and Social media (e.g. Facebook, Twitter, etc.)

## Appendix. Supplementary Materials

Supplementary Appendix A1 DIB Ravago et al. 2020.csv
Supplementary Appendix A2 DIB Ravago et al. 2020.txt
Supplementary Appendix A3 DIB Ravago et al. 2020.dct (same content as in the .txt file)
Supplementary Appendix B DIB Ravago et al. 2020 questionnaire.pdf
Supplementary Appendix C DIB Ravago et al. 2020 sub-index equations.pdf

## Adherence to Ethical Requirements

The authors certify that the conduct of this research has fulfilled the technical requirements necessary to demonstrate the use of ethical procedures in researching human participants. Implicit informed consent has been obtained from the participants because they have agreed to be interviewed. The respondents are public officials and public servants. The information requested from them are related to their work and activities of the local public office. No personal identifiable information of the respondents is included in the data.

## Declaration of Competing Interest

The authors declare that they have no known competing financial interests or personal relationships which have, or could be perceived to have, influenced the work reported in this article.

## Appendix Supplementary materials

Image, application 1
