# Supplementary material for: Data from a survey of the Philippines’ local governments on their risk management strategies to natural disasters
Source: Data Brief. 2020 Nov 19;33:106548. doi: 10.1016/j.dib.2020.106548 (PMC7701180; doi:10.1016/j.dib.2020.106548)
Supplement: Supplementary file 1 [file mmc1.zip › Supplementary Appendix B DIB Ravago et al 2020 questionnaire.pdf]

Supplementary Appendix B: Ravago, Majah-Leah; Mapa, Claire Dennis; Sunglao, Jun Carlo; Aycardo, Angelie Grace (2020), Data in Brief.

	BLOCK	DESCRIPTION	PAGE
	**-**	Front page	2
**A**	**Profile (Part 1)**	Profile of the city/municipality and the LDRRMO officer (Part 1)	3
**B**	**LDRRM Plan and Budget**	Information on the LDRRMO office and budget	4
**C**	**Incidence of Shocks**	Incidence of shock(s)	5
**D**	**Damages**	Damages from the shock(s) that hit the area	6
**E**	**State of Recovery**	Recovery from the shock(s) that hit the area	7
**F**	**Harm Mitigation (Ex-Ante Reduction Of Exposure)**	Short-term, mid-term, and long-term harm mitigation activities implemented by the city/municipality	8-9
**G**	**Preparedness (Early Warning And Response)**	Warnings received and issued by the city/municipality in relation to the shock(s)	10
**H**	**Response (Ex-Post Loss Reduction): Evacuation**	Conduct of evacuations	11
**I**	**Response (Ex-Post Loss Reduction): Search And Rescue**	Conduct of search and rescue operations	12
**J**	**Response (Ex-Post Loss Reduction): State Of Calamity**	Activities carried out by LDRRMO after declaration of State of Calamity	13-14
**K**	**Recovery (Coping): Relief**	Assistance provided by the LDRRMO to the constituents to help cope with the shock(s)	15
**L**	**Recovery (Coping): Clean-Up Operations**	Conduct of clean-up operations	16
**M**	**Recovery (Coping): Employment**	Effect of the shock(s) on employment and housing	16
**N**	**Recovery (Coping): Response From Others**	Assistance provided by government agencies and other LGUs in response to the shock(s)	17
**O**	**Recovery (Coping): Loans**	Loans applied for as additional funding to cope with the shock(s)	18
**P**	**Rehabilitation (Coping): Lifeline Services**	Impact of the shock(s) on electricity, water, and telecommunication services.	19-20
**Q**	**Reconstruction (Coping)**	Spending of the city/municipality as a result of the shock(s)	21
**R**	**Reconstruction (Coping): Housing And Relocation**	Effect of the shock(s) on housing	22
**S**	**Risk Perception**	Perception on the likelihood of shock(s) happening in the future	22
**T**	**Harm Mitigation: Agriculture and Fisheries**	Impact of the shock(s) on agriculture	23-24
**U**	**DRRM Trainings**	Disaster-related trainings received and conducted by the LDRRMO	25-26
**V**	**Assets**	Inventory of disaster management assets	27-28
**A-B**	**Profile (Part 2)**	Profile of the city/municipality and the LDRRMO officer (Part 2)	29

Ravago, Mapa, Sunglao, Aycardo, (2020). Mendeley Data, doi: 10.17632/9y2yhw45zt.1

NATURAL DISASTER SURVEY

CONTROL NO. ☐☐☐☐☐☐☐☐☐ — ☐ — ☐☐☐

For the enumerator only
STRICTLY CONFIDENTIAL

Geographic Information **Codes**

Region ....

Province ....

City/ Municipality ....

Disaster Risk Classification .... ☐ High-risk ☐ Low-risk

Enumerator / Interview Information

Enumerator Name (1) ....

Enumerator Name (2) ....

Date of Interview (*MM-DD-YYYY*) ☐☐—☐☐—☐☐☐☐

Time Started (*HH:MM*) ☐☐ : ☐☐

Time Ended (*HH:MM*) ☐☐ : ☐☐

Certification

We hereby certify that the data gathered in this questionnaire were obtained/reviewed by me personally and in accordance with instructions.

________________________ Signature over Printed Name of Enumerator 1

________________________ Date Accomplished

________________________ Signature over Printed Name of Enumerator 2

________________________ Signature over Printed Name of Reviewer

________________________ Date Reviewed

LDRRMO Contact Information

Address ....

....

....

Landline ....

Mobile Number ....

Email address ....

Fax No. ....

Instruction on Language

Before the interview, ask the respondent: *Which language would you like the interview to be conducted in?* Encircle language chosen below.

1 English 2 Filipino

Ravago, Mapa, Sunglao, Aycardo, (2020). Mendeley Data, doi: 10.17632/9y2yhw45zt.1

[SPIEL]: *Thank you for agreeing to participate in this survey. I will be asking some questions on shocks such as strong rains, floods, and earthquakes. Let's start with your personal profile, as well as the profile of your city/municipality.*

Salamat po sa partisipasyon ninyo sa aming survey. Magtatanong kami tungkol sa mga naranasan ninyong mga shocks gaya ng malakas na ulan, baha, at lindol. Magsimula tayo sa inyong profile at ng inyong siyudad/munisipyo.

PROFILE (Part 1)		
**A1**	**Respondent Information**	A1.1 First Name A1.2 Middle Name A1.3 Last Name A1.4 Extension *(e.g. Sr., Jr.)*
		A1.5 Age ***Edad***<br>1 18-25 years old 4 46-60 years old<br>2 26-35 years old 5 60 years old above<br>3 36-45 years old
		A1.6 Sex ***Kasarian***<br>1 Male ***Lalaki*** 2 Female ***Babae***
		A1.7 Educational Attainment<br>***Natapos na Edukasyon***<br>1 Elementary 3 College<br>2 High School 4 Post-graduate
**A2**	**DRRM Experience**	A2.1 Current Position<br>***Kasalukuyang Posisyon***
		A2.2 Years in Current Position<br>***Tagal sa Kasalukuyang Posisyon***
		A2.3 Previous Occupation/ Position<br>***Huling Posisyon o Trabaho***
**B1**	**DRRMO Level**<br>***(Encircle one)***	1 Province ***Probinsya*** 2 City ***Siyudad*** 3 Municipality ***Munisipyo*** 4 Barangay ***Barangay***
**B2**	**City/municipality information**	B2.1 Which of the following can be found in your city/municipality?<br>***Alin sa mga sumusunod ang matatagpuan sa inyong siyudad/munisipyo?*** ***(Encircle all that apply)***<br>1 Mountains ***Bundok*** 2 Volcanoes ***Bulkan*** 3 Hills ***Burol*** 4 Coast ***Tabing dagat*** 5 Rivers ***Ilog*** 6 Lakes ***Lawa*** 7 Others, specify ___________ ***Iba pa, specify*** _____________ 8 Others, specify _____________ ***Iba pa, specify*** ____________
		B2.2 Number of Municipal Employees<br>***Bilang ng mga empleyado sa munisipyo*** _________________
		**NOTE: If respondent selected codes 1 or 2, ask the AGRICULTURE section**<br>B2.3/ B2.4 Do you have the following industries in your city/municipality? *Then, ask:* Please rank each industry according to its size.<br>***Mayroon ba ng mga sumusunod na industriya ang inyong siyudad/munisipyo?*** ***(Encircle all that apply)***<br>*Then, ask:* ***Paki-rank po ang bawat industriya base sa kung gaano ito kalaki.***
		B2.3 Industry / B2.4 Rank:<br>1 Agriculture ________ 4 Heavy Industry ________ 7 Services ________<br>2 Fisheries ________ 5 Tourism ________ 8 Others, _________ ________<br>3 Construction ________ 6 Mining ________ 9 Others, _________ ________
		B2.5 Does your city/municipality have a sister-city/town arrangement, either formal or informal, with another city/municipality/province?<br>***Mayroon bang sister-city/town arrangement ang inyong siyudad/munisipyo, pormal o hindi pormal, sa ibang siyudad/munisipyo/probinsya?***<br>1 Yes 2 No ►B3.1
		B2.6 Is DRRM assistance included in that partnership?<br>***Ang DRRM assistance ba ay kasama sa partnership?*** 1 Yes 2 No

Ravago, Mapa, Sunglao, Aycardo, (2020). Mendeley Data, doi: 10.17632/9y2yhw45zt.1

B	DRRM		
B4	DRRM Plan	B4.1 Does your city/municipality have a DRRM plan? ***Mayroon bang DRRM plan ang inyong siyudad/munisipyo?***<br>1 Yes 2 No ►B4.3	
		B4.2 Please indicate which of the following has already been done by your DRRM office.<br>***Alin sa mga sumusunod ang nagawa na ng iyong DRRM office.*** ***(Encircle all that apply)***<br>1 Conducted vulnerability assessments — ***Paggawa ng vulnerability assessments***<br>2 *(If answered code 1)* Presented vulnerability assessments — ***Pagpresenta ng vulnerability assessments***<br>3 Consolidated programs and projects related to DRRM — ***Pinagsama-sama ang mga DRRM programs and projects***<br>4 Created a roadmap of planned activities — ***Paggawa ng roadmap of planned activities***<br>5 Conducted a review of the financial viability of plan — ***Paggawa ng financial viability review***	
		B4.3 Does your city/municipality have a Comprehensive Land-use Plan?<br>***Mayroon bang Comprehensive Land-use Plan ang inyong siyudad/munisipyo?***<br>1 Yes 2 No	B4.4 Does your city/municipality have a Contingency Plan?<br>***Mayroon bang Contingency Plan ang inyong siyudad/munisipyo?***<br>1 Yes 2 No
B5	DRRM Budget	B5.1 Does your city/municipality have an annual LDRRM fund?<br>***Mayroon bang taunang LDRRM fund ang inyong siyudad/munisipyo?*** 1 Yes 2 No	
		B5.2 (If yes on B5.1) How much is your annual (operating) fund?<br>***Magkano ang inyong taunang pondo?***<br>___________________________________	B5.3 How much is allocated to DRRM projects and activities?<br>***Magkano ang nakalaan sa DRRM projects and activities?***<br>___________________________________
		B5.4 How much is allocated to the Quick Response Fund?<br>***Magkano ang nakalaan sa Quick Response Fund?***<br>___________________________________	B5.5 Since January 2009, How many times was the Quick Response Fund used?<br>***Mula January 2009, ilang beses na nagamit ang Quick Response Fund?***<br>___________________________________

SPIEL: Cities/municipalities are affected by different [SHOCK]s - adverse events that can lead to illness, injury, or death; loss of livelihood, and/or public/private property damage. I want to ask you about the shocks that your city/municipaliy has experienced.

Ang mga siyudad at munisipyo ay naapektuhan ng mga [SHOCK] o mga di inaasahang pangyayari na talagang nakakapinsala. Kasama sa epekto ng mga [SHOCK] na ito ang pagkasakit, pagkasugat, or pagkamatay ng mga tao, kawalan ng hanapbuhay, at pinsala sa pampubliko o privadong property. Magtatanong ako tungkol sa mga shocks na naranasan ng inyong siyudad/munisipyo.

Ask questions for [SHOCK] code 8 (Pest infestation, crop diseases) if the respondent answered codes 1 or 2 in B2.3. [SHOCK] code 7 (Biological hazards) are only relevant to outbreaks of Leptospirosis.

C	INCIDENCE																
	C1						C2				C3	C4	C5				
Shock Code	Since January 2009, has your city/municipality experienced the following [SHOCK]? *Mula Enero 2009, nakaranas ba ang inyong siyudad/munisipyo ng alinman sa mga sumusunod na [SHOCK]?*						Since January 1980, has your city/municipality experienced the following [SHOCK]? *Mula Enero 1980, nakaranas ba ang inyong siyudad/munisipyo ng alinman sa mga sumusunod na [SHOCK]?*				How severe was the [SHOCK]? *(See code C3)*	How many families were affected by the [SHOCK]?	Since 2012, aside from the most severe [SHOCK], did you experience another [SHOCK] that is close in magnitude? *Mula 2012, maliban sa pinakamatinding [SHOCK], may iba pa ba kayong naranasan na [SHOCK] na halos pareho?*			C5.3	C5.4
	C1.1	C1.2	C1.3		C1.3	C1.4	C2.1	C2.2	C2.3				C5.1	C5.2		How severe was the [SHOCK]? *(See code C3)*	How many families were affected by the [SHOCK]?
	1 Yes<br>2 No ►C2	How many times? *Ilang beses?*	When did the most severe [SHOCK] occur? *Kailan nangyari ang pinakamatinding [SHOCK]?*		(If 1,2,6 in C1.1) Was the [SHOCK] caused by a typhoon? *Ang [SHOCK] ba ay dulot ng bagyo?* 1 Yes 2 No►C2	What was the name of the typhoon? *Anong ang pangalan ng bagyo?*	1 Yes<br>2 No ►C3	How many times? *Ilang beses?*	When did the most severe [SHOCK] occur? *Kailan nangyari ang pinakamatinding [SHOCK]?*		*Gaano katindi ang [SHOCK]? (See code C3)*	*Ilang mga pamilya ang naapektuhan ng [SHOCK]?*	1 Yes<br>2 No ►D1	When did this [SHOCK] occur? *Kailan nangyari ang [SHOCK] na ito?*		*Gaano katindi ang [SHOCK]? (See code C3)*	*Ilang mga pamilya ang naapektuhan ng [SHOCK]?*
			Month	Year					Month	Year				Month	Year		
1																	
2																	
3																	
4																	
6																	
7																	
8																	
31																	
32																	

C1.1 Codes for Shocks
- 1 Strong winds and rain
- 2 Flood due to continuous rains, storms, etc.
- 3 Landslide/mudslide
- 4 Drought (El Niño)
- 6 Big waves (including tsunami and storm surge)
- 7 Biological hazards (i.e. leptospirosis)
- 8 Pest infestation, crop diseases

C1.3/2.3/5.3 Codes for Months
- 1 January
- 2 February
- 3 March
- 4 April
- 5 May
- 6 June
- 7 July
- 8 August
- 9 September
- 10 October
- 11 November
- 12 December

C2.1 Codes for Shocks
- 31 Earthquake (shaking of earth)
- 32 Volcanic eruption

C3 Codes for Severity
- 1 Least severe
- 2 Somewhat severe
- 3 Very severe
- 4 Most severe

Ravago, Mapa, Sunglao, Aycardo, (2020). Mendeley Data, doi: 10.17632/9y2yhw45zt.1

IMPORTANT:
a) From this point onwards, responses must be based on the most severe incident of the shock since 2012 (C5). If answered code 2 or No in C5. 1, responses will be based on the most severe incident of shock since 2009 (C1 and C2).
b) This section shall be self-accomplished by the respondent. Responses in the sheet should be attached and verified by the enumerator before turnover of all fieldwork materials for quality check.

**D**	**DAMAGES**																							
	**D1**				**D2**										**D3**									
Shock Code	How severe was the damage cost by the [SHOCK]? ***Gaano katindi ang halaga ng pinsala ng [SHOCK]?*** *(See code D1. Encircle all that apply)*				How much was the cost of damage on….? / ***Magkano ang halaga ng pinsala sa…?*** *(Ask for each sub-sector below: electricity, transport, etc.)*										How much was the cost of loss on….? / ***Magkano ang halaga ng nawala sa...*** *(Ask for each sub-sector below: electricity, transport, etc.)*									
					**D2.1** **Infrastructure sectors**				**D2.2** **Economic sectors**		**D2.3** **Social Sectors**			**D2.4** **Cross-sectoral**	**D3.1** **Infrastructure sectors**				**D3.2** **Economic sectors**		**D3.3** **Social Sectors**			**D3.4** **Cross-sectoral**
					Electricity ***Kuryente***	Roads, bridges, flood control ***Daanan, tulay, flood control***	Transport ***Transport***	Water and sanitation ***Tubig at sanitasyon***	Agriculture ***Agrikultura***	Industry, Services ***Industriya, Serbisyo***	Education ***Edukasyon***	Health ***Kalusugan***	Housing ***Pabahay***	Local government ***Lokal na gobyerno***	Electricity ***Kuryente***	Roads, bridges, flood control ***Daanan, tulay, flood control***	Transport ***Transport***	Water and sanitation ***Tubig at sanitasyon***	Agriculture ***Agrikultura***	Industry, Services ***Industriya, Serbisyo***	Education ***Edukasyon***	Health ***Kalusugan***	Housing ***Pabahay***	Local government ***Lokal na gobyerno***
1	1	2	3	4																				
2	1	2	3	4																				
3	1	2	3	4																				
4	1	2	3	4																				
6	1	2	3	4																				
7	1	2	3	4																				
8	1	2	3	4																				
31	1	2	3	4																				
32	1	2	3	4																				

D1 Damage Cost
1 Suffered moderate to severe property damage
2 Families suffered moderate to severe loss of livelihood, harvest, fishing boat, etc.
3 Families suffered personal injury or loss of member of family
4 City/municipality suffered public infrastructure damage

Ravago, Mapa, Sunglao, Aycardo, (2020). Mendeley Data, doi: 10.17632/9y2yhw45zt.1

E	STATE OF RECOVERY		
	E3	E4	
		E4.1	E4.2
Shock Code	Has your city/municipality already recovered from the negative consequences of the [SHOCK]?<br><br>***Naka-recover na ba ang inyong siyudad/munisipyo mula sa mga negatibong epekto ng [SHOCK]?***<br><br>*(See code E3. Skip to F1 if answer is code 1)*	Which best describes the recovery of your city/municipality from [SHOCK]?<br>*(See code E4.1)*<br><br>***Alin ang pinakanaglalarawan ng pag-recover ng inyong siyudad/munisipyo mula sa [SHOCK]? (See code E4.1)***	How long did your city/municipality recover? (See code E4.2)<br><br>***Gaano katagal bago naka-recover ang inyong siyudad/munisipyo? (See code E4.2)***
1			
2			
3			
4			
6			
7			
8			
31			
32			

E3 Codes for Recovery
1 Not at all
2 Not much, but some
3 Much, but not completely
4 Yes, completely

E4.1 Codes for Definition
1 Better than before [SHOCK]
2 The same as before [SHOCK]
3 Worse than before [SHOCK]
4 Don't know/ no answer

E4.2 Codes for Length of Recovery
1 Less than 1 day
2 1 to 3 days
3 4 days to 1 week
4 More than 1 week to 1 month
5 More than 1 month to 6 months
6 More than 6 months to 1 year
7 More than 1 year

Ravago, Mapa, Sunglao, Aycardo, (2020). Mendeley Data, doi: 10.17632/9y2yhw45zt.1

F	HARM MITIGATION (EX-ANTE REDUCTION OF EXPOSURE)										
	F1	F2									
Shock Code	Did your city/municipality conduct precautionary measures before [SHOCK] occurred?<br>***Nagsagawa ba ng precautionary measures ang inyong siyudad/munisipyo bago ang [SHOCK]?***<br>1 Yes 2 No ►G1	**F2.1** Which of the following long-term precautionary measures?<br>***Alin sa mga sumusunod na long-term precautionary measures?***<br>**F2.2** Please rank these measures according to importance to recovery<br>*[1-Most important, 2-Next most important, and so on.]*<br>***Paki-rank ang mga measures base sa kung gaano sila ka-importante sa recovery***<br>***[1-Most important, 2-Next most important, and so on.]***<br>*(For F2.1, encircle all answers that apply in below answer grid. For F2.2, Write rank on the space provided beside the code.)*					**F2.3**<br>When were the measures implemented?<br>***Kailan pinatupad ang mga measures?***<br>*(Copy encircled codes in F2.1. For answers to F2.3, see code F2.3)*				
1		1______	2______	3______	4______	9______	1_____	2_____	3_____	4_____	9_____
		5______	6______	7______	8______	_______	5_____	6_____	7_____	8_____	
2		1______	2______	3______	4______	9______	1_____	2_____	3_____	4_____	9_____
		5______	6______	7______	8______	_______	5_____	6_____	7_____	8_____	
3		1______	2______	3______	4______	9______	1_____	2_____	3_____	4_____	9_____
		5______	6______	7______	8______	_______	5_____	6_____	7_____	8_____	
4		1______	2______	3______	4______	9______	1_____	2_____	3_____	4_____	9_____
		5______	6______	7______	8______	_______	5_____	6_____	7_____	8_____	
6		1______	2______	3______	4______	9______	1_____	2_____	3_____	4_____	9_____
		5______	6______	7______	8______	_______	5_____	6_____	7_____	8_____	
7		1______	2______	3______	4______	9______	1_____	2_____	3_____	4_____	9_____
		5______	6______	7______	8______	_______	5_____	6_____	7_____	8_____	
8		1______	2______	3______	4______	9______	1_____	2_____	3_____	4_____	9_____
		5______	6______	7______	8______	_______	5_____	6_____	7_____	8_____	
31		1______	2______	3______	4______	9______	1_____	2_____	3_____	4_____	9_____
		5______	6______	7______	8______	_______	5_____	6_____	7_____	8_____	
32		1______	2______	3______	4______	9______	1_____	2_____	3_____	4_____	9_____
		5______	6______	7______	8______	_______	5_____	6_____	7_____	8_____	

F2.1 Codes for Long-term Measures

1 Build resilent housing units
2 Invest in stronger public facilities
3 Build (Cement) dams, dikes and river embankments
4 Upgrade power and water lines
5 Major road repairs
6 Identify relocation areas
7 Rezoning and land-use regulations
8 Build drainage
9 Others, specify

F2.3 Codes for Length of Implementation

1 Less than 1 year before [SHOCK]
2 1 to 2 years before [SHOCK]
3 2 to 3 years before [SHOCK]
4 More than 3 years before [SHOCK]
5 After the [SHOCK]

Ravago, Mapa, Sunglao, Aycardo, (2020). Mendeley Data, doi: 10.17632/9y2yhw45zt.1

F	HARM MITIGATION (EX-ANTE REDUCTION OF EXPOSURE)											
Shock Code	F3						F4					
	**F3.1** Which of the following mid-term precautionary measures? ***Alin sa mga sumusunod na mid-term precautionary measures?*** **F3.2** Please rank these measures according to importance to recovery [1-Most important,2-Next most important, and so on.] ***Paki-rank ang mga measures base sa kung gaano sila ka-importante sa recovery [1-Most important, 2-Next most important, and so on.]***			**F3.3** How often are the measures implemented? ***Gaano kadalas pinapatupad ang mga measures?***			**F4.1** Which of the following short-term precautionary measures? ***Alin sa mga sumusunod na short-term precautionary measures?*** **F4.2** Please rank these measures according to importance to recovery [1-Most important, 2-Next most important, and so on.] ***Paki-rank ang mga measures base sa kung gaano sila ka-importante sa recovery [1-Most important, 2-Next most important, and so on.]***		**F4.3** When were the measures implemented? ***Kailan pinatupad ang mga measures?*** (Copy encircled codes in F4.2. For answers to F4.3, see code F4.3)		**F4.4** How long were the measures implemented? ***Gaano katagal pinatupad ang mga measures?*** (See code F4.4)	
	(For F3.1, encircle all answers that apply in below answer grid. For F3.2, Write the rank on the space provided beside the code.)			*(Copy encircled codes in F3.2. For answers to F3.3, see code F3.3)*			*(For F4.1, encircle all answers that apply in below answer grid. For F4.2, Write the rank on the space provided beside the code.)*					
1	1______	2______	3______	1_____	2_____	3_____	1______	2______	1_____	2_____	1_____	2_____
	4______	5______	6______ ______	4_____	5_____	6_____	3______	4______ ______	3_____	4_____	3_____	4_____
2	1______	2______	3______	1_____	2_____	3_____	1______	2______	1_____	2_____	1_____	2_____
	4______	5______	6______ ______	4_____	5_____	6_____	3______	4______ ______	3_____	4_____	3_____	4_____
3	1______	2______	3______	1_____	2_____	3_____	1______	2______	1_____	2_____	1_____	2_____
	4______	5______	6______ ______	4_____	5_____	6_____	3______	4______ ______	3_____	4_____	3_____	4_____
4	1______	2______	3______	1_____	2_____	3_____	1______	2______	1_____	2_____	1_____	2_____
	4______	5______	6______ ______	4_____	5_____	6_____	3______	4______ ______	3_____	4_____	3_____	4_____
6	1______	2______	3______	1_____	2_____	3_____	1______	2______	1_____	2_____	1_____	2_____
	4______	5______	6______ ______	4_____	5_____	6_____	3______	4______ ______	3_____	4_____	3_____	4_____
7	1______	2______	3______	1_____	2_____	3_____	1______	2______	1_____	2_____	1_____	2_____
	4______	5______	6______ ______	4_____	5_____	6_____	3______	4______ ______	3_____	4_____	3_____	4_____
8	1______	2______	3______	1_____	2_____	3_____	1______	2______	1_____	2_____	1_____	2_____
	4______	5______	6______ ______	4_____	5_____	6_____	3______	4______ ______	3_____	4_____	3_____	4_____
31	1______	2______	3______	1_____	2_____	3_____	1______	2______	1_____	2_____	1_____	2_____
	4______	5______	6______ ______	4_____	5_____	6_____	3______	4______ ______	3_____	4_____	3_____	4_____
32	1______	2______	3______	1_____	2_____	3_____	1______	2______	1_____	2_____	1_____	2_____
	4______	5______	6______ ______	4_____	5_____	6_____	3______	4______ ______	3_____	4_____	3_____	4_____

F3.1 Codes for Mid-term Measures
1 Assess safety of public buildings
2 Strengthen river embankments and dikes using sandbags
3 Clean sewers and canals
4 Conduct road assessment and repairs
5 Repair/rehabilitate classrooms
6 Others, specify

F3.3 Frequency of implementation
1 1 to 2 times since 2009/ 2012
2 3 to 5 times since 2009/ 2012
3 1 to 2 times a year
4 3 to 5 times a year
5 6 times or more a year

F4.1 Short-term Measures
1 Class suspension
2 Gale warning
3 Road closures
4 Others, specify

F4.3 Time of Implementation
1 More than 24 hours before [SHOCK]
2 24 hours or less before [SHOCK]
3 During [SHOCK]
4 Less than 24 hours after [SHOCK]
5 More than 24 hours after [SHOCK]

F4.4 Length of Implementation
1 Less than 1 day
2 1 to 3 days
3 4 days to 1 week
4 More than 1 week to 1 month
5 More than 1 month

Ravago, Mapa, Sunglao, Aycardo, (2020). Mendeley Data, doi: 10.17632/9y2yhw45zt.1

G	PREPAREDNESS (EARLY WARNING AND RESPONSE)							
	G1			G2		G3		
	Did your city/municipality receive or hear a warning before [SHOCK] occurred?<br>***Nakatanggap ba ng babala ang inyong siyudad/munisipyo bago nangyari ang [SHOCK]?***			After receiving or hearing the warning, did your city/municipality conduct any checks?<br>***Pagkatapos makatanggap ng babala, nagsagawa ba ng anumang checks ang inyong siyudad/munisipyo?***		Did your city/municipality issue a warning to your constituents?<br>***Nag-issue ba ang inyong siyudad/munisipyo ng babala sa inyong constituents?***		
	**G1.1**	**G1.2**	**G1.3**	**G2.1**	**G2.2**	**G3.1**	**G3.2**	**G3.3**
							(If 1 on G3.1)	
Shock Code	1 Yes<br>2 No ►G3	Who gave the warning?<br>***Kanino nangggaling ang babala?***<br>*(See code G1.2)*	Classify the warning.<br>***Paki-classify ang babala.***<br>*(See code G1.3)*	1 Yes<br>2 No ►G3	Which of the following preparatory checks?<br>***Alin sa mga sumusunod na mga preparatory checks?***<br>*(See code G2.2. Encircle all that apply)*	1 Yes<br>2 No ►H1	When?<br>***Kelan nag-issue?***<br>*(See code G3.2)*	Via what medium?<br>***Sa anong paraan na-issue?***<br>*(See code G3.3. Encircle all that apply.)*
1		1 2 3 4 5_________ 6_________ 7_________	1 2 3 4		1 2 3<br>4 5 6 7___________			1 2 3 4 5___________
2		1 2 3 4 5_________ 6_________ 7_________	1 2 3 4		1 2 3<br>4 5 6 7___________			1 2 3 4 5___________
3		1 2 3 4 5_________ 6_________ 7_________	1 2 3 4		1 2 3<br>4 5 6 7___________			1 2 3 4 5___________
4		1 2 3 4 5_________ 6_________ 7_________	1 2 3 4		1 2 3<br>4 5 6 7___________			1 2 3 4 5___________
6		1 2 3 4 5_________ 6_________ 7_________	1 2 3 4		1 2 3<br>4 5 6 7___________			1 2 3 4 5___________
7		1 2 3 4 5_________ 6_________ 7_________	1 2 3 4		1 2 3<br>4 5 6 7___________			1 2 3 4 5___________
8		1 2 3 4 5_________ 6_________ 7_________	1 2 3 4		1 2 3<br>4 5 6 7___________			1 2 3 4 5___________
31		1 2 3 4 5_________ 6_________ 7_________	1 2 3 4		1 2 3<br>4 5 6 7___________			1 2 3 4 5___________
32		1 2 3 4 5_________ 6_________ 7_________	1 2 3 4		1 2 3<br>4 5 6 7___________			1 2 3 4 5___________

G1.2 Codes for Warning
- 1 PAGASA/DOST
- 2 Provincial DRRMO
- 3 NDRRMC
- 4 Local media
- 5 Other government agency, specify
- 6 Other NGO, specify
- 7 Others, specify

G1.3 Codes for Types of Warning
- 1 Alpha - low risk or 51 to 100 km away from Charlie
- 2 Bravo - medium risk or 1 to 50 km away from Charlie
- 3 Charlie - high risk or critical area
- 4 Others, specify

G2.2 Codes for Preparatory Checks
- 1 Check inventory of supplies and equipment
- 2 Check capacity of critical facilities like hospitals
- 3 Organize DRRM teams and personnel
- 4 Enlist volunteers
- 5 Prepare evacuation centers
- 6 Prepare and preposition relief goods
- 7 Others, specify

G3.2 Codes for Warning
- 1 More than 24 hours before [SHOCK]
- 2 24 hours or less before [SHOCK]
- 3 During [SHOCK]
- 4 Less than 24 hours after [SHOCK]
- 5 More than 24 hours after [SHOCK]

G3.3 Codes for Medium
- 1 Television
- 2 Radio
- 3 SMS / Calls
- 4 Social media (e.g. Facebook, Twitter, etc.)
- 5 Others, specify

Ravago, Mapa, Sunglao, Aycardo, (2020). Mendeley Data, doi: 10.17632/9y2yhw45zt.1

NOTE: This section shall be pre-accomplished by the respondent. Responses in the sheet should be attached and verified by the enumerator before before turnover of all fieldwork materials for quality check.

H	RESPONSE (EX-POST LOSS REDUCTION): EVACUATION													
	H1	H2				H3						H4	H5	H6
	Did you issue a warning for evacuation? *Nag-issue ba kayo ng evacuation warning?* 1 Yes 2 No ►H2	Did you issue an order for evacuation? *Nag-issue ba kayo ng order para sa evacuation?*				Was there an evacuation center designated for the [SHOCK]? *Mayroon bang evacuation center na naka-assign para sa [SHOCK]?*						What percentage of the affected population complied with the evacuation order? *Ilang porsyento ng apektadong populasyon ang sumunod sa evacuation order?*	Did you conduct a forcible evacuation? *Nagkaroon ba kayo ng sapilitang evacuation?* 1 Yes 2 No	How long was the evacuation center used? *Gaano katagal ginamit ang evacuation center?* (See code H6)
		H2.1	H2.2		H2.3	H3.1	H3.2	H3.3		H3.4	H3.5			
Shock Code		1 Yes 2 No ►H3	*If respondent is Congressman:* How many towns were covered by the order? *Ilang mga bayan ang sakop ng order?*	*If respondent is Mayor:* How many barangays were covered by the order? *Ilang mga barangay ang sakop ng order?*	When was the order issued? *Kelan na-issue ang order?* (See code H2.3.)	1 Yes 2 No ►H4	How many? *Ilan ang mga evacuation centers na ito?*	How many families can the smallest and largest evacuation centers accommodate? *Ilang pamilya ang kasya pinakamaliit at pinakamalaking evacuation center?* Smallest *Pinakamaliit*	Biggest *Pinakamalaki*	What is/are their original use? *Ano ang orihinal nilang gamit* (See code H3.4. Encircle all that apply.)	Which of the following facilities are available? *Alin sa mga sumusunod na pasilidad ang meron?* (See code H3.5. Encircle all that apply.)			
1										1 2 3 4 5 6 ________	1 2 3 4 5 6___________			
2										1 2 3 4 5 6 ________	1 2 3 4 5 6___________			
3										1 2 3 4 5 6 ________	1 2 3 4 5 6___________			
4										1 2 3 4 5 6 ________	1 2 3 4 5 6___________			
6										1 2 3 4 5 6 ________	1 2 3 4 5 6___________			
7										1 2 3 4 5 6 ________	1 2 3 4 5 6___________			
8										1 2 3 4 5 6 ________	1 2 3 4 5 6___________			
31										1 2 3 4 5 6 ________	1 2 3 4 5 6___________			
32										1 2 3 4 5 6 ________	1 2 3 4 5 6___________			

H2.3 Codes for Time of Implementation
1 More than 24 hours before [SHOCK]
2 24 hours or less before [SHOCK]
3 During [SHOCK]
4 Less than 24 hours after [SHOCK]
5 More than 24 hours after [SHOCK]

H3.4 Codes for Original Use
1 Public school building
2 Public gym/basketball court, etc.
3 Municipal hall
4 Church
5 Private building
6 Others, specify

H3.5 Codes for Facilities
1 Toilets
2 Generators
3 Common Kitchen
4 Health Station
5 Assembly Area
6 Others, specify

H6 Codes for Center Use
1 Less than 1 day
2 1 to 3 days
3 4 days to 1 week
4 More than 1 week to 1 month
5 More than 1 month to 6 months
6 More than 6 months to 1 year
7 More than 1 year

Ravago, Mapa, Sunglao, Aycardo, (2020). Mendeley Data, doi: 10.17632/9y2yhw45zt.1

I	RESPONSE (EX-POST LOSS REDUCTION): SEARCH AND RESCUE												
Shock Code	I1		I2							I3			
	Did your city/municipality conduct search and rescue?<br>***Nagsagawa ba ng search and rescue ang inyong siyudad/munisipyo?***		Did the [SHOCK] result in death, illness, injury of your constituents?<br>***Meron bang namatay, nagkasakit, o nasugatan sa inyong siyudad/munisipyo ng dahil sa [SHOCK]?***							While doing the search and rescue when [SHOCK] occured, have any LGU employee involved in DRRM died, got injuried, or got sick?<br>***Noong nagsasagawa ng search and rescue para sa [SHOCK], may mga empleyado ba ng LGU na parte ng DRRM na namatay, nasugatan, o nagkasakit?***			
	**I1.1**	**I1.2**	**I2.1**	**I2.2**			**I2.3**			**I3.1**	**I3.2**		
	1 Yes<br>2 No ►I2	How many were rescued?<br>***Ilan ang na-rescue?***<br>*(See code I1.2)*	1 Yes<br>2 No ►I3	*(See code I2.2. Encircle all that apply)*			How many were affected?<br>***Gaano karami ang ...?***<br>*(See code I2.3)*			1 Yes<br>2 No ►J1	How many...?<br>***Ilan ang...?***		
											Died<br>***Namatay***	Got injured<br>***Nasugatan***	Got sick<br>***Nagkasakit***
1				1	2	3	1_______	2_______	3_______				
2				1	2	3	1_______	2_______	3_______				
3				1	2	3	1_______	2_______	3_______				
4				1	2	3	1_______	2_______	3_______				
6				1	2	3	1_______	2_______	3_______				
7				1	2	3	1_______	2_______	3_______				
8				1	2	3	1_______	2_______	3_______				
31				1	2	3	1_______	2_______	3_______				
32				1	2	3	1_______	2_______	3_______				

I1.2 Codes for People Rescued

1 Less than 100 people
2 101 to 200 people
3 201 to 300 people
4 301 to 400 people
5 401 to 500 people
6 More than 501 people

I2.2 Codes for Effects

1 Death
2 Illness
3 Injury

Ravago, Mapa, Sunglao, Aycardo, (2020). Mendeley Data, doi: 10.17632/9y2yhw45zt.1

NOTE: This section shall be pre-accomplished by the respondent. Responses in the sheet should be attached and verified by the enumerator before turnover of all fieldwork materials for quality check.

J	RESPONSE (EX-POST LOSS REDUCTION): STATE OF CALAMITY					
	J1	J2				
	Did your city/municipality declare a State of Calamity?<br>***Nagdeklara ba ng State of Calamity ang inyong siyudad/munisipyo?***<br>1 Yes 2 No ►J3	Did you use your Quick Response Fund?<br>***Ginamit ba ninyo ang inyong Quick Response Fund?***				
Shock Code		J2.1<br>1 Yes<br>2 No<br>►J3	J2.2<br>How much did you use?<br>***Magkano ang inyong ginamit?***	J2.3 How did you use your fund?<br>***Paano mo ginamit ang pondo?***<br>*(See code J2.3. Encircle all that apply.)*<br>J2.4 How much did you use for (MENTION ANSWER IN J2.3)<br>***Magkano ang ginamit ninyo sa (MENTION ANSWER IN J2.3)***	J2.5<br>*(If 11 in J2.3)*<br>What was the monetary assistance for?<br>***Para saan ang monetary assistance?***<br>*(See code J2.5. Encircle all that apply.)*	J2.6<br>*(If 11 in J2.3)*<br>How much was given, on average, to each household?<br>***Magkano ang binigay ninyo sa bawat pamilya?***
1				1____ 2____ 3____ 4____ 5____ 6____ 12____ ____<br>7____ 8____ 9____ 10____ 11____	1 2 3 4 5____	
2				1____ 2____ 3____ 4____ 5____ 6____ 12____ ____<br>7____ 8____ 9____ 10____ 11____	1 2 3 4 5____	
3				1____ 2____ 3____ 4____ 5____ 6____ 12____ ____<br>7____ 8____ 9____ 10____ 11____	1 2 3 4 5____	
4				1____ 2____ 3____ 4____ 5____ 6____ 12____ ____<br>7____ 8____ 9____ 10____ 11____	1 2 3 4 5____	
6				1____ 2____ 3____ 4____ 5____ 6____ 12____ ____<br>7____ 8____ 9____ 10____ 11____	1 2 3 4 5____	
7				1____ 2____ 3____ 4____ 5____ 6____ 12____ ____<br>7____ 8____ 9____ 10____ 11____	1 2 3 4 5____	
8				1____ 2____ 3____ 4____ 5____ 6____ 12____ ____<br>7____ 8____ 9____ 10____ 11____	1 2 3 4 5____	
31				1____ 2____ 3____ 4____ 5____ 6____ 12____ ____<br>7____ 8____ 9____ 10____ 11____	1 2 3 4 5____	
32				1____ 2____ 3____ 4____ 5____ 6____ 12____ ____<br>7____ 8____ 9____ 10____ 11____	1 2 3 4 5____	

J2.3 Codes for Fund Use

1 Search and Rescue
2 Relief Goods Procurement
3 Soup Kitchen
4 Other Relief Operations
5 Clean-up Operations
6 Restoration of Lifeline Services
7 Employment and Livelihood
8 Housing and Relocation
9 Reconstruction of damaged buildings
10 Replacement and Repair of Lost Assets
11 Monetary Assistance
12 Others, specify

J2.5 Codes for Monetary Assistance

1 Emergency Shelter
2 Livelihood
3 Health
4 Unconditional
5 Others, specify

Ravago, Mapa, Sunglao, Aycardo, (2020). Mendeley Data, doi: 10.17632/9y2yhw45zt.1

NOTE: This section shall be pre-accomplished by the respondent. Responses in the sheet should be attached and verified by the enumerator before turnover of all fieldwork materials for quality check.

J	RESPONSE (EX-POST LOSS REDUCTION): STATE OF CALAMITY											
	J3						J4				J5	
	Did you avail of funding from the National Disaster Fund/Calamity Fund?<br>***Kumuha ba kayo ng pondo mula sa National Disaster Fund/Calamity Fund?***						**J4.1**	**J4.2**	**J4.3**	**J4.4**	**J5.1**	**J5.2**
	**J3.1**	**J3.2**	**J3.3**	**J3.4**	**J3.5**	**J3.6**		*(If code 1 in J4.1)*				
Shock Code	1 Yes<br>2 No<br>►J4	How much did you get?<br>***Magkano ang inyong kinuha?***	Which of the following agencies released the funding?<br>***Alin sa mga sumusunod na ahensya ang naglabas ng pondo?***<br>(See code J3.3. Encircle all that apply.)	How did you use your fund?<br>***Paano mo ginamit ang pondo?***<br>(See code J3.4. Encircle all that apply.)	*(If 11 in J3.4)*<br>What was the monetary assistance for?<br>***Para saan ang monetary assistance?***<br>(See code J3.5. Encircle all that apply.)	*(If 11 in J3.4)*<br>How much was given, on average, to each household?<br>***Magkano ang binigay ninyo sa bawat pamilya?***	Does your city/municipality have insurance against [SHOCK]?<br>***Meron bang insurance para sa [SHOCK] ang inyong siyudad/munisipyo?***<br>1 Yes<br>2 No ►J5	How much did you get?<br>***Magkano ang nakuha ninyo?***	Who provided the insurance?<br>***Kanino galing ang insurance?***	How did you use the fund?<br>***Saan ninyo ginamit ang pondo?***	Did your city/municipality issue a price freeze/ceiling?<br>***Nag-issue ba ang inyong siyudad/munisipyo ng price freeze/ceiling?***<br>1 Yes<br>2 No ►K1	For how long?<br>***Gaano katagal?***<br>(See code J5.2)
1			1 2 3 4 5<br>6 7 8 9 10<br>11_________	1 2 3 4 5 6<br>7 8 9 10 11<br>12_________	1 2 3 4 5__________							
2			1 2 3 4 5<br>6 7 8 9 10<br>11_________	1 2 3 4 5 6<br>7 8 9 10 11<br>12_________	1 2 3 4 5__________							
3			1 2 3 4 5<br>6 7 8 9 10<br>11_________	1 2 3 4 5 6<br>7 8 9 10 11<br>12_________	1 2 3 4 5__________							
4			1 2 3 4 5<br>6 7 8 9 10<br>11_________	1 2 3 4 5 6<br>7 8 9 10 11<br>12_________	1 2 3 4 5__________							
6			1 2 3 4 5<br>6 7 8 9 10<br>11_________	1 2 3 4 5 6<br>7 8 9 10 11<br>12_________	1 2 3 4 5__________							
7			1 2 3 4 5<br>6 7 8 9 10<br>11_________	1 2 3 4 5 6<br>7 8 9 10 11<br>12_________	1 2 3 4 5__________							
8			1 2 3 4 5<br>6 7 8 9 10<br>11_________	1 2 3 4 5 6<br>7 8 9 10 11<br>12_________	1 2 3 4 5__________							
31			1 2 3 4 5<br>6 7 8 9 10<br>11_________	1 2 3 4 5 6<br>7 8 9 10 11<br>12_________	1 2 3 4 5__________							
32			1 2 3 4 5<br>6 7 8 9 10<br>11_________	1 2 3 4 5 6<br>7 8 9 10 11<br>12_________	1 2 3 4 5__________							

J3.3 Codes for Agency
- 1 DSWD
- 2 DILG
- 3 DOH
- 4 DepEd
- 5 DA
- 6 DPWH
- 7 AFP-OCD
- 8 BFP
- 9 Coast Guard
- 10 PNP
- 11 Others, specify

J3.4 Codes for Fund Use
- 1 Search and Rescue
- 2 Relief Goods Procurement
- 3 Soup Kitchen
- 4 Other Relief Operations
- 5 Clean-up Operations
- 6 Restoration of Lifeline Services
- 7 Employment and Livelihood
- 8 Housing & Relocation
- 9 Reconstruction of damaged buildings
- 10 Replacement & Repair of Lost Assets
- 11 Monetary Assistance
- 12 Others, specify

J3.5 Codes for Monetary
- 1 Emergency Shelter
- 2 Livelihood
- 3 Health
- 4 Unconditional
- 5 Others, specify

J5.2 Codes for Price Freeze/Ceiling
- 1 Less than 1 day
- 2 1 to 3 days
- 3 4 days to 1 week
- 4 More than 1 week to 1 month
- 5 More than 1 month to 6 months
- 6 More than 6 months to 1 year
- 7 More than 1 year

Ravago, Mapa, Sunglao, Aycardo, (2020). Mendeley Data, doi: 10.17632/9y2yhw45zt.1

K	RECOVERY (COPING) : RELIEF														
	K1														
	Did your city/municipality extend assistance to their constituents after [SHOCK] occurred?<br>***Nagbigay ba ng tulong ang inyong siyudad/munisipyo sa mga constituents pagkatapos ng [SHOCK]?***														
	**K1.1**	**K1.2**	**K1.3**					**K1.4**					**K1.5**	**K1.6**	**K1.7**
Shock Code	1 Yes<br>2 No<br>►L1	Was the assistance…<br>***Ang tulong ba ay…***<br>1 Adequate<br>***Sapat***<br>2 Inadequate<br>***Hindi sapat***	What types of relief?<br>***Anong klase ng relief?***<br>*(See code K1.3. Encircle all that apply.)*					From the time [SHOCK] occurred, when was [ANSWER IN J1.3] provided?<br>***Mula nang mangyari ang [SHOCK], kailan naibigay ang [ANSWER IN J1.3]?***<br>*(See code K1.4)*					How many families have you given relief to?<br>***Ilang mga pamilya ang binigyan ninyo ng relief?***	*(If answered code 3 in K1.3)* How many days did food and water relief last?<br>***Ilang araw tumagal ang ipinamahaging pagkain at tubig?***	*(If answered code 3 in K1.3)* Was there enough relief goods for everyone?<br>***Sapat ba ang inyong supply ng mga relief goods para sa lahat?***<br>1 Yes<br>2 No
1			1	2	3	4	5___________	1_____	2_____	3_____	4_____	5_____			
2			1	2	3	4	5___________	1_____	2_____	3_____	4_____	5_____			
3			1	2	3	4	5___________	1_____	2_____	3_____	4_____	5_____			
4			1	2	3	4	5___________	1_____	2_____	3_____	4_____	5_____			
6			1	2	3	4	5___________	1_____	2_____	3_____	4_____	5_____			
7			1	2	3	4	5___________	1_____	2_____	3_____	4_____	5_____			
8			1	2	3	4	5___________	1_____	2_____	3_____	4_____	5_____			
31			1	2	3	4	5___________	1_____	2_____	3_____	4_____	5_____			
32			1	2	3	4	5___________	1_____	2_____	3_____	4_____	5_____			

K1.3 Codes for Types of Assistance
1 Soup kitchen
2 Emergency shelter kit
3 Relief goods (e.g. food pack and water)
4 Medical kit
5 Others, specify

K1.4 Codes for Start of Relief
1 During [SHOCK]
2 Less than 1 day after [SHOCK]
3 1 to 3 days after [SHOCK]
4 4 days to 1 week after [SHOCK]
5 More than 1 week to 1 month after [SHOCK]
6 More than 1 month after [SHOCK]

K1.6 Codes for Duration of Relief
1 Less than 1 day
2 1 to 3 days
3 4 days to 1 week
4 More than 1 week to 1 month
5 More than 1 month to 6 months
6 More than 6 months to 1 year
7 More than 1 year

Ravago, Mapa, Sunglao, Aycardo, (2020). Mendeley Data, doi: 10.17632/9y2yhw45zt.1

L	RECOVERY (COPING) : CLEAN-UP OPERATIONS			
	L1			
	L1.1	L1.2	L1.3	L1.4
Shock Code	Did your city/municipality undertake clean-up operations?<br>***May ginawa bang clean-up operations ang inyong siyudad/munisipyo?***<br>1 Yes<br>2 No ►M1	When did it start?<br>***Kelan nag-umpisa?***<br>*(See code L1.2)*	How long did it take?<br>***Gaano katagal?***<br>*(See code L1.3)*	How much was the clean-up operations cost?<br>***Magkano ang nagastos sa clean-up operations?***<br>*In pesos*
1				
2				
3				
4				
6				
7				
8				
31				
32				

M	RECOVERY (COPING): EMPLOYMENT					
	M1			M2		
	Was there a cash-for-work for the [SHOCK]?<br>***Nagkaroon ba ng cash-for-work program para sa [SHOCK]?***			Was there a food-for-work program for the [SHOCK]?<br>***Nagkaroon ba ng food-for-work program para sa [SHOCK]?***		
	M1.1	M1.2	M1.3	M2.1	M2.2	M2.3
Shock Code	1 Yes<br>2 No<br>►M2	How much is the daily rate? *(In pesos)*<br>***Magkano kada araw?***<br>***(In pesos)***	How many people did you hire?<br>***Ilang mga tao ang na-hire?***	1 Yes<br>2 No<br>►N1	How much do you think is the value of the food for a day's work?<br>***Magkano sa palagay mo ang halaga ng pagkain para sa isang araw na trabaho?***	How many people did you hire?<br>***Ilang mga tao ang na-hire?***
1						
2						
3						
4						
6						
7						
8						
31						
32						

L1.2 Codes for Start of Cleanup
1 During [SHOCK]
2 Less than 1 day after [SHOCK]
3 1-3 days after [SHOCK]
4 4 days-1 week after [SHOCK]
5 More than 1 week - 1 month after [SHOCK]
6 More than 1 month after [SHOCK]

L1.3 Codes for Duration of Clean-up
1 Less than 1 day
2 1 to 3 days
3 4 days to 1 week
4 More than 1 week to 1 month
5 More than 1 month to 6 months
6 More than 6 months to 1 year
7 More than 1 year

M1.2 Codes for Daily Rate
1 Less than Php 150
2 Php 150-300
3 Php 301-450
4 Php 451-600
5 More than Php 600

M2.2 Codes for Value of Food
1 Less than Php 150
2 Php 150-300
3 Php 301-450
4 Php 451-600
5 More than Php 600

Ravago, Mapa, Sunglao, Aycardo, (2020). Mendeley Data, doi: 10.17632/9y2yhw45zt.1

N	RECOVERY (COPING): RESPONSE FROM OTHERS																
	N1																
	Did other government agencies, LGUs or NGOs extend assistance during and/or after [SHOCK]?<br>***May mga iba bang government agencies, LGUs o NGOs na nagbigay ng tulong habang or pagkatapos ng [SHOCK]?***																
	**N1.1**	**N1.2**								**N1.3**	**N1.4**						**N1.5**
Shock Code	1 Yes<br>2 No ►O1	Which of the following agencies or other LGUs extended assistance?<br>***Alin sa mga sumusunod na mga ahensiya o ibang LGU ang nabigay ng tulong?***<br>*(See code N1.2. Encircle all that apply.)*								*(If 12 or 13 in N1.2)*<br>Is the LGU a sister city/municipality?<br>***Ang LGU ba ay sister city/municipality?***<br>1-Yes 2-No	Which of the following types of assistance were provided?<br>***Alin sa mga sumusunod na klase ng tulong ang binigay?***<br>*(See code N1.4. Encircle all that apply.)*						When was the assistance provided?<br>***Kailan naibigay ang tulong?***<br>*(See code N1.5)*
1		1	2	3	4	5	6	7	8		1	2	3	4	5	6	12_________	
		9	10	11_______	12_______	13_______		14	15		7	8	9	10	11			
2		1	2	3	4	5	6	7	8		1	2	3	4	5	6	12_________	
		9	10	11_______	12_______	13_______		14	15		7	8	9	10	11			
3		1	2	3	4	5	6	7	8		1	2	3	4	5	6	12_________	
		9	10	11_______	12_______	13_______		14	15		7	8	9	10	11			
4		1	2	3	4	5	6	7	8		1	2	3	4	5	6	12_________	
		9	10	11_______	12_______	13_______		14	15		7	8	9	10	11			
6		1	2	3	4	5	6	7	8		1	2	3	4	5	6	12_________	
		9	10	11_______	12_______	13_______		14	15		7	8	9	10	11			
7		1	2	3	4	5	6	7	8		1	2	3	4	5	6	12_________	
		9	10	11_______	12_______	13_______		14	15		7	8	9	10	11			
8		1	2	3	4	5	6	7	8		1	2	3	4	5	6	12_________	
		9	10	11_______	12_______	13_______		14	15		7	8	9	10	11			
31		1	2	3	4	5	6	7	8		1	2	3	4	5	6	12_________	
		9	10	11_______	12_______	13_______		14	15		7	8	9	10	11			
32		1	2	3	4	5	6	7	8		1	2	3	4	5	6	12_________	
		9	10	11_______	12_______	13_______		14	15		7	8	9	10	11			

N1.2 Codes for Agency

1 DSWD
2 DILG
3 DOH
4 DepEd
5 DA
6 DPWH
7 AFP-OCD
8 BFP
9 Coast Guard
10 PNP
11 Other agency
12 Other city
13 Other province
14 Local NGOs
15 Foreign NGOs

N1.4 Codes for Other response

1 Search and Rescue
2 Relief Goods
3 Soup Kitchen
4 Other Relief Operations
5 Clean-up Operations
6 Restoration of Lifeline Services
7 Employment and Livelihood
8 Housing and Relocation
9 Reconstruction of damaged buildings
10 Replacement and Repair of Lost Assets
11 Monetary Assistance
12 Others, specify

N1.5 Codes for Length

1 Less than 1 day
2 1 to 3 days
3 4 days to 1 week
4 More than 1 week to 1 month
5 More than 1 month to 6 months
6 More than 6 months to 1 year
7 More than 1 year

Ravago, Mapa, Sunglao, Aycardo, (2020). Mendeley Data, doi: 10.17632/9y2yhw45zt.1

O	RECOVERY (COPING): LOANS																	
	**O1**<br>Did your city/municipality ask for a loan for [SHOCK]??<br>***Umutang ba ang inyong siyudad o munisipyo para sa [SHOCK]?***																	
	**O1.1**	**O1.2**	**O1.3**	**O1.4**						**O1.5**						**O1.6**	**O1.7**	**O1.8**
Shock Code	1 Yes<br>2 No ►Q1	Did you get the loan?<br>***Nakuha ba ninyo ang pautang?***<br>1 Yes<br>2 No ►O1.4	When was loan released?<br>***Kailan na-release ang pautang?***<br>*(See code O1.3)*	Where did you borrow the loans from?<br>***Kanino kayo umutang?***<br>*(See code O1.4. Encircle all that apply)*						How much was the loan?<br>***Magkano ang halaga ng utang?***<br>*(Estimate in pesos.)*						How did you use your loan?<br>***Paano mo ginamit ang utang?***<br>*(See code O1.6. Encircle all that apply.)*	How much was the interest?<br>***Magkano ang interes?***	How long was the payment term?<br>***Ilang taon ang pagbayad?***
1				1	2	3	4	5	6	1________	2________	3________	4________	5________	6________	1 2 3 4 5 6<br>7 8 9 10 11<br>12__________		
2				1	2	3	4	5	6	1________	2________	3________	4________	5________	6________	1 2 3 4 5 6<br>7 8 9 10 11<br>12__________		
3				1	2	3	4	5	6	1________	2________	3________	4________	5________	6________	1 2 3 4 5 6<br>7 8 9 10 11<br>12__________		
4				1	2	3	4	5	6	1________	2________	3________	4________	5________	6________	1 2 3 4 5 6<br>7 8 9 10 11<br>12__________		
6				1	2	3	4	5	6	1________	2________	3________	4________	5________	6________	1 2 3 4 5 6<br>7 8 9 10 11<br>12__________		
7				1	2	3	4	5	6	1________	2________	3________	4________	5________	6________	1 2 3 4 5 6<br>7 8 9 10 11<br>12__________		
8				1	2	3	4	5	6	1________	2________	3________	4________	5________	6________	1 2 3 4 5 6<br>7 8 9 10 11<br>12__________		
31				1	2	3	4	5	6	1________	2________	3________	4________	5________	6________	1 2 3 4 5 6<br>7 8 9 10 11<br>12__________		
32				1	2	3	4	5	6	1________	2________	3________	4________	5________	6________	1 2 3 4 5 6<br>7 8 9 10 11<br>12__________		

O1.3 Codes for Length
- 1 Less than 1 day
- 2 1 to 3 days
- 3 4 days to 1 week
- 4 More than 1 week to 1 month
- 5 More than 1 month to 6 months
- 6 More than 6 months to 1 year
- 7 More than 1 year

O1.4 Codes for Borrowing
- 1 Landbank/DBP
- 2 Commercial bank (BPI, BDO, PNB, UCPB, etc.)
- 3 National Government
- 4 Local/Foreign NGO
- 5 Foreign Development Agency
- 6 Foreign Government

O1.6 Codes for Fund Use
- 1 Search and Rescue
- 2 Relief Goods Procurement
- 3 Soup Kitchen
- 4 Other Relief Operations
- 5 Clean-up Operations
- 6 Restoration of Lifeline Services
- 7 Employment and Livelihood
- 8 Housing and Relocation
- 9 Reconstruction of damaged buildings
- 10 Replacement and Repair of Lost Assets
- 11 Monetary Assistance
- 12 Others, specify

Ravago, Mapa, Sunglao, Aycardo, (2020). Mendeley Data, doi: 10.17632/9y2yhw45zt.1

P	REHABILITATION (COPING): LIFELINE SERVICES																
Shock Code	P1				P2							P3					
	P1.1	P1.2			P2.1	P2.2	P2.3	P2.4				P3.1	P3.2	P3.3	P3.4		
					Water *(If 1 in P1.2)*							Telecommunication *(If 2 in P1.2)*					
	Were there any services that were interrupted during [SHOCK] ***Mayroon bang mga serbisyong naputol o natigil noong may [SHOCK]?*** 1 Yes 2 No ►P1	Which services were interrupted? ***Alin sa mga serbisyong ito ang naputol o natigil?*** *(See code P1.2. Encircle all that apply.)*			When was the water supply cut off? ***Kailan naputol ang supply ng tubig?*** *(See code P2.1)*	What % of the city/ municipality were affected? ***Ilang porsyento ng siyudad/munisipyo ang naapektuhan nito?***	How long was the interruption? ***Gaano katagal naputol ang supply ng tubig?*** *(See code P2.3)*	Which of the following stopgap measures were utilized? ***Alin sa mga stopgap measures ang inyong nagamit?*** *(See code P2.4)*				When was telecommunication interrupted? ***Kailan natigil ang serbisyong telecomunikasyon?*** *(See code P3.1)*	What % of the city/ municipality were affected? ***Ilang porsyento ng siyudad/munisipyo ang naapektuhan nito?***	How long was the interruption? ***Gaano katagal natigil ang telekomunikasyon?*** *(See code P3.3)*	Which of the following stopgap measures were utilized? ***Alin sa mga stopgap measures ang inyong nagamit?*** *(See code P3.4)*		
1		1	2	3				1	2	3	4________				1	2	3________
2		1	2	3				1	2	3	4________				1	2	3________
3		1	2	3				1	2	3	4________				1	2	3________
4		1	2	3				1	2	3	4________				1	2	3________
6		1	2	3				1	2	3	4________				1	2	3________
7		1	2	3				1	2	3	4________				1	2	3________
8		1	2	3				1	2	3	4________				1	2	3________
31		1	2	3				1	2	3	4________				1	2	3________
32		1	2	3				1	2	3	4________				1	2	3________

P1.2 Codes
for Services
1 Water
2 Telecommunication
3 Electricity

P2.1/3.1/4.1 Codes for Start of Interruption
1 More than 24 hours before [SHOCK]
2 24 hours or less before [SHOCK]
3 During [SHOCK]
4 Less than 24 hours after [SHOCK]
5 More than 24 hours after [SHOCK]

P2.3/3.3/4.3 Codes for Length of Interruption
1 Less than 1 day
2 1 to 3 days
3 4 days to 1 week
4 More than 1 week to 1 month
5 More than 1 month to 6 months
6 More than 6 months to 1 year
7 More than 1 year

P2.4 Water stopgap
1 Rationing
2 Water Wells
3 Mobile Water Treatment
4 Others, specify

P3.4 Telecomm. stopgap
1 Satellite phone
2 Two-way radio
3 Others, specify

Ravago, Mapa, Sunglao, Aycardo, (2020). Mendeley Data, doi: 10.17632/9y2yhw45zt.1

[SPIEL]: Now, I will ask about the general experience of your city/municipality on power interruptions, **aside** from those caused by shocks.
Ngayon, magtatanong ako tungkol sa mga power interruptions sa inyong siyudad/munisipyo, bukod sa mga dulot ng shocks.

P	REHABILITATION (COPING): LIFELINE SERVICES					
	P4					
	P4.1	P4.2	P4.3	P4.4		
	Electricity *(If 1 in P1.2)*					
Shock Code	When was the electricity cut off? ***Kailan naputol ang kuryente?*** *(See code P4.1)*	What % of the city/ municipality were affected? ***Ilang porsyento ng siyudad/munisipyo ang naapektuhan nito?***	How long was the interruption? ***Gaano katagal naputol ang kuryente?*** *(See code P4.3)*	Which of the following stopgap measures were utilized? ***Alin sa mga stopgap measures ang inyong nagamit?*** *(See code P4.4)*		
1				1	2	3________
2				1	2	3________
3				1	2	3________
4				1	2	3________
6				1	2	3________
7				1	2	3________
8				1	2	3________
31				1	2	3________
32				1	2	3________

REHABILITATION (COPING): LIFELINE SERVICES								
P5						P5.4		
P5.1	P5.2	What are the frequent causes of these power interruptions? ***Ano ang mga kadalasang sanhi ng mga power interruptions?*** *(Encircle all that apply.)*				How many hours is the… ***Ilang oras ang…***		
Every year, aside from those caused by shocks, how many times does your city/municipality experience power interruptions? ***Kada taon, bukod sa dulot ng shocks, ilang beses nagkakaroon ng power interruptions ang inyong siyudad/munisipyo?*** *(See code P5.1)* ***NOTE:*** *This is a general question, not to be asked per shock.*	P5.3	Please rank these causes according to frequency of causing interruptions *[1-Most frequent, 2-Next Most Frequent, and so on]* ***Paki-rank ang mga measures base sa kung gaano sila kadalas nagdudulot ng power interruptions [1-Most frequent, 2-Next Most Frequent, and so on]*** *(Answer grid for P5.2 and P5.3 below. Write rank on the space provided.* ***NOTE:*** *These are general questions, not to be asked per shock.)*				Shortest power interruption ***Pinakamaiksing power interruption***	Average power interruption ***Karaniwang power interruption***	Longest power interruption ***Pinakamahabang power interruption***
________________________	1 __________	2 __________	3 __________	4__________	__________	_______ hours	_______ hours	_______ hours

P4.1 Start of Interruption
1 More than 24 hours before [SHOCK]
2 24 hours or less before [SHOCK]
3 During [SHOCK]
4 Less than 3 hours after [SHOCK]
5 3 to 12 hours after [SHOCK]
6 13 hours to 24 hours after [SHOCK]
7 More than 24 hours after [SHOCK]

P4.3 Length of Interruption
1 Less than 1 day
2 1 to 3 days
3 4 days to 1 week
4 More than 1 week to 1 month
5 More than 1 month to 6 months
6 More than 6 months to 1 year
7 More than 1 year

P5.1 Stopgap Measures
1 Gas or Diesel-powered generators
2 Solar panels
3 Others, specify

P5.1 No. of Power Interruptions
1 Less than 5 times a year
2 6 to 10 times a year
3 11 to 15 times a year
4 16 to 20 times a year
5 More than 20 times a year

P5.2 Cause of Power Interruptions
1 Regular maintenance
2 Rotational brownouts
3 Damage to transmission lines or other electrical facilities
4 Others, specify

Ravago, Mapa, Sunglao, Aycardo, (2020). Mendeley Data, doi: 10.17632/9y2yhw45zt.1

NOTE: This section shall be pre-accomplished by the respondent. Responses in the sheet should be attached and verified by the enumerator before turnover of all fieldwork materials for quality check.

Q	RECONSTRUCTION (COPING)																					
	Q1																	Q2				
	Did any public infrastructure or facility break down during or after the [SHOCK]? *May nasira bang pampublikong pasilidad o kagamitan habang o pagkatapos ng [SHOCK]?*																	Did your office lose any files, records, or data during or after the [SHOCK]? ***Nawalan ba ng files, records, o data ba ang opisina ninyo habang o pagkatapos ng [SHOCK]?***				
	**Q1.1**	**Q1.2**						**Q1.3**	**Q1.4**	**Q1.5**	**Q1.6**							**Q2.1**	**Q2.2**	**Q2.3**		
Shock Code	1 Yes<br>2 No ►Q1	Which ones?<br>***Alin sa mga sumusunod?***<br>*(See code Q1.2. Encircle all that apply.)*						Was the damage fixed?<br>***Napaayos na ba ang sira?***<br>1 Yes<br>2 No	*(If yes on Q1.3)* Length of repair?<br>***Gaano katagal pinaayos?***<br>*(See code Q1.4)*	How much was spent?<br>***Magkano ang nagastos?***<br>*(In pesos)*	Which of the following funded the repair?<br>***Aling sa mga sumusunod ang nagbigay ng pondo para sa pagpapaayos?***<br>*(See code Q1.6)*							1 Yes<br>2 No ►Q2	Were you able to recover these?<br>***Narecover ba ninyo ang mga ito?***<br>1 - Yes 2 - No	What caused the loss of these?<br>***Anong dahilan ng pagkawala ng mga ito?***<br>*(See code Q2.3. Encircle all that apply.)*		
1		1	2	3	4	5	6				1	2	3	4	5	11			1	2	3	4__________
		7	8	9	10	11	12				6	7	8	9	10	12 _________						
2		1	2	3	4	5	6				1	2	3	4	5	11			1	2	3	4__________
		7	8	9	10	11	12				6	7	8	9	10	12 _________						
3		1	2	3	4	5	6				1	2	3	4	5	11			1	2	3	4__________
		7	8	9	10	11	12				6	7	8	9	10	12 _________						
4		1	2	3	4	5	6				1	2	3	4	5	11			1	2	3	4__________
		7	8	9	10	11	12				6	7	8	9	10	12 _________						
6		1	2	3	4	5	6				1	2	3	4	5	11			1	2	3	4__________
		7	8	9	10	11	12				6	7	8	9	10	12 _________						
7		1	2	3	4	5	6				1	2	3	4	5	11			1	2	3	4__________
		7	8	9	10	11	12				6	7	8	9	10	12 _________						
8		1	2	3	4	5	6				1	2	3	4	5	11			1	2	3	4__________
		7	8	9	10	11	12				6	7	8	9	10	12 _________						
31		1	2	3	4	5	6				1	2	3	4	5	11			1	2	3	4__________
		7	8	9	10	11	12				6	7	8	9	10	12 _________						
32		1	2	3	4	5	6				1	2	3	4	5	11			1	2	3	4__________
		7	8	9	10	11	12				6	7	8	9	10	12 _________						

Q1.2 Codes for Infrastructure

1 LDRRM office
2 Municipal hall
3 Health office, health center
4 Public school
5 Public gym
6 Other government-owned buildings
7 Public equipment
8 Public vehicles
9 Bridges, roads
10 Water facilities
11 Electrical facilities
12 Communication facilities (e.g. cell sites)

Q1.4 Codes for Length

1 Less than 1 day
2 1 to 3 days
3 4 days to 1 week
4 More than 1 week to 1 month
5 More than 1 month to 6 months
6 More than 6 months to 1 year
7 More than 1 year

Q1.6 Codes for Agency

1 Own city/municipality
2 DSWD
3 DILG
4 DOH
5 DepEd
6 DA
7 DPWH
8 AFP-OCD
9 BFP
10 Coast Guard
11 PNP
12 Others, specify

Q2.3 Codes for Cause

1 Fire
2 Flood
3 Damaged building structure
4 Others, specify

Ravago, Mapa, Sunglao, Aycardo, (2020). Mendeley Data, doi: 10.17632/9y2yhw45zt.1

NOTE: SECTION R shall be pre-accomplished by the respondent. Responses in the sheet should be attached and verified by the enumerator before turnover of all fieldwork materials for quality check.

NOTE: Ask SECTION S for all shock types.

R	RECONSTRUCTION (COPING): HOUSING AND RELOCATION					
	R1			R2		
	Did people move residences because of the [SHOCK]? ***May mga lumipat ba ng tirahan dahil sa [SHOCK]?***			Did your city/municipality create housing programs in response to the [SHOCK]? ***May nilikha bang mga housing program ang inyong siyudad/munisipyo nang dahil sa [SHOCK]?***		
	**R1.1**	**R1.2**	**R1.3**	**R2.1**	**R2.2**	**R2.4**
Shock Code	*(See code R1.1. Encircle all that apply. Go to S1 if code 3)*	*(If 1 in R1.1)* How many families moved into temporary shelters? ***Ilang pamilya ng lumipat sa mga temporary shelters?***	*(If 2 in R1.1)* How many families moved permanently? ***Ilang pamilya ng lumipat nang permanente?***	1 Yes<br>2 No ►S1	When did it start? ***Kailan ito sinimulan?*** *(See code R2.2)*	How many housing units were built since the program started? ***Ilang housing units ang naitayo mula nang magsimula ang program?***
1						
2						
3						
4						
6						
7						
8						
31						
32						

S	RISK PERCEPTION		
	**S1**	**S2**	**S3**
Shock Code	In the coming 5 years, do you think a similar [SHOCK] might occur? ***Sa susunod na 5 taon, sa palagay ninyo, magkakaroon kaya ng parehong [SHOCK]?*** 1 Yes<br>2 No	How concerned are you that a [SHOCK] might affect your city/municipality in the future, including the next generation? ***Gaano kayo nag-aalala na may [SHOCK] na maaaring makaapekto sa inyong siyudad/munisipyo sa hinaharap, kasama ang susunod na henerasyon?*** *(See code S2)*	How prepared is your city/municipality now for [SHOCK]? ***Gaano ngayon kahanda ang inyong siyudad/munisipyo sa [SHOCK]?*** *(See code S3)*
1			
2			
3			
4			
6			
7			
8			
31			
32			

R1.1 Codes for Relocation
1 Yes, Temporarily
2 Yes, Permanently
3 No

R2.2 Codes for Start of Housing
1 Less than 1 month after [SHOCK]
2 2 to 3 months after [SHOCK]
3 3 to 6 months after [SHOCK]
4 7 months to 1 year after [SHOCK]
5 More than 1 year after [SHOCK]

S2 Codes for Concern
1 Not concerned at all
2 Not very concerned
3 Neither concerned nor unconcerned
4 Fairly concerned
5 Very concerned

S3 Codes for Preparedness
1 Not prepared at all
2 Not very prepared
3 Neither prepared nor unprepared
4 Fairly prepared
5 Very prepared

Ravago, Mapa, Sunglao, Aycardo, (2020). Mendeley Data, doi: 10.17632/9y2yhw45zt.1

ASK THIS SECTION IF RESPONDENT ANSWERED CODES 1 OR 2 IN B2.3, OTHERWISE SKIP TO THE NEXT SECTION.

T	HARM MITIGATION: AGRICULTURE AND FISHERIES													
Shock code	**T1**<br>Were any agricultural products destroyed or damaged due to the [SHOCK]?<br>***Meron bang anumang produktong agrikultura na nasira o napinsala dahil sa [SHOCK]?***													
	**T1.1**<br>1 Yes<br>2 No<br>►U1	**T1.2** Which of the following products and all others including machineries and equipment?<br>***Alin sa mga sumusunod na produkto at iba pang bagay gaya ng machineries at equipment?***												
		**T1.3** How much have been destroyed or damaged?<br>***Gaano karami ang nasira o napinsala?***					**T1.4** How much is the value of the destroyed/damaged products and all others including machineries and equipment?<br>***Magkano ang halaga ng nasira o napinsalang mga produkto at iba pang bagay gaya ng machineries at equipment?***							
		(For T1.2, encircle all that apply. Indicate answers for T1.3 and T1.4 on the blanks under the items.)												
		**1**<br>Rice	**2**<br>Corn	**3**<br>Coconut	**4**<br>Banana	**5**<br>Pineapple	**6**<br>Sugarcane	**7**<br>Other grains, fruits, vegetables	**8**<br>Poultry	**9**<br>Livestock	**10**<br>Fisheries	**11**<br>Other animals	**12**<br>Agricultural equipment	**13**<br>Irrigation facilities
1		**1**<br>Qty ________<br>Value ________	**2**<br>Qty ________<br>Value ________	**3**<br>Qty ________<br>Value ________	**4**<br>Qty ________<br>Value ________	**5**<br>Qty ________<br>Value ________	**6**<br>Qty ________<br>Value ________	**7**<br>Qty ________<br>Value ________	**8**<br>Qty ________<br>Value ________	**9**<br>Qty ________<br>Value ________	**10**<br>Qty ________<br>Value ________	**11**<br>Qty ________<br>Value ________	**12**<br>Qty ________<br>Value ________	**13**<br>Qty ________<br>Value ________
2		**1**<br>Qty ________<br>Value ________	**2**<br>Qty ________<br>Value ________	**3**<br>Qty ________<br>Value ________	**4**<br>Qty ________<br>Value ________	**5**<br>Qty ________<br>Value ________	**6**<br>Qty ________<br>Value ________	**7**<br>Qty ________<br>Value ________	**8**<br>Qty ________<br>Value ________	**9**<br>Qty ________<br>Value ________	**10**<br>Qty ________<br>Value ________	**11**<br>Qty ________<br>Value ________	**12**<br>Qty ________<br>Value ________	**13**<br>Qty ________<br>Value ________
3		**1**<br>Qty ________<br>Value ________	**2**<br>Qty ________<br>Value ________	**3**<br>Qty ________<br>Value ________	**4**<br>Qty ________<br>Value ________	**5**<br>Qty ________<br>Value ________	**6**<br>Qty ________<br>Value ________	**7**<br>Qty ________<br>Value ________	**8**<br>Qty ________<br>Value ________	**9**<br>Qty ________<br>Value ________	**10**<br>Qty ________<br>Value ________	**11**<br>Qty ________<br>Value ________	**12**<br>Qty ________<br>Value ________	**13**<br>Qty ________<br>Value ________
4		**1**<br>Qty ________<br>Value ________	**2**<br>Qty ________<br>Value ________	**3**<br>Qty ________<br>Value ________	**4**<br>Qty ________<br>Value ________	**5**<br>Qty ________<br>Value ________	**6**<br>Qty ________<br>Value ________	**7**<br>Qty ________<br>Value ________	**8**<br>Qty ________<br>Value ________	**9**<br>Qty ________<br>Value ________	**10**<br>Qty ________<br>Value ________	**11**<br>Qty ________<br>Value ________	**12**<br>Qty ________<br>Value ________	**13**<br>Qty ________<br>Value ________
6		**1**<br>Qty ________<br>Value ________	**2**<br>Qty ________<br>Value ________	**3**<br>Qty ________<br>Value ________	**4**<br>Qty ________<br>Value ________	**5**<br>Qty ________<br>Value ________	**6**<br>Qty ________<br>Value ________	**7**<br>Qty ________<br>Value ________	**8**<br>Qty ________<br>Value ________	**9**<br>Qty ________<br>Value ________	**10**<br>Qty ________<br>Value ________	**11**<br>Qty ________<br>Value ________	**12**<br>Qty ________<br>Value ________	**13**<br>Qty ________<br>Value ________
7		**1**<br>Qty ________<br>Value ________	**2**<br>Qty ________<br>Value ________	**3**<br>Qty ________<br>Value ________	**4**<br>Qty ________<br>Value ________	**5**<br>Qty ________<br>Value ________	**6**<br>Qty ________<br>Value ________	**7**<br>Qty ________<br>Value ________	**8**<br>Qty ________<br>Value ________	**9**<br>Qty ________<br>Value ________	**10**<br>Qty ________<br>Value ________	**11**<br>Qty ________<br>Value ________	**12**<br>Qty ________<br>Value ________	**13**<br>Qty ________<br>Value ________
8		**1**<br>Qty ________<br>Value ________	**2**<br>Qty ________<br>Value ________	**3**<br>Qty ________<br>Value ________	**4**<br>Qty ________<br>Value ________	**5**<br>Qty ________<br>Value ________	**6**<br>Qty ________<br>Value ________	**7**<br>Qty ________<br>Value ________	**8**<br>Qty ________<br>Value ________	**9**<br>Qty ________<br>Value ________	**10**<br>Qty ________<br>Value ________	**11**<br>Qty ________<br>Value ________	**12**<br>Qty ________<br>Value ________	**13**<br>Qty ________<br>Value ________
31		**1**<br>Qty ________<br>Value ________	**2**<br>Qty ________<br>Value ________	**3**<br>Qty ________<br>Value ________	**4**<br>Qty ________<br>Value ________	**5**<br>Qty ________<br>Value ________	**6**<br>Qty ________<br>Value ________	**7**<br>Qty ________<br>Value ________	**8**<br>Qty ________<br>Value ________	**9**<br>Qty ________<br>Value ________	**10**<br>Qty ________<br>Value ________	**11**<br>Qty ________<br>Value ________	**12**<br>Qty ________<br>Value ________	**13**<br>Qty ________<br>Value ________
32		**1**<br>Qty ________<br>Value ________	**2**<br>Qty ________<br>Value ________	**3**<br>Qty ________<br>Value ________	**4**<br>Qty ________<br>Value ________	**5**<br>Qty ________<br>Value ________	**6**<br>Qty ________<br>Value ________	**7**<br>Qty ________<br>Value ________	**8**<br>Qty ________<br>Value ________	**9**<br>Qty ________<br>Value ________	**10**<br>Qty ________<br>Value ________	**11**<br>Qty ________<br>Value ________	**12**<br>Qty ________<br>Value ________	**13**<br>Qty ________<br>Value ________

Ravago, Mapa, Sunglao, Aycardo, (2020). Mendeley Data, doi: 10.17632/9y2yhw45zt.1

ASK THIS SECTION IF RESPONDENT ANSWERED CODES 1 OR 2 IN B2.3, OTHERWISE SKIP TO SECTION THE NEXT SECTION.

T	HARM MITIGATION: AGRICULTURE AND FISHERIES									
	T2	T3	T4		T5	T6	T7	T8		T9
			T4.1	T4.2				Were any agricultural product transferred to the public warehouse due to the [SHOCK]? ***Nilipat ba ang anumang produktong agrikultura sa pampublikong warehouse dahil sa [SHOCK]?***		
	Did you encourage crop switching? ***Naghikayat ba kayo na mag-crop switching o magtanim ng ibang pananim?*** *(1-Yes 2-No)*	Did you provide climate-resistant crops or seeds? ***Namahagi ba kayo ng mga climate-resistant na pananim o binhi?*** *(1-Yes 2-No)*	Did you distribute fertilizers, medicines and other supplies to farmers? ***Namahagi o nag-distribute ba kayo ng mga pataba at iba bang mga supplies sa mga magsasaka?*** *(1-Yes 2-No)*	Which of the following did you distribute? ***Alin sa mga sumusunod ang ipinamahagi ninyo?*** *(Encircle all that apply)*	Did you implement cloud seeding to your city/municipality? ***Nagpa-cloud seeding ba kayo sa inyong siyudad/munisipyo?*** *(1-Yes, 2-No)*	Did you improve farmland irrigation? ***Inimprove ba ang irigasyon sa farmland?*** *(1-Yes, 2-No)*	Is there a public warehouse designated for storing for agricultural products in time of [SHOCK]? ***May pampublikong warehouse ba na paglilipatan ng mga produktong agrikultura kapag may [SHOCK]?*** *(1-Yes, 2-No)*	**T8.1** 1 Yes 2 No ►U1	**T8.2** Which of the following products? ***Alin sa mga sumusunod na produkto?*** **T8.3** How much were transferred? ***Gaano karami ang nilipat?*** *(See code T8.2. Encircle all that apply. Indicate answers for T8.3. on the blank beside the code.)*	How many days was the warehouse used when the [SHOCK] occurred? ***Gaano katagal itinago sa warehouse noong nagkaroon ng [SHOCK]?*** *(See code T9)*
1				1 2 / 3 4 / 5					1____ 2____ 3____ 4____	
2				1 2 / 3 4 / 5					1____ 2____ 3____ 4____	
3				1 2 / 3 4 / 5					1____ 2____ 3____ 4____	
4				1 2 / 3 4 / 5					1____ 2____ 3____ 4____	
6				1 2 / 3 4 / 5					1____ 2____ 3____ 4____	
7				1 2 / 3 4 / 5					1____ 2____ 3____ 4____	
8				1 2 / 3 4 / 5					1____ 2____ 3____ 4____	
31				1 2 / 3 4 / 5					1____ 2____ 3____ 4____	
32				1 2 / 3 4 / 5					1____ 2____ 3____ 4____	

T4.2 Supplies
1 Fertilizer
2 Pesticide
3 Livestock feed
4 Livestock medicine
5 Others

T8.2 Codes for Agricultural Products
1 Rice
2 Fruits and vegetables
3 Animals
4 Equipment

S4 Codes for Length
1 Less than 1 day
2 1 to 3 days
3 4 days to 1 week
4 More than 1 week to 1 month
5 More than 1 month to 6 months
6 More than 6 months to 1 year
7 More than 1 year

Ravago, Mapa, Sunglao, Aycardo, (2020). Mendeley Data, doi: 10.17632/9y2yhw45zt.1

U		DRRM TRAININGS

T1 — Trainings Received

U1.1 Did your office members receive any training or seminar in managing shocks? ***Nakatanggap ba ng training o seminar ang mga miyembro ng inyong opisina na tungkol sa pag-manage ng mga sakuna?***
1 Yes 2 No ►S2

U1.2 Which of the following types of training did your personnel receive? ***Alin sa mga sumusunod na klase ng training ang natanggap ng inyong personnel? Please select all that apply.***
U1.3 When did your personnel receive the [MENTION TYPE OF TRAINING]? ***Kailan natanggap ng inyong personnel ang [MENTION TYPE OF TRAINING]? Note: Ask for the earliest received.***

TYPE OF TRAINING	Month	Year		Month	Year		Month	Year		Month	Year
1 DRRM Structures, Systems, Processes	_____	_______	3 Prevention and Mitigation	_____	_______	5 Early Warning	_____	_______	7 Relief	_____	_______
2 Pre-Disaster Risk Assessment	_____	_______	4 Evacuation	_____	_______	6 Search and Rescue	_____	_______	8 Recovery	_____	_______

U1.4 Which of the following received the [MENTION TYPE OF TRAINING]? ***Sino sa mga sumusunod ang nakatanggap ng [MENTION TYPE OF TRAINING]? Encircle all that apply.***

TYPE OF TRAINING	Office of the Mayor	Office of the Governor	LDRRM Officer	Municipal Officer	Provincial Officer	Others, specify
1 DRRM Structures, Systems, Processes	1	2	3	4	5	6 __________
2 Pre-Disaster Risk Assessment	1	2	3	4	5	6 __________
3 Prevention and Mitigation	1	2	3	4	5	6 __________
4 Evacuation	1	2	3	4	5	6 __________
5 Early Warning	1	2	3	4	5	6 __________
6 Search and Rescue	1	2	3	4	5	6 __________
7 Relief	1	2	3	4	5	6 __________
8 Recovery	1	2	3	4	5	6 __________

U1.5 Who conducted the training? ***Sino ang nag-conduct ng training?***

TYPE OF TRAINING	AFP-OCD	DILG	DSWD	PAG-ASA/DOST	Provincial govt	Other govt agency	Other NGO
1 DRRM Structures, Systems, Processes	1	2	3	4	5	6 ________	7 _________
2 Pre-Disaster Risk Assessment	1	2	3	4	5	6 ________	7 _________
3 Prevention and Mitigation	1	2	3	4	5	6 ________	7 _________
4 Evacuation	1	2	3	4	5	6 ________	7 _________
5 Early Warning	1	2	3	4	5	6 ________	7 _________
6 Search and Rescue	1	2	3	4	5	6 ________	7 _________
7 Relief	1	2	3	4	5	6 ________	7 _________
8 Recovery	1	2	3	4	5	6 ________	7 _________

U1.6 Was the training useful? ***Nagagamit ba ninyo ang training?***

TYPE OF TRAINING	YES	NO		YES	NO		YES	NO		YES	NO
1 DRRM Structures, Systems, Processes	1	2	3 Prevention & Mitigation	1	2	5 Early Warning	1	2	7 Relief	1	2
2 Pre-Disaster Risk Assessment	1	2	4 Evacuation	1	2	6 Search and Rescue	1	2	8 Recovery	1	2

U1.7 Did your city/municipality receive training or seminar on insurance that the constituents can avail after a shock?
Nakatanggap ba ang inyong siyudad/munisipyo ng training o seminar tungkol sa insurance na maaaring i-avail ng mga constituents pagkatapos ng isang shock?
1 Yes 2 No ►U1.10

U1.8 After the training o seminar, did your constituents avail of insurance such as property, car, crop, or fire insurance?
Pagkatapos ng training o seminar, nag-avail ba ang inyong mga constituents ng insurance gaya ng property, car, crop, o fire insurance?
1 Yes ►U1.11 2 No ►U1.9

U1.9 Do they plan to avail?
May plano ba silang mag-avail?
1 Yes ►U1.11 2 No ►U1.11

U1.10 Do you plan on requesting such training or seminar?
May plano ba kayong humingi ng ganoong training o seminar?
1 Yes 2 No

U1.11 Did your city/municipality receive training or seminar on loans that the constituents can avail after a shock?
Nakatanggap ba ang inyong siyudad/munisipyo ng training o seminar tungkol sa loans o pautang na maaaring i-avail ng mga constituents pagkatapos ng isang shock?
1 Yes 2 No ►U1.14

U1.12 After the training o seminar, did your constituents apply for such loans?
Pagkatapos ng training o seminar, nag-apply ba sa ganoong klase ng pautang ang inyong mga constituents?
1 Yes ►U2.1 2 No ►U1.13

U1.13 Do they plan to apply for such loans?
May plano ba silang mag-apply sa ganoong klase ng pautang?
1 Yes ►U2.1 2 No ►U2.1

U1.14 Do you plan on requesting such training or seminar?
May plano ba kayong humingi ng ganoong training o seminar?
1 Yes 2 No

Ravago, Mapa, Sunglao, Aycardo, (2020). Mendeley Data, doi: 10.17632/9y2yhw45zt.1

U	DRRM TRAININGS

U2 — Trainings Conducted

U2.1 Did your office provide any training or seminar in managing shocks? ***Nagbigay ba ng anumang training o seminar ang inyong opisina na tungkol sa pag-manage ng mga sakuna?***
1 Yes 2 No ►T1

U2.1 Which of the following types of training did your office conduct? ***Alin sa mga sumusunod na klase ng training ang isinagawa ng inyong personnel?*** ***Encircle all that apply.***
U2.3 When did your office conduct the [MENTION TYPE OF TRAINING]? ***Kailan isinagawa ng inyong opisina ang [MENTION TYPE OF TRAINING]? Note: Ask for earliest conducted.***

TYPE OF TRAINING	Month	Year		Month	Year		Month	Year
1 Prevention and Mitigation	_____	_______	4 Early Warning	_____	_______	7 Recovery	_____	_______
2 Information and Awareness	_____	_______	5 Search and Rescue	_____	_______			
3 Evacuation			6 Relief	_____	_______			

U2.4 Which of the following received the [MENTION TYPE OF TRAINING]? ***Sino sa mga sumusunod ang nakatanggap ng [MENTION TYPE OF TRAINING]?*** ***Encircle all that apply.***

TYPE OF TRAINING	Barangay officials	Local constituents	Disaster volunteers	Other LGUs, specify	Other NGOs, specify	Others, specify
1 Prevention and Mitigation	1	2	3	4 __________	5 __________	6 __________
2 Information and Awareness	1	2	3	4 __________	5 __________	6 __________
3 Evacuation	1	2	3	4 __________	5 __________	6 __________
4 Early Warning	1	2	3	4 __________	5 __________	6 __________
5 Search and Rescue	1	2	3	4 __________	5 __________	6 __________
6 Relief	1	2	3	4 __________	5 __________	6 __________
7 Recovery	1	2	3	4 __________	5 __________	6 __________

U2.5 Did your city/municipality conduct training or seminar on insurance that the constituents can avail after a shock?
Nagbigay ba ang inyong siyudad/munisipyo ng training o seminar tungkol sa insurance na maaaring i-avail ng mga constituents pagkatapos ng isang shock?
1 Yes 2 No ►U2.8

U2.6 After the training o seminar, did your constituents avail of insurance such as property, car, crop, or fire insurance?
Pagkatapos ng training o seminar, nag-avail ba ang inyong mga constituents ng insurance gaya ng property, car, crop, o fire insurance?
1 Yes ►U2.9 2 No ►U2.7

U2.7 Do they plan to avail?
May plano ba silang mag-avail?
1 Yes ►U2.10 2 No ►U2.10

U2.8 Do you plan conducting such training or seminar?
May plano ba kayong magbigay ng ganoong training o seminar?
1 Yes 2 No

U2.9 Did your city/municipality conduct training or seminar on loans that the constituents can avail after a shock?
Nagbigay ba ang inyong siyudad/munisipyo ng training o seminar tungkol sa loans o pautang na maaaring i-avail ng mga constituents pagkatapos ng isang shock?
1 Yes 2 No ►U2.12

U2.10 After the training o seminar, did your constituents apply for such loans?
Pagkatapos ng training o seminar, nag-apply ba sa ganoong klase ng pautang ang inyong mga constituents?
1 Yes ►V1 2 No ►U2.11

U2.11 Do they plan to apply for such loans?
May plano ba silang mag-apply sa ganoong klase ng pautang?
1 Yes ►V1 2 No ►V1

U2.12 Do you plan conducting such training or seminar?
May plano ba kayong magbigay ng ganoong training o seminar?
1 Yes 2 No

NOTE: This section shall be pre-accomplished by the respondent. Responses in the sheet should be attached and verified by the enumerator before proceeding to the next section.

V	ASSETS

V1

V1.1 Do you have any of the following vehicles used during times of shocks?
Meron ba kayo ng alinman sa sumusunod na sasakyan sa panahon ng shocks?
V1.2 How many (ANSWER IN V1.1) do you currently have?
Ilang (ANSWER IN V3.1) ang kasalukuyang meron kayo?
V1.3 Since 2009/2012, how often did you use (ANSWER IN V1.1) during times of shocks?
Mula 2009/2012, gaano kadalas ninyo ginamit ang (ANSWER IN V1.1) sa panahon ng shocks? (See codes below)
1 Often ***Madalas*** 2 Sometimes ***Minsan*** 3 Never ***Hindi nagamit***

ITEMS	QUANTITY	FREQ. OF USE	ITEMS	QUANTITY	FREQ. OF USE	ITEMS	QUANTITY	FREQ. OF USE
1 Boats	___________	___________	5 Ambulance	___________	___________	9 Firetruck	___________	___________
2 Vans	___________	___________	6 Amphibian	___________	___________	10 Crawler	___________	___________
3 Truck	___________	___________	7 Backhoe or Scoop loader	___________	___________	11 Tractor	___________	___________
4 Bus	___________	___________	8 Dump truck	___________	___________	12 Others, specify: __________	___________	___________

V2

V2.1 Do you have any of the following emergency shelter supplies?
Meron ba kayo ng alinman sa sumusunod na emergency shelter supplies?
V2.2 Since 2009/2012, how often did you distribute (ANSWER IN V2.1) during times of shocks?
Mula 2009/2012, gaano kadalas kayo nag-distribute ng (ANSWER IN V2.1) sa panahon ng shocks? (See codes below)
1 Often ***Madalas*** 2 Sometimes ***Minsan*** 3 Never ***Hindi nagamit***

ITEMS		FREQ. OF USE	ITEMS		FREQ. OF USE	ITEMS		FREQ. OF USE
1 Jackets or Raincoats		___________	3 Beds		___________	5 Mosquito nets		___________
2 Clothes		___________	4 Beddings (blankets, etc.)		___________	6 Kitchen supplies		___________

V3

V3.1 Do you have any of the following facilities and resources?
Meron ba kayo ng alinman sa sumusunod na pasilidad at resources?
V3.2 How many (ANSWER IN V3.1) do you currently have?
Ilang (ANSWER IN V3.1) ang kasalukuyang meron kayo?
V3.3 Since 2009/2012, how often did you use (ANSWER IN V3.1) during times of shocks?
Mula 2009/2012, gaano kadalas ninyo ginamit ang (ANSWER IN V3.1) sa panahon ng shocks? (See codes below)
1 Often ***Madalas*** 2 Sometimes ***Minsan*** 3 Never ***Hindi nagamit***

ITEMS	QUANTITY	FREQ. OF USE	ITEMS	QUANTITY	FREQ. OF USE
1 Portable/Solar-powered generator	___________	___________	2 Mobile water treatment	___________	___________

V4

V4.1 Do you have any of the following search and rescue equipment?
Meron ba kayo ng alinman sa sumusunod na search and rescue equipment?
V4.2 Since 2009/2012, how often did you use (ANSWER IN V4.1) during times of shocks?
Mula 2009/2012, gaano kadalas ninyo ginamit ang (ANSWER IN V4.1) sa panahon ng shocks? (See codes below)
1 Often ***Madalas*** 2 Sometimes ***Minsan*** 3 Never ***Hindi nagamit***

ITEMS		FREQ. OF USE	ITEMS		FREQ. OF USE	ITEMS		FREQ. OF USE
1 Siren, megaphone, or whistle		___________	5 Search light or flashlight		___________	9 Extraction kit (spine board, shovel, jackhammer)		___________
2 Two-way radio		___________	6 Ladders		___________			
3 GPS device		___________	7 Helmets		___________	10 Caution tape		___________
4 Ropes		___________	8 Life vest/ reflectorized vest		___________	11 Others, specify: __________		___________

Ravago, Mapa, Sunglao, Aycardo, (2020). Mendeley Data, doi: 10.17632/9y2yhw45zt.1

NOTE: This section shall be pre-accomplished by the respondent. Responses in the sheet should be attached and verified by the enumerator before proceeding to the next section.

V	ASSETS
V5	**V5.1** Do you have the following equipment for information and awareness during times of shocks?<br>***Meron ba kayo ng alinman sa sumusunod para sa pagkuha ng impormasyon sa panahon ng shocks?***<br>**V5.2** Since 2009/2012, how often did you use (ANSWER IN V5.1) during times of shocks?<br>***Mula 2009/2012, gaano kadalas ninyo ginamit ang (ANSWER IN V5.1) sa panahon ng shocks? (See codes below)***<br>1 Often ***Madalas*** 2 Sometimes ***Minsan*** 3 Never ***Hindi nagamit***

ITEMS		FREQ. OF USE	ITEMS		FREQ. OF USE	ITEMS	QUANTITY	FREQ. OF USE
1 Phones		___________	3 Internet connection		___________	5 Others, specify: __________		___________
2 Laptops		___________	4 Batteries and Power banks		___________			

V	ASSETS
V6	**V6.1** Do you stockpile relief goods?<br>***Nagsa-stockpile ba kayo ng mga relief goods?***<br>1 Yes 2 No<br>**V6.2** Do you have a memorandum of agreement with any grocery store, supermarket, etc.?<br>***May memorandum of agreement ba kayo sa anumang grocery store, supermarket, atbp.?***<br>1 Yes 2 No<br>**V6.3** Since 2009/2012, how often did you distribute (ANSWER IN V6.1) during times of shocks?<br>***Mula 2009/2012, gaano kadalas kayo nag-distribute ng (ANSWER IN V6.1) sa panahon ng shocks? (See codes below)***<br>1 Often ***Madalas*** 2 Sometimes ***Minsan*** 3 Never ***Hindi nagamit***<br>**V6.4** Do you have a warehouse for relief goods?<br>***May warehouse ba kayo para sa relief goods?***<br>1 Yes 2 No

ITEMS		FREQ. OF USE	ITEMS		FREQ. OF USE	ITEMS		FREQ. OF USE
1 Bottled water		___________	5 Ready-to-eat meals		___________	9 Soap		___________
2 Rice		___________	6 Milk for infants		___________	10 Shampoo		___________
3 Noodles		___________	7 Toothbrush		___________	11 Sanitary pad		___________
4 Canned goods		___________	8 Toothpaste		___________	12 Diaper		___________

V	ASSETS
V7	**V7.1** Do you have any of the following medical supplies?<br>***Meron ba kayo ng alinman sa sumusunod na medical supplies?***<br>**V7.2** Since 2009/2012, how often did you distribute (ANSWER IN V7.1) during times of shocks?<br>***Mula 2009/2012, gaano kadalas kayo nagdistribute ng (ANSWER IN V7.1) sa panahon ng shocks? (See codes below)***<br>1 Often ***Madalas*** 2 Sometimes ***Minsan*** 3 Never ***Hindi nagamit***

ITEMS		FREQ. OF USE	ITEMS		FREQ. OF USE	ITEMS		FREQ. OF USE
1 First-aid kits		___________	5 Dressings (for wounds)		___________	8 Gloves and Surgical Masks		___________
2 Vaccines		___________	6 Surgical instruments		___________	9 Syringes and Needles		___________
3 Cadaver bags		___________	7 Thermometers, Stethoscope, Sphygmomanometer (for blood pressure)		___________	10 Plastic bags		___________
4 Disinfectants and antiseptics		___________				11 Others, specify: __________		___________

Ravago, Mapa, Sunglao, Aycardo, (2020). Mendeley Data, doi: 10.17632/9y2yhw45zt.1

PROFILE (Part 2)

A	**Respondent Information**	A2.4 Before your present position, do you have previous experience in disaster risk management? 1 Yes 2 No<br>***Bago ang kasalukuyan mong posisyon, mayroon ba kayong karanasan sa disaster risk management?*** ***Meron*** ***Wala***
		A2.6/ A2.7 Please indicate previous role(s)/position(s) starting with the most recent one and the number of years you held each position.<br>***Pakisabi po ang mga dating role o posisyon mula sa pinakabago at kung ilang taon kayo sa bawat posisyon.***<br>A2.6 Previous Position A2.7 No. of years A2.6 Previous Position A2.7 No. of years<br>1 ____________________________________ __________ 4 ____________________________________ __________<br>2 ____________________________________ __________ 5 ____________________________________ __________<br>3 ____________________________________ __________ 6 ____________________________________ __________
B3	**DRRM Office and Personnel**	B3.1 Does your city/municipality have a DRRM office?<br>***Mayroon bang DRRM office ang inyong siyudad/munisipyo?***<br>1 Yes 2 No ►B3.3<br>B3.2 When was the DRRM office created?<br>***Kailan nabuo ang inyong DRRM office?*** ***(MM/YYYY)***<br>MM:_______ Year:___________
		B3.3 How many [MENTION ITEMS BELOW] DRRM staff do you have?<br>***Ilan ang [MENTION ITEMS BELOW] DRRM staff ang mayroon kayo?***<br>1 Full time 2 Casual ________
		B3.4 How many of below personnel do you have? ***Ilan ang mga sumusunod na personnel ang mayroon kayo?***<br>1 Disaster Monitoring _____________<br>2 Disaster Response _____________<br>3 Others (specify ______________) _____________
B6	**Catastrophe Insurance**	B6.1 Are you aware of any catastrophe bond or insurance for your city/municipality?<br>***Pamilyar ka ba sa anumang catastrophe bond o insurance para sa inyong siyudad/munisipyo?*** 1 Yes ►B6.2 2 No ►C1
		B6.2 Has your LGU availed of any catastrophe bond or insurance?<br>***Nag-avail ba ng anumang catastrophe bond insurance ang inyong LGU?***<br>1 Yes ►B6.3 2 No ►B6.4
		B6.3 What catastrophe bond or insurance for your city/municipality?<br>***Anong catastrophe bond o insurance para sa inyong siyudad/ munisipyo?***<br>____________________________________<br>B6.4 Are you considering availing any catastrophe bond or insurance for you city/municipality?<br>***May balak bang mag-avail ng anumang catastrophe bond o insurance para sa inyong siyudad/munisipyo?*** 1 Yes 2 No

Ravago, Mapa, Sunglao, Aycardo, (2020). Mendeley Data, doi: 10.17632/9y2yhw45zt.1
