# Supplementary material for: Data from a survey of the Philippines’ local governments on their risk management strategies to natural disasters
Source: Data Brief. 2020 Nov 19;33:106548. doi: 10.1016/j.dib.2020.106548 (PMC7701180; doi:10.1016/j.dib.2020.106548)
Supplement: Supplementary file 1 [file mmc1.zip › Supplementary Appendix C DIB Ravago et al 2020 sub-index equations.pdf]

Supplementary Appendix AC: Ravago, Majah-Leah; Mapa, Claire Dennis; Sunglao, Jun Carlo; Aycardo, Angelie Grace (2020), Data in Brief.

Supplementary Appendix C: Full sets of equations used to create each sub-indices.

1. Assets Index ($\overline{AI}$)

The AI is the geometric average of the 7 indices: 1.) Asset Vehicle Index (AVI), 2. Asset Emergency Shelter Index (AESI), 3. Asset Facilities and Resources Index (AFRI), 4. Asset Search and Rescue Index (ASRI), 5. Asset Information Index (AII), 6. Asset Relief Goods Index (ARGI), and 7.) Asset Medical Supplies Index (AMSI), given by

$$\overline{AI_\iota} = \sqrt[7]{AVI_{i,s} \times AESI_{i,s} \times AFRI_{i,s} \times ASRI_{i,s} \times AII_{i,s} \times ARGI_{i,s} \times AMSI_{i,s}} \times 100\%$$

where

$\overline{AI_\iota}$ = Assets Index of the ith city/ municipality

$AVI_i \equiv$ Asset Vehicle Index of ith city/municipality

$AESI_i \equiv$ Asset Emergency Shelter Index of ith city/municipality

$AFRI_i \equiv$ Asset Facilities and Resources Index of ith city/municipality

$ASRI_i \equiv$ Asset Search and Rescue Index of ith city/municipality

$AII_i \equiv$ Asset Information Index of ith city/municipality

$ARGI_i \equiv$ Asset Relief Goods Index of ith city/municipality

$AMSI_i \equiv$ Asset Medical Supplies Index of ith city/municipality

$i \equiv$ city/municipality ≡ 1, 2, 3, …, 189

$s \equiv$ type of hydro-meteorological hazard ≡ {1- Strong winds & rain, 2 - Flood, 3 - Landslide, 4 – Big waves}

1.1. Asset Vehicle Index (AVI)

The AVI is the average of the type of vehicles used by the municipality in times of hydro-meteorological hazards (Boats, Vans, Truck, Bus, Ambulance, Amphibian, Backhoe or Scoop loader, Dump truck, Firetruck, Crawler and Tractor) and the frequency of use., given by

$$AVI_i = \frac{\sum_{j=1}^{11} V_{ij} \times F_{ij}}{11 \times 3} \times 100\%$$

where

$AVI_i \equiv$ Asset Vehicle Index of ith city/municipality

$V_{ij} \equiv$ Indicator variable if the ith city/municipality has the jth vehicle type used during times of hydro-
meteorological hazards (1 – Yes, 0 – No)

$F_{ij} \equiv$ Ordinal variable on the frequency of use of the jth vehicle type by the ith city/municipality ≡ {Often, Sometimes, Never}

$i \equiv$ city/municipality ≡ 1, 2, 3, …, 189

$j \equiv$ type of vehicle ≡ {Boats, Vans, Truck, Bus, Ambulance, Amphibian, Backhoe or Scoop loader, Dump
truck, Firetruck, Crawler, Tractor}

1.2. Asset Emergency Shelter Index (AESI)

The AESI is the average of the type of emergency shelter supplies given by the municipality in times of hydro-meteorological hazards (Jackets or Raincoats, Clothes, Beds, Beddings (blankets, etc.), Mosquito nets and Kitchen supplies) and the frequency of distribution., given by

$$AESI_i = \frac{\sum_{j=1}^{6} S_{ij} \times F_{ij}}{6 \times 3} \times 100\%$$

where
$AESI_i$ ≡ Asset Emergency Shelter Index of ith city/municipality
S_{ij} ≡ Indicator variable if the ith city/municipality has the jth type of emergency shelter supply given during
times of hydro-meteorological hazards (1 – Yes, 0 – No)
F_{ij} ≡ Ordinal variable on the frequency of distribution of the jth type of emergency shelter supply given
by the ith city/municipality ≡{Often, Sometimes, Never}
i ≡ city/municipality ≡ 1, 2, 3, ..., 189
j ≡ type of emergency supply ≡ {Jackets or Raincoats, Clothes, Beds, Beddings (blankets, etc.), Mosquito
nets, Kitchen supplies}

1.3. Asset Facilities and Resources Index (AFRI)

The AFRI is the average of the type of facilities and resources used by the municipality in times of hydro-meteorological hazards (Portable/Solar-powered generator and Mobile water treatment) and the frequency of use., given by

$$AFRI_i = \frac{\sum_{j=1}^{2} R_{ij} \times F_{ij}}{2 \times 3} \times 100\%$$

where
$AFRI_i$ ≡ Asset Facilities and Resources Index of ith city/municipality
R_{ij} ≡ Indicator variable if the ith city/municipality has the jth type of facility and resources used during
times of hydro-meteorological hazards (1 – Yes, 0 – No)
F_{ij} ≡ Ordinal variable on the frequency of distribution of the jth type of facility and resources used by the ith city/municipality ≡ {Often, Sometimes, Never}
i ≡ city/municipality ≡ 1, 2, 3, ..., 189
j ≡ type of facility and resources ≡ {Portable/Solar-powered generator, Mobile water treatment}

1.4. Asset Search and Rescue Index (ASRI)

The ASRI is the average of the type of search and rescue equipment used by the municipality in times of hydro-meteorological hazards (Siren, megaphone, or whistle, Two-way radio, GPS device, Ropes, Search light or flashlight, Ladders, Helmets, Life vest/ reflectorized vest, Extraction kit (spine board, shovel, jackhammer) and Caution tape) and the frequency of use., given by

$$AFRI_i = \frac{\sum_{j=1}^{10} S_{ij} \times F_{ij}}{10 \times 3} \times 100\%$$

where
$ASRI_i$ ≡ Asset Search and Rescue Index of ith city/municipality
S_{ij} ≡ Indicator variable if the ith city/municipality has the jth type of search and rescue equipment used
during times of hydro-meteorological hazards (1 – Yes, 0 – No)
F_{ij} ≡ Ordinal variable on the frequency of use of the jth type of search and rescue equipment by the ith
city/municipality ≡≡ {Often, Sometimes, Never}
i ≡ city/municipality ≡ 1, 2, 3, ..., 189

$j \equiv$ type of search and rescue equipment ≡ {Siren, megaphone, or whistle, Two-way radio, GPS device,
Ropes, Search light or flashlight, Ladders, Helmets, Life vest/ reflectorized vest, Extraction kit
(spine board, shovel, jackhammer), Caution tape}

1.5. Asset Information Index (AII)

The AII is the average of the type of information and awareness equipment used by the municipality in times of hydro-meteorological hazards (Phones, Laptops, Internet connection, Batteries and Power banks) and the frequency of use., given by

$$AII_i = \frac{\sum_{j=1}^{4} I_{ij} \times F_{ij}}{4 \times 3} \times 100\%$$

where
$AII_i \equiv$ Asset Information Index of ith city/municipality
$I_{ij} \equiv$ Indicator variable if the ith city/municipality has the jth type of information and awareness equipment
used during times of hydro-meteorological hazards (1 – Yes, 0 – No)
$F_{ij} \equiv$ Ordinal variable on the frequency of use of the jth type of information and awareness equipment by
the ith city/municipality ≡ ≡ {Often, Sometimes, Never}
$i \equiv$ city/municipality ≡ 1, 2, 3, ..., 189
$j \equiv$ type of information and awareness equipment ≡ {Phones, Laptops, Internet connection, Batteries and
Power banks}

1.6. Asset Relief Goods Index (ARGI)

The ARGI is the average of the type of relief goods distributed by the municipality in times of hydro-meteorological hazards (Bottled water, Rice, Noodles, Canned goods, Ready-to-eat meals, Milk for infants, Toothbrush, Toothpaste, Soap, Shampoo, Sanitary pad and Diaper) and the frequency of distribution., given by

$$ARGI_i = \frac{\sum_{j=1}^{12} G_{ij} \times F_{ij}}{12 \times 3} \times 100\%$$

where
$ARGI_i \equiv$ Asset Relief Goods Index of ith city/municipality
$G_{ij} \equiv$ Indicator variable if the ith city/municipality has the jth type of relief goods distributed during times
of hydro-meteorological hazards (1 – Yes, 0 – No)
$F_{ij} \equiv$ Ordinal variable on the frequency of distribution of the jth type of relief goods by the
i^{th} city/municipality ≡ {Often, Sometimes, Never}
$i \equiv$ city/municipality ≡ 1, 2, 3, ..., 189
$j \equiv$ type of relief goods distributed ≡ {Bottled water, Rice, Noodles, Canned goods, Ready-to-eat meals,
Milk for infants, Toothbrush, Toothpaste, Soap, Shampoo, Sanitary pad, Diaper}

1.7. Asset Medical Supplies Index (AMSI)

The AMSI is the average of the type of medical supplies distributed by the municipality in times of hydro-meteorological hazards (First-aid kits, Vaccines, Cadaver bags, Disinfectants and antiseptics, Dressings (for wounds), Surgical instruments, Thermometers, Stethoscope,

Sphygmomanometer (for blood pressure), Gloves and Surgical Masks, Syringes and Needles, and Plastic bags) and the frequency of distribution., given by

$$AMSI_i = \frac{\sum_{j=1}^{10} M_{ij} \times F_{ij}}{10 \times 3} \times 100\%$$

where

$AMSI_i$ ≡ Asset Medical Supplies Index of ith city/municipality

M_{ij} ≡ Indicator variable if the ith city/municipality has the jth type of medical supply distributed during times of hydro-meteorological hazards (1 – Yes, 0 – No)

F_{ij} ≡ Ordinal variable on the frequency of distribution of the jth type of medical supply by the ith city/municipality ≡ {Often, Sometimes, Never}

i ≡ city/municipality ≡ 1, 2, 3, ..., 189

j ≡ type of medical supply distributed ≡ {First-aid kits, Vaccines, Cadaver bags, Disinfectants and antiseptics, Dressings (for wounds), Surgical instruments, Thermometers, Stethoscope, Sphygmomanometer (for blood pressure), Gloves and Surgical Masks, Syringes and Needles, Plastic bags}

2. **Cleanup Operations Index ($\overline{COI}$)**

The $\overline{COI}$ is the weighted geometric mean of these two components: 1. Product of the indicator variable if the LGU has undertaken clean-up operations and when it started, and 2. Product of the indicator variable if the LGU has undertaken clean-up operations and duration.

First, we calculate the index for each hydro-meteorological hazard (strong winds & rain, flood, landslide and big waves),

$$COI_{i,s} = \sqrt{\frac{C_{i,s} \times S_{i,s}}{6} \times \frac{C_{i,s} \times D_{i,s}}{7}} \times 100\%$$

then the unweighted/weighted mean of the four hydro-meteorological hazards are calculated,

$$\overline{COI_i} = \left(\frac{167}{189} COI_{i,1} + \frac{20}{189} COI_{i,2} + \frac{2}{189} COI_{i,3} + \frac{0}{189} COI_{i,4}\right) \times 100\%$$

where

$\overline{COI_i}$≡ Weighted Clean-up Operations Index of ith city/municipality

$COI_{i,s}$ ≡ Clean-up Operations Index of ith city/municipality for s^{th} hydro-meteorological hazard

$C_{i,s}$ ≡ Indicator variable if the ith city/municipality has undertaken clean-up operations for the sth hydro-meteorological hazard (1 – Yes, 0 – No)

$S_{i,s}$ ≡ Ordinal variable when the ith city/ municipality started the clean-up operations for the sth hydro-meteorological hazard ≡ {1, 2, 3, ..., 6}

$D_{i,s}$ ≡ Duration of clean-up operations done by the ith city/municipality for the sth hydro-meteorological hazard ≡ {Less than 1 day, 1 to 3 days, 4 days to 1 week, More than 1 week to 1 month, More than 1 month to 6 months, More than 6 months to 1 year, More than 1 year}

i ≡ city/municipality ≡ 1, 2, 3, ..., 189

s ≡ type of hydro-meteorological hazard ≡ {1- Strong winds & rain, 2 - Flood, 3 - Landslide, 4- Big waves}

3. Disaster Effects to Constituents Index ($\overline{DECI}$)

The $\overline{DECI}$ is the weighted average of the product of the indicator variable if the hydro-meteorological hazard resulted in death, illness or injury of the constituents and the types of effects (Death, Illness and Injury).

First, we calculate the index for each hydro-meteorological hazard (strong winds & rain, flood, landslide and big waves),

$$DECI_{i,s} = \frac{\sum_{j=1}^{3} R_{i,s} \times E_{ij,s}}{3} \times 100\%$$

then the weighted mean of the four hydro-meteorological hazards are calculated,

$$\overline{DECI_i} = \left(\frac{167}{189} DECI_{i,1} + \frac{20}{189} DECI_{i,2} + \frac{2}{189} DECI_{i,3} + \frac{0}{189} DECI_{i,4}\right) \times 100\%$$

where

$\overline{DECI_i}$≡ Weighted Disaster Effects to Constituents Index of ith city/municipality

$DECI_i$ ≡ Disaster Effects to Constituents Index of ith city/municipality

$DECI_{i,s}$ ≡ Disaster Effects to Constituents Index of ith city/municipality for s^{th} hydro-meteorological hazard

$R_{i,s}$ ≡ Indicator variable if the hydro-meteorological hazard resulted in death, illness or injury of constituents of the ith city/municipality for the sth hydro-meteorological hazard (1 – Yes, 0 – No)

$E_{ij,s}$ ≡ Indicator variable of the effects of the hydro-meteorological hazard to the constituents of the ith city/municipality for s^{th} hydro-meteorological hazard (1 – Yes, 0 – No)

j ≡ types of effects of the hydro-meteorological hazard ≡ {Death, Illness, Injury}

i ≡ city/municipality ≡ 1, 2, 3, ..., 189

s ≡ type of hydro-meteorological hazard ≡ {1- Strong winds & rain, 2 - Flood, 3 – Landslide, 4 – Big waves}

4. Employment Index ($\overline{EMI}$)

The $\overline{EI}$ is the weighted geometric mean of the product of two components: 1.) Product of the indicator variable if the LGU has a cash-for-work program for the hydro-meteorological hazard and the daily wage rate (Less than Php 150, Php 150-300, Php 301-450, Php 451-600 and More than Php 600); and 2.) Product of the indicator variable if the LGU has a food-for-work program for the hydro-meteorological hazard and the value of the food for a day's work (Less than Php 150, Php 150-300, Php 301-450, Php 451-600 and More than Php 600).

First, we calculate the index for each hydro-meteorological hazard (strong winds & rain, flood, landslide and big waves),

$$EMI_{i,s} = \sqrt{\frac{C_{i,s} \times W_{i,s}}{5} \times \frac{F_{i,s} \times V_{i,s}}{5}} \times 100\%$$

then the weighted mean of the four hydro-meteorological hazards are calculated,

$$\overline{EMI_i} = \left(\frac{167}{189} EI_{i,1} + \frac{20}{189} EI_{i,2} + \frac{2}{189} EI_{i,3} + \frac{0}{189} EI_{i,4}\right) \times 100\%$$

where

$\overline{EMI_i}$≡ Weighted Employment Index of ith city/municipality

$EMI_{i,s}$ ≡ Employment Index of ith city/municipality for s^{th} hydro-meteorological hazard

$C_{i,s}$ ≡ Indicator variable if the ith city/municipality has a cash-for-work program for the sth hydro-meteorological hazard (1 – Yes, 0 – No)

$W_{i,s}$ ≡ Ordinal variable for the daily wage rate offered by the ith city/ municipality for the sth hydro-meteorological hazard ≡ {1, 2, 3, ..., *5*}

$F_{i,s}$ ≡ Indicator variable if the ith city/municipality has a food-for-work program for the sth hydro-meteorological hazard (1 – Yes, 0 – No)
$V_{i,s}$ ≡ Ordinal variable for the daily food value offered by the ith city/ municipality for the sth hydro-meteorological hazard ≡ {Less than Php 150, Php 150-300, Php 301-450, Php 451-600,
More than Php 600}
i ≡ city/municipality ≡ 1, 2, 3, ..., 189
s ≡ type of hydro-meteorological hazard ≡ {1- Strong winds & rain, 2 - Flood, 3 - Landslide, 4- Big waves}

5. Evacuation Index ($\overline{EI}$)

The $\overline{EI}$ is the weighted geometric mean of three indices: 1.) Evacuation Order Index (EOI), 2.) Evacuation Center Index (ECI), and 3.) Evacuation Center Facilities Index (ECFI).

First, we calculate the arithmetic mean of the index for each hydro-meteorological hazard (strong winds & rain, flood, landslide and big waves).

$$EI_{i,s} = \left(\sqrt[3]{EOI_{i,s} + ECI_{i,s} + ECFI_{i,s}}\right) \times 100\%$$

then the weighted mean of the four hydro-meteorological hazards are calculated,

$$\overline{EI_i} = \left(\frac{167}{189}EI_{i,1} + \frac{20}{189}EI_{i,2} + \frac{2}{189}EI_{i,3} + \frac{0}{189}EI_{i,4}\right) \times 100\%$$

where
$\overline{EI_i}$≡ Weighted Evacuation Index of ith city/municipality
$EI_{i,s}$ ≡ Evacuation Index of ith city/municipality for s^{th} hydro-meteorological hazard
$EOI_{i,s}$ ≡ Evacuation Order Index of ith city/municipality for s^{th} hydro-meteorological hazard
$ECI_{i,s}$ ≡ Evacuation Center Index of ith city/municipality for s^{th} hydro-meteorological hazard
$ECFI_{i,s}$ ≡ Evacuation Center Facilities Index of ith city/municipality for s^{th} hydro-meteorological hazard
i ≡ city/municipality ≡ 1, 2, 3, ..., 189
s ≡ type of hydro-meteorological hazard ≡ {1- Strong winds & rain, 2 - Flood, 3 – Landslide, 4 – Big waves}

5.1. Evacuation Order Index (EOI)

The EOI is the average of product of presence of evacuation order issued to the constituents and time it was issued.

First, we calculate the index for each hydro-meteorological hazard (strong winds & rain, flood, landslide and big waves).

$$EOI_{i,s} = \frac{O_{i,s} \times T_{i,s}}{5} \times 100\%$$

then the weighted mean of the four hydro-meteorological hazards are calculated,

$$\overline{EOI_i} = \left(\frac{167}{189}EOI_{i,1} + \frac{20}{189}EOI_{i,2} + \frac{2}{189}EOI_{i,3} + \frac{0}{189}EOI_{i,4}\right) \times 100\%$$

where
$\overline{EOI_i}$≡ Weighted Evacuation Order Index of ith city/municipality
$EOI_{i,s}$ ≡ Evacuation Order Index of ith city/municipality for s^{th} hydro-meteorological hazard
$O_{i,s}$ ≡ Indicator variable if the ith city/municipality issue an order for evacuation to constituents for s^{th}
hydro-meteorological hazard (1 – Yes, 0 – No)
$T_{i,s}$ ≡ Ordinal variable for the time of implementation of evacuation order to the constituents by i^{th}

city/municipality for s^{th} hydro-meteorological hazard ≡ {More than 24 hours before disaster, 24
hours or less before disaster, During disaster, Less than 24 hours after disaster, More than 24 hours
after disaster}
i ≡ city/municipality ≡ 1, 2, 3, ..., 189
s ≡ type of hydro-meteorological hazard ≡ {1- Strong winds & rain, 2 - Flood, 3 – Landslide, 4 – Big waves}

5.2. Evacuation Center Index (ECI)

The ECI is the average of the product of presence of evacuation center designated for the hydro-meteorological hazard and their original use (Public school building, Public gym/basketball court, etc., Municipal hall, Church, and Private building)

First, we calculate the index for each hydro-meteorological hazard (strong winds & rain, flood, landslide and big waves),

$$ECI_{i,s} = \frac{\sum_{j=1}^{5} EC_{i,s} \times U_{ij,s}}{5} \times 100\%$$

then the weighted mean of the four hydro-meteorological hazards are calculated,

$$\overline{ECI_i} = \left(\frac{167}{189} ECI_{i,1} + \frac{20}{189} ECI_{i,2} + \frac{2}{189} ECI_{i,3} + \frac{0}{189} ECI_{i,4}\right) \times 100\%$$

where
$\overline{ECI_i}$≡ Weighted Evacuation Center Index of ith city/municipality
$ECI_{i,s}$ ≡ Evacuation Center Index of ith city/municipality for s^{th} hydro-meteorological hazard
$EC_{i,s}$ ≡ Indicator variable if the ith city/municipality had a designated evacuation center for the sth hydro-meteorological hazard (1 – Yes, 0 – No)
$U_{ij,s}$ ≡ Indicator variable of type of original use of the evacuation center of the ith city/municipality for s^{th} hydro-meteorological hazard (1 – Yes, 0 – No)
j ≡ original use of the evacuation center ≡ {Public school building, Public gym/basketball court, etc.
Municipal hall, Church, Private building}
i ≡ city/municipality ≡ 1, 2, 3, ..., 189
s ≡ type of hydro-meteorological hazard ≡ {1- Strong winds & rain, 2 - Flood, 3 – Landslide, 4 – Big waves}

5.3. Evacuation Center Facilities Index (ECFI)

The ECFI is the average of the product of presence of evacuation center designated for the hydro-meteorological hazard and the facilities available (Toilets, Generators, Common Kitchen, Health Station and Assembly Area)

First, we calculate the index for each hydro-meteorological hazard (strong winds & rain, flood, landslide and big waves),

$$ECFI_{i,s} = \frac{\sum_{j=1}^{5} EC_{i,s} \times F_{ij,s}}{5} \times 100\%$$

then the weighted mean of the four hydro-meteorological hazards are calculated,

$$\overline{ECFI_i} = \left(\frac{167}{189} ECFI_{i,1} + \frac{20}{189} ECFI_{i,2} + \frac{2}{189} ECFI_{i,3} + \frac{0}{189} ECFI_{i,4}\right) \times 100\%$$

where
$\overline{ECFI_i}$≡ Weighted Evacuation Center Facilities Index of ith city/municipality
$ECFI_{i,s}$ ≡ Evacuation Center Facilities Index of ith city/municipality for s^{th} hydro-meteorological hazard
$EC_{i,s}$ ≡ Indicator variable if the ith city/municipality had a designated evacuation center for

the sth hydro-meteorological hazard (1 – Yes, 0 – No)
$F_{ij,s}$ ≡ Indicator variable of the facilities available in the evacuation center of the ith city/municipality for
s^{th} hydro-meteorological hazard (1 – Yes, 0 – No)
j ≡ facilities available in the evacuation center ≡ {Toilets, Generators, Common Kitchen, Health Station,
Assembly Area}
i ≡ city/municipality ≡ 1, 2, 3, ..., 189
s ≡ type of hydro-meteorological hazard ≡ {1- Strong winds & rain, 2 - Flood, 3 - Landslide, 4 – Big waves}

6. **Housing Program Index ($\overline{HPI}$)**

The $\overline{HPI}$ is the weighted average of the product of the indicator variable if the LGU has any housing programs in response to the hydro-meteorological hazard and when it was started.

First, we calculate the index for each hydro-meteorological hazard (strong winds & rain, flood, landslide and big waves),

$$HPI_{i,s} = \frac{H_{i,s} \times S_{i,s}}{5} \times 100\%$$

then the weighted mean of the four hydro-meteorological hazards are calculated,

$$\overline{HPI_i} = \left(\frac{167}{189}HPI_{i,1} + \frac{20}{189}HPI_{i,2} + \frac{2}{189}HPI_{i,3} + \frac{0}{189}HPI_{i,4}\right) \times 100\%$$

where
$\overline{HPI_i}$ ≡ Housing Program Index of ith city/municipality
$HPI_{i,s}$ ≡ Housing Program Index of ith city/municipality for s^{th} hydro-meteorological hazard
$H_{i,s}$ ≡ Indicator variable if the ith city/municipality has any housing programs in response to the
s^{th} hydro-meteorological hazard (1 – Yes, 0 – No)
$S_{i,s}$ ≡ Ordinal variable for in the time when the housing programs began in the ith
city/municipality for the sth hydro-meteorological hazard ≡ {Less than 1 month after disaster,
2 to 3 months after disaster, 3 to 6 months after disaster, 7 months to 1 year after disaster, More
than 1 year after disaster}
i ≡ city/municipality ≡ 1, 2, 3, ..., 189
s ≡ type of hydro-meteorological hazard ≡ {1- Strong winds & rain, 2 - Flood, 3 - Landslide, 4- Big waves}

7. **Infrastructure Index ($\overline{II}$)**

The $\overline{II}$ is the weighted geometric mean of two indices: 1.) Infrastructure Breakdown Index (IBI), and 2.) Infrastructure Repair Index (IRI).

First, we calculate the index for each hydro-meteorological hazard (strong winds & rain, flood, landslide and big waves),

$$II_{i,s} = \sqrt{IBI_{i,s} \times IRI_{i,s}} \times 100\%$$

then the weighted mean of the four hydro-meteorological hazards are calculated,

$$\overline{II_i} = \left(\frac{167}{189}II_{i,1} + \frac{20}{189}II_{i,2} + \frac{2}{189}II_{i,3} + \frac{0}{189}II_{i,4}\right) \times 100\%$$

where
$\overline{II_i}$ ≡ Infrastructure Index of ith city/municipality

$II_{i,s}$ ≡ Infrastructure Index of ith city/municipality for s^{th} hydro-meteorological hazard
$IBI_{i,s}$ ≡ Infrastructure Breakdown Index of ith city/municipality for s^{th} hydro-meteorological hazard
$IRI_{i,s}$ ≡ Infrastructure Repair Index of ith city/municipality for s^{th} hydro-meteorological hazard
i ≡ city/municipality ≡ 1, 2, 3, ..., 189
s ≡ type of hydro-meteorological hazard ≡ {1- Strong winds & rain, 2 - Flood, 3 - Landslide, 4- Big waves}

7.1. Infrastructure Breakdown Index (IBI)

The IBI is the weighted average of the product of the indicator variable if the LGU had infrastructure breakdowns during/ after the hydro-meteorological hazard and the types of infrastructures that broke down (LDRRM office, Municipal hall, Health office, health center, Public school, Public gym, Other government-owned buildings, Public equipment, Public vehicles, Bridges, roads, Water facilities, Electrical facilities and Communication facilities (e.g. cell sites)).

First, we calculate the index for each hydro-meteorological hazard (strong winds & rain, flood, landslide and big waves),

$$IBI_{i,s} = \frac{\sum_{j=1}^{12} B_{i,s} \times I_{ij,s}}{12} \times 100\%$$

then the weighted mean of the four hydro-meteorological hazards are calculated,

$$\overline{IBI_i} = \left(\frac{167}{189} IBI_{i,1} + \frac{20}{189} IBI_{i,2} + \frac{2}{189} IBI_{i,3} + \frac{0}{189} IBI_{i,4}\right) \times 100\%$$

where
$\overline{IBI_i}$ ≡ Infrastructure Breakdown Index of ith city/municipality
$IBI_{i,s}$ ≡ Infrastructure Breakdown Index of ith city/municipality for s^{th} hydro-meteorological hazard
$B_{i,s}$ ≡ Indicator variable if the ith city/municipality had infrastructure breakdown before/ during
the sth hydro-meteorological hazard (1 – Yes, 0 – No)
$I_{ij,s}$ ≡ Indicator variable of the type of infrastructure that broke down in the ith city/municipality
before /during the sth hydro-meteorological hazard (1 – Yes, 0 – No)
j ≡ type of infrastructure ≡ {LDRRM office, Municipal hall, Health office, health center, Public school,
Public gym, Other government-owned buildings, Public equipment, Public vehicles, Bridges,
roads, Water facilities, Electrical facilities and Communication facilities (e.g. cell sites)}
i ≡ city/municipality ≡ 1, 2, 3, ..., 189
s ≡ type of hydro-meteorological hazard ≡ {1- Strong winds & rain, 2 - Flood, 3 - Landslide, 4- Big waves}

7.2. Infrastructure Repair Index (IRI)

The IRI is the weighted geometric mean of the product of two components: 1. Product of the indicator variable if the LGU had infrastructure breakdowns during/ after the hydro-meteorological hazard, indicator variable if the damage was fixed and length of repair; and 2. Average of the product of the indicator variable if the LGU had infrastructure breakdowns during/ after the hydro-meteorological hazard and the agencies that funded the repair (Own city/municipality, DSWD, DILG, DOH, DepEd, DA, DPWH, AFP-OCD, BFP, Coast Guard, and PNP).

First, we calculate the index for each hydro-meteorological hazard (strong winds & rain, flood, landslide and big waves),

$$IRI_{i,s} = \sqrt{\frac{B_{i,s} \times F_{i,s} \times R_{i,s}}{7} \times \frac{B_{i,s} \times \sum_{j=1}^{11} A_{ij,s}}{11}} \times 100\%$$

then the weighted mean of the four hydro-meteorological hazards are calculated,

$$\overline{IRI_i} = \left(\frac{167}{189} IRI_{i,1} + \frac{20}{189} IRI_{i,2} + \frac{2}{189} IRI_{i,3} + \frac{0}{189} IRI_{i,4}\right) \times 100\%$$

where
$\overline{IRI_i}$ ≡ Infrastructure Repair Index of ith city/municipality
$IRI_{i,s}$ ≡ Infrastructure Repair Index of ith city/municipality for s^{th} hydro-meteorological hazard
$B_{i,s}$ ≡ Indicator variable if the ith city/municipality had infrastructure breakdowns during/after
the sth hydro-meteorological hazard (1 – Yes, 0 – No)
$F_{i,s}$ ≡ Indicator variable if the ith city/municipality fixed the infrastructure breakdown after the
the sth hydro-meteorological hazard (1 – Yes, 0 – No)
$R_{i,s}$ ≡ Ordinal variable of the length of repair in the ith city/municipality for the sth hydro-
meteorological hazard ≡ {Less than 1 day, 1 to 3 days, 4 days to 1 week, More than 1 week to 1
month, More than 1 month to 6 months, More than 6 months to 1 year, More than 1 year}
$A_{ij,s}$ ≡ Indicator variable for the agencies that funded the repair in the ith city/municipality after
the sth hydro-meteorological hazard (1 – Yes, 0 – No)
j ≡ type of agency ≡ {Own city/municipality, DSWD, DILG, DOH, DepEd, DA, DPWH, AFP-OCD,
BFP, Coast Guard, and PNP}
i ≡ city/municipality ≡ 1, 2, 3, ..., 189
s ≡ type of hydro-meteorological hazard ≡ {1- Strong winds & rain, 2 - Flood, 3 - Landslide, 4- Big waves}

8. **National Disaster Fund Index ($\overline{NDFI}$)**

The NDFI is the weighted geometric mean of two indices: 1.) National Disaster Fund Sources & Uses Index (NDFSUI); and 2.) National Disaster Fund Monetary Assistance Index (NDF-MAI).

First, we calculate the index for each hydro-meteorological hazard (strong winds & rain, flood, landslide and big waves),

$$NDFI_{i,s} = \sqrt{NDFSUI_{i,s} \times NDFMAI_{i,s}} \times 100\%$$

then the weighted mean of the four hydro-meteorological hazards are calculated,

$$\overline{NDFI_i} = \left(\frac{167}{189} NDFI_{i,1} + \frac{20}{189} NDFI_{i,2} + \frac{2}{189} NDFI_{i,3} + \frac{0}{189} NDFI_{i,4}\right) \times 100\%$$

where
$\overline{NDFI_i}$≡ Weighted National Disaster Fund Index of ith city/municipality
$NDFI_{i,s}$ ≡ National Disaster Fund Index of ith city/municipality for s^{th} hydro-meteorological hazard
$NDFSUI_{i,s}$ ≡ National Disaster Fund Sources & Uses Index of ith city/municipality for s^{th} hydro-

meteorological hazard
$NDFMAI_{i,s}$ ≡ National Disaster Fund Monetary Assistance Index of ith city/municipality for s^{th} hydro-meteorological hazard
i ≡ city/municipality ≡ 1, 2, 3, ..., 189
s ≡ type of hydro-meteorological hazard ≡ {1- Strong winds & rain, 2 - Flood, 3 - Landslide, 4 – Big waves}

9. Precautionary Measures Index - Long Term ($\overline{PMILT}$)

($\overline{PMILT}$) is the weighted mean of the product of type of long-term precautionary measures conducted by an LGU and its length of implementation. These measures include: Build resilient housing units, Invest In stronger public facilities, Build (cement) dams, dikes and river embankments, Upgrade power and water lines, Major road repairs, Identify relocation areas, Rezoning and land-use regulations, Build drainage, among others.

First, we calculate the index for each hydro-meteorological hazard (strong winds & rain, flood, landslide and big waves),

$$PMILT_{i,s} = \frac{\sum_{j=1}^{4} PMLT_{ij,s} \times LI_{ij,s}}{8 \times 4} \times 100\%$$

then the weighted mean of the four hydro-meteorological hazards are calculated:

$$\overline{PMILT_i} = \left(\frac{167}{189}PMILT_{i,1} + \frac{20}{189}PMILT_{i,2} + \frac{2}{189}PMILT_{i,3} + \frac{0}{189}PMILT_{i,4}\right) \times 100\%$$

where
$\overline{PMILT_i}$≡ Weighted Long Term Precautionary Measures Index of ith city/municipality
$PMILT_{i,s}$ ≡ Long Term Precautionary Measures Index of ith city/municipality for s^{th} type of hydro-meteorological hazard
$PMLT_{ij,s}$ ≡ Indicator variable for the type of long term precautionary measure conducted by i^{th} city/municipality for s^{th} hydro-meteorological hazard (1 – Yes, 0 – No)
LI_{ij} ≡ Ordinal variable for the length of implementation of jth type of long term precautionary measure conducted by i^{th} city/municipality for s^{th} type of hydro-meteorological hazard ≡ {Less than 1 year before disaster, 1 to 2 years before disaster, 2 to 3 years before disaster, More than 3 years before disaster}
i ≡ city/municipality ≡ 1, 2, 3, ..., 189
j ≡ types of long-term precautionary measure ≡ {Build resilent housing units, Invest in stronger public facilities, Build (Cement) dams, dikes and river embankments, Upgrade power and water lines, Major road repairs, Identify relocation areas, Rezoning and land-use regulations, Build drainage}
s ≡ type of hydro-meteorological hazard ≡ {1- Strong winds & rain, 2 - Flood, 3 - Landslide, 4 – Big waves}

10. Precautionary Measures Index - Mid-term ($\overline{PMIMT}$)

The $\overline{PMIMT}$ is the weighted mean of the product of type of mid-term precautionary measures and its frequency of implementation. These measures include: Assess the safety of public buildings, Strengthen river embankments and dikes using sandbags, Clean sewers and canals, Conduct road assessment and repairs, Repair/rehabilitate classrooms, etc.

First, we calculate the index for each hydro-meteorological hazard (strong winds & rain, flood, landslide and big waves),

$$PMIMT_{i,s} = \frac{\sum_{j=1}^{5} MTPM_{ij,s} \times FI_{ij,s}}{5 \times 5} \times 100\%$$

then the weighted average of the four hydro-meteorological hazards are calculated:

$$\overline{PMIMT_i} = \left(\frac{167}{189} MTPMI_{i,1} + \frac{20}{189} MTPMI_{i,2} + \frac{2}{189} MTPMI_{i,3} + \frac{0}{189} MTPMI_{i,4}\right) \times 100\%$$

where

$\overline{PMIMT_i}$≡ Weighted Mid − Term Precautionary Measures Index of ith city/municipality

$PMIMT_{i,s}$ ≡ Mid-term Precautionary Measures Index of ith city/municipality for s^{th} hydro-meteorological hazard

$PMMT_{ij,s}$ ≡ Indicator variable for the type of mid-term precautionary measure conducted by i^{th} city/municipality for s^{th} hydro-meteorological hazard (1 – Yes, 0 – No)

$FI_{ij,s}$ ≡ Ordinal variable for the frequency of implementation of the type of mid-term precautionary measure conducted by i^{th} city/municipality for s^{th} hydro-meteorological hazard ≡ {1 to 2 times since 2009/ 2012, 3 to 5 times since 2009/ 2012, 1 to 2 times a year, 3 to 5 times a year, 6 times or more a year}

i ≡ city/municipality ≡ 1, 2, 3, ..., 189

j ≡ types of mid-term precautionary measure ≡ {Assess safety of public buildings, Strengthen river embankments and dikes using sandbags, Clean sewers and canals, Conduct road assessment and repairs, Repair/rehabilitate classrooms}

s ≡ type of hydro-meteorological hazard ≡ {1- Strong winds & rain, 2 - Flood, 3 - Landslide, 4 – Big waves}

11. Precautionary Measures Index - Short Term ($\overline{PMIST}$)

The $\overline{PMIST}$ is the weighted geometric mean of the type of short-term precautionary measures, its time of implementation, and its length of implementation. These measures include the following: Class suspension, Gale warning, Road closures, etc.

First, we calculate the index for each hydro-meteorological hazard (strong winds & rain, flood, landslide and big waves). We use geometric mean to combine two sub-index components.

$$PMIST_{i,s} = \sqrt{\frac{\sum_{j=1}^{3} STPM_{ij,s} \times TI_{ij,s}}{3 \times 5} \times \frac{\sum_{j=1}^{3} STPM_{ij,s} \times LI_{ij,s}}{3 \times 4}} \times 100\%$$

then the weighted average of the four hydro-meteorological hazards are calculated

$$\overline{PMIST} = \left(\frac{167}{189} STPMI_{i,1} + \frac{20}{189} STPMI_{i,2} + \frac{2}{189} STPMI_{i,3} + \frac{0}{189} STPMI_{i,4}\right) \times 100\%$$

where

$\overline{PMIST_i}$≡ Weighted Short Term Precautionary Measures Index of ith city/municipality

$PMIST_{i,s}$ ≡ Short Term Precautionary Measures Index of ith city/municipality for s^{th} type of hydro-meteorological hazard using arithmetic mean

$PMST_{ij,s}$ ≡ Indicator variable for the type of short-term precautionary measure conducted by i^{th} city/municipality for s^{th} type of hydro-meteorological hazard (1 – Yes, 0 – No)

$TI_{ij,s}$ ≡ Ordinal variable for the time of implementation of jth type of mid-term precautionary measure

Ravago, Majah-Leah; Mapa, Claire Dennis; Sunglao, Jun Carlo; Aycardo, Angelie Grace (2020), "Data from a Survey of the Philippines' Local Governments on their Risk Management Strategies to Natural Disasters", Mendeley Data, V1, doi: 10.17632/9y2yhw45zt.1

conducted by i^{th} city/municipality for s^{th} type of hydro-meteorological hazard ≡ {More than 24
hours before disaster, 24 hours or less before disaster, During disaster, Less than 24 hours after
disaster, More than 24 hours after disaster}

$LI_{ij,s}$ ≡ Ordinal variable for the length of implementation of jth type of mid-term precautionary measure
conducted by i^{th} city/municipality for s^{th} type of hydro-meteorological hazard ≡ {Less than 1
day, 1 to 3 days, 4 days to 1 week, More than 1 week to 1 month}

i ≡ city/municipality ≡ 1, 2, 3, ..., 189

j ≡ types of short-term precautionary measure ≡ {Class suspension, Gale warning, Road closures}

s ≡ type of hydro-meteorological hazard ≡ {1- Strong winds & rain, 2 - Flood, 3 - Landslide, 4 – Big waves}

12. Quick Response Fund Index ($\overline{QRFI}$)

The QRFI is the weighted geometric mean of two indices: 1.) Quick Response Fund Uses Index (QRFUI); and 2.) Quick Response Fund Monetary Assistance Index (QRF-MAI).

First, we calculate the index for each hydro-meteorological hazard (strong winds & rain, flood, landslide and big waves),

$$QRFI_{i,s} = \sqrt{QRFUI_{i,s} \times QRFMAI_{i,s}} \times 100\%$$

then the weighted mean of the four hydro-meteorological hazards are calculated,

$$\overline{QRFI_i} = \left(\frac{167}{189}QRFI_{i,1} + \frac{20}{189}QRFI_{i,2} + \frac{2}{189}QRFI_{i,3} + \frac{0}{189}QRFI_{i,4}\right) \times 100\%$$

where

$\overline{QRFI_i}$≡ Weighted Quick Response Fund Index of ith city/municipality

$QRFI_{i,s}$ ≡ Quick Response Fund Index of ith city/municipality for s^{th} hydro-meteorological hazard

$QRFUI_{i,s}$ ≡ Quick Response Fund Uses Index of ith city/municipality for s^{th} hydro-meteorological hazard

$QRFMAI_{i,s}$ ≡ Quick Response Fund Monetary Assistance Index of ith city/municipality for s^{th} hydro-
meteorological hazard

i ≡ city/municipality ≡ 1, 2, 3, ..., 189

s ≡ type of hydro-meteorological hazard ≡ {1- Strong winds & rain, 2 - Flood, 3 – Landslide, 4 – Big waves}

12.1. Quick Response Fund Uses Index (QRFUI)

The QRFUI is the average of the product of the indicator variable if the LGU used its Quick Response Fund (QRF) and the types of fund use (Search and Rescue, Relief Goods Procurement, Soup Kitchen, Other Relief Operations, Clean-up Operations, Restoration of Lifeline Services, Employment and Livelihood, Housing and Relocation, Reconstruction of damaged buildings, Replacement and Repair of Lost Assets, and Monetary Assistance)

First, we calculate the index for each hydro-meteorological hazard (strong winds & rain, flood, landslide and big waves),

$$QRFUI_{i,s} = \frac{\sum_{j=1}^{11} U_{i,s} \times F_{ij,s}}{11} \times 100\%$$

then the weighted mean of the four hydro-meteorological hazards are calculated,

$$\overline{QRFUI_i} = \left(\frac{167}{189}QRFUI_{i,1} + \frac{20}{189}QRFUI_{i,2} + \frac{2}{189}QRFUI_{i,3} + \frac{0}{189}QRFUI_{i,4}\right) \times 100\%$$

where
$\overline{QRFUI_i}$ ≡ Weighted Quick Response Fund Uses Index of ith city/municipality
$QRFUI_{i,s}$ ≡ Quick Response Fund Uses Index of ith city/municipality for s^{th} hydro-meteorological hazard
$U_{i,s}$ ≡ Indicator variable if the ith city/municipality used its Quick Response Fund for the sth hydro-
meteorological hazard (1 – Yes, 0 – No)
$F_{ij,s}$ ≡ Indicator variable of the type of fund use (for QRF) of the ith city/municipality for s^{th} hydro-
meteorological hazard (1 – Yes, 0 – No)
j ≡ types of QRF fund use ≡ {Search and Rescue, Relief Goods Procurement, Soup Kitchen, Other Relief
Operations, Clean-up Operations, Restoration of Lifeline Services, Employment and Livelihood,
Housing and Relocation, Reconstruction of damaged buildings, Replacement and Repair of Lost
Assets, and Monetary Assistance}
i ≡ city/municipality ≡ 1, 2, 3, …, 189
s ≡ type of hydro-meteorological hazard ≡ {1- Strong winds & rain, 2 - Flood, 3 – Landslide, 4 – Big waves}

12.2. Quick Response Fund Monetary Assistance Index (QRF-MAI)

The QRF-MAI is the geometric average of two components: 1. Product of the indicator variable if the LGU used its Quick Response Fund (QRF) and indicator variable for QRF monetary assistance; and 2. Average of the product of the indicator variable if the LGU used its Quick Response Fund (QRF) and the types of monetary assistance given by the LGU (Emergency Shelter, Livelihood, Health and Unconditional)

First, we calculate the index for each hydro-meteorological hazard (strong winds & rain, flood, landslide and big waves),

$$QRF - MAI_{i,s} = \sqrt{\left(U_{i,s} \times M_{i,s}\right) \times \frac{\sum_{j=1}^{4} M_{i,s} \times T_{ij,s}}{4}} \times 100\%$$

then the weighted mean of the four hydro-meteorological hazards are calculated,

$$\overline{QRF - MAI_i} = \left(\frac{167}{189}QRF - MAI_{i,1} + \frac{20}{189}QRF - MAI_{i,2} + \frac{2}{189}QRF - MAI_{i,3} + \frac{0}{189}QRF - MAI_{i,4}\right) \times 100\%$$

where
$\overline{QRF - MAI_i}$≡ Weighted Quick Response Fund Monetary Assistance Index of ith city/municipality
$QRF - MAI_{i,s}$ ≡ Quick Response Fund Monetary Assistance Index of ith city/municipality for s^{th} hydro-meteorological hazard
$U_{i,s}$ ≡ Indicator variable if the ith city/municipality used its QRF for the sth hydro-meteorological hazard
(1 – Yes, 0 – No)
$M_{i,s}$ ≡ Indicator variable if the ith city/municipality used its QRF for monetary assistance for the sth hydro-
meteorological hazard (1 – Yes, 0 – No)

$T_{ij,s}$ ≡ Indicator variable of the type of monetary assistance given by the ith city/municipality for the sth
hydro-meteorological hazard
j ≡ types of monetary assistance ≡ {Emergency Shelter, Livelihood, Health and Unconditional}
i ≡ city/municipality ≡ 1, 2, 3, ..., 189
s ≡ type of hydro-meteorological hazard ≡ {1- Strong winds & rain, 2 - Flood, 3 - Landslide, 4 – Big waves}

13. Relief Index ($\overline{RI}$)

The $\overline{RI}$ is the weighted geometric mean of two indices: 1.) Relief Assistance Index (RAI); and 2.) Relief Goods Index (RGI).

First, we calculate the index for each hydro-meteorological hazard (strong winds & rain, flood, landslide and big waves),

$$RI_{i,s} = \sqrt{RAI_{i,s} \times RGI_{i,s}} \times 100\%$$

then the weighted mean of the four hydro-meteorological hazards are calculated,

$$\overline{RI_i} = \left(\frac{167}{189} RI_{i,1} + \frac{20}{189} RI_{i,2} + \frac{2}{189} RI_{i,3} + \frac{0}{189} RI_{i,4}\right) \times 100\%$$

where
$\overline{RI_i}$≡ Weighted Relief Index of ith city/municipality
$RI_{i,s}$ ≡ Relief Index of ith city/municipality for s^{th} hydro-meteorological hazard
$RAI_{i,s}$ ≡ Relief Assistance Index of ith city/municipality for s^{th} hydro-meteorological hazard
$RGI_{i,s}$ ≡ Relief Goods Index of ith city/municipality for s^{th} hydro-meteorological hazard
i ≡ city/municipality ≡ 1, 2, 3, ..., 189
s ≡ type of hydro-meteorological hazard ≡ {1- Strong winds & rain, 2 - Flood, 3 – Landslide, 4 – Big waves}

13.1. Relief Assistance Index (RAI)

The RAI is the geometric average of two components: 1. Average of the product of the indicator variable for presence of relief assistance to the constituents extended by the LGU and the type of relief assistance provided (Soup kitchen, Emergency shelter kit, Relief goods (e.g. food pack and water), and Medical kit); and 2. Average of the product of the indicator variable for presence of relief assistance to the constituents extended by the LGU and when it was provided.

First, we calculate the index for each hydro-meteorological hazard (strong winds & rain, flood, landslide and big waves),

$$RAI_{i,s} = \sqrt{\frac{\sum_{j=1}^{4} R_{i,s} \times A_{ij,s}}{4} \times \frac{\sum_{j=1}^{4} R_{i,s} \times S_{ij,s}}{4 \times 6}} \times 100\%$$

then the weighted mean of the four hydro-meteorological hazards are calculated,

$$\overline{RAI_i} = \left(\frac{167}{189} RAI_{i,1} + \frac{20}{189} RAI_{i,2} + \frac{2}{189} RAI_{i,3} + \frac{0}{189} RAI_{i,4}\right) \times 100\%$$

where
$\overline{RAI_i}$≡ Weighted Relief Assistance Index of ith city/municipality
$RAI_{i,s}$ ≡ Relief Assistance Index of ith city/municipality for s^{th} hydro-meteorological hazard
$R_{i,s}$ ≡ Indicator variable if the ith city/municipality extended assistance/ relief to its constituents for the sth
hydro-meteorological hazard (1 – Yes, 0 – No)
$A_{ij,s}$ ≡ Indicator variable for the type of assistance extended by the ith city/ municipality for the sth hydro-
meteorological hazard (1 – Yes, 0 – No)

$S_{ij,s}$ ≡ Ordinal variable for the start of relief by the ith city/municipality for the sth hydro-meteorological
hazard ≡ {1, 2, 3, …, *6}*
j ≡ types of assistance/ relief ≡ { Soup kitchen, Emergency shelter kit, Relief goods (e.g. food pack and
water), Medical kit}
i ≡ city/municipality ≡ 1, 2, 3, …, 189
s ≡ type of hydro-meteorological hazard ≡ {1- Strong winds & rain, 2 - Flood, 3 – Landslide, 4 – Big waves}

13.2. Relief Goods Index (RGI)

The RGI is the geometric average of two components: 1. Product of the indicator variable for presence of relief assistance to the constituents extended by the LGU and indicator variable for relief goods assistance; and 2. Product of the indicator variable for relief goods assistance and duration of relief provided.

First, we calculate the index for each hydro-meteorological hazard (strong winds & rain, flood, landslide and big waves),

$$RGI_{i,s} = \sqrt{\left(R_{i,s} \times G_{i,s}\right) \times \frac{G_{i,s} \times D_{i,s}}{7}} \times 100\%$$

then the weighted mean of the four hydro-meteorological hazards are calculated,

$$\overline{RGI_i} = \left(\frac{167}{189} RGI_{i,1} + \frac{20}{189} RGI_{i,2} + \frac{2}{189} RGI_{i,3} + \frac{0}{189} RGI_{i,4}\right) \times 100\%$$

where
$\overline{RGI_i}$≡ Weighted Relief Goods Index of ith city/municipality
$RGI_{i,s}$ ≡ Relief Goods Index of ith city/municipality for s^{th} hydro-meteorological hazard
$R_{i,s}$ ≡ Indicator variable if the ith city/municipality extended assistance/ relief to its constituents for the sth
hydro-meteorological hazard (1 – Yes, 0 – No)
$G_{i,s}$ ≡ Indicator variable if the ith city/ municipality provided relief goods to the constituents for the sth
hydro-meteorological hazard (1 – Yes, 0 – No)
$D_{i,s}$ ≡ Duration of relief goods provided by the ith city/municipality for the sth hydro-meteorological
hazard ≡ {Less than 1 day, 1 to 3 days, 4 days to 1 week, More than 1 week to 1 month, More than
1 month to 6 months, More than 6 months to 1 year, More than 1 year}
i ≡ city/municipality ≡ 1, 2, 3, …, 189
s ≡ type of hydro-meteorological hazard ≡ {1- Strong winds & rain, 2 - Flood, 3 - Landslide, 4 – Big waves}

14. Response & Assistance from Others Index ($\overline{RAOI}$)

The $\overline{RAOI}$ is the weighted geometric mean of two indices: 1. Response from Others Index (ROI); and 2. Assistance from Others Index (AOI).

First, we calculate the index for each hydro-meteorological hazard (strong winds & rain, flood, landslide and big waves),

$$RAOI_{i,s} = \sqrt{ROI_{i,s} \times AOI_{i,s}} \times 100\%$$

then the weighted mean of the four hydro-meteorological hazards are calculated,

$$\overline{RAOI_i} = \left(\frac{167}{189} RAOI_{i,1} + \frac{20}{189} RAOI_{i,2} + \frac{2}{189} RAOI_{i,3} + \frac{0}{189} RAOI_{i,4}\right) \times 100\%$$

where

$\overline{RAOI_i}$ ≡ Response & Assistance from Others Index of ith city/municipality
$RAOI_{i,s}$ ≡ Response & Assistance from Others Index of ith city/municipality for s^{th} hydro-meteorological
hazard
$ROI_{i,s}$ ≡ Response from Others Index of ith city/municipality for s^{th} hydro-meteorological hazard
$AOI_{i,s}$ ≡ Assistance from Others Index of ith city/municipality for s^{th} hydro-meteorological hazard
i ≡ city/municipality ≡ 1, 2, 3, ..., 189
s ≡ type of hydro-meteorological hazard ≡ {1- Strong winds & rain, 2 - Flood, 3 - Landslide, 4 – Big waves}

14.1. Response from Others Index (ROI)

The ROI is the average of the product of the indicator variable for presence of assistance extended by other government agencies, LGUs or NGOs after the hydro-meteorological hazard and the agencies that provided assistance (DSWD, DILG, DOH, DepEd, DA, DPWH, AFP-OCD, BFP, Coast Guard, PNP, Other agency, Other city, Other province, Local NGOs and Foreign NGOs)

First, we calculate the index for each hydro-meteorological hazard (strong winds & rain, flood, landslide and big waves),

$$ROI_{i,s} = \frac{\sum_{j=1}^{10} A_{i,s} \times O_{ij,s}}{10} \times 100\%$$

then the weighted mean of the four hydro-meteorological hazards are calculated,

$$\overline{ROI_i} = \left(\frac{167}{189} ROI_{i,1} + \frac{20}{189} ROI_{i,2} + \frac{2}{189} ROI_{i,3} + \frac{0}{189} ROI_{i,4}\right) \times 100\%$$

where
$\overline{ROI_i}$≡ Response from Others Index of ith city/municipality
$ROI_{i,s}$ ≡ Response from Others Index of ith city/municipality for s^{th} hydro-meteorological hazard
$A_{i,s}$ ≡ Indicator variable if other government agencies, LGUs or NGOs extended assistance to the ith
city/municipality after the sth hydro-meteorological hazard (1 – Yes, 0 – No)
$O_{ij,s}$ ≡ Indicator variable for the type of agency that assisted the ith city/ municipality for the sth hydro-
meteorological hazard (1 – Yes, 0 – No)
j ≡ type of agency ≡ {DSWD, DILG, DOH, DepEd, DA, DPWH, AFP-OCD, BFP, Coast Guard, PNP}
i ≡ city/municipality ≡ 1, 2, 3, ..., 189
s ≡ type of hydro-meteorological hazard ≡ {1- Strong winds & rain, 2 - Flood, 3 - Landslide, 4- Big waves}

14.2. Assistance from Others Index (AOI)

The AOI is the weighted geometric mean of two components: 1. Average of the product of the indicator variable for presence of assistance extended by other government agencies, LGUs or NGOs after the hydro-meteorological hazard and the types of assistance; and 2. the product of the types of assistance (Search and Rescue, Relief Goods, Soup Kitchen, Other Relief Operations, Clean-up Operations, Restoration of Lifeline Services, Employment and Livelihood, Housing and Relocation, Reconstruction of damaged buildings, Replacement and Repair of Lost Assets, and Monetary Assistance) and duration of assistance provided.

First, we calculate the index for each hydro-meteorological hazard (strong winds & rain, flood, landslide and big waves),

$$AOI_{i,s} = \sqrt{\frac{\sum_{j=1}^{11} A_{i,s} \times T_{ij,s}}{11} \times \frac{T_{ij,s} \times D_{ij,s}}{7}} \times 100\%$$

then the weighted mean of the four hydro-meteorological hazards are calculated,

$$\overline{AOI_i} = \left(\frac{167}{189} AOI_{i,1} + \frac{20}{189} AOI_{i,2} + \frac{2}{189} AOI_{i,3} + \frac{0}{189} AOI_{i,4}\right) \times 100\%$$

where

$\overline{AOI_i}$ ≡ Assistance from Others Index of ith city/municipality

$AOI_{i,s}$ ≡ Assistance from Others Index of ith city/municipality for s^{th} hydro-meteorological hazard

$A_{i,s}$ ≡ Indicator variable if other government agencies, LGUs or NGOs extended assistance to the ith city/municipality after the sth hydro-meteorological hazard (1 – Yes, 0 – No)

$T_{ij,s}$ ≡ Indicator variable for the type of assistance provided to the ith city/ municipality for the sth hydro-meteorological hazard (1 – Yes, 0 – No)

$D_{ij,s}$ ≡ Ordinal variable for the duration of assistance provided to the ith city/ municipality for the sth hydro-meteorological hazard ≡ {1, 2, 3, ..., 7}

j ≡ type of assistance ≡ {Search and Rescue, Relief Goods, Soup Kitchen, Other Relief Operations, Clean-up Operations, Restoration of Lifeline Services, Employment and Livelihood, Housing and Relocation, Reconstruction of damaged buildings, Replacement and Repair of Lost Assets, and Monetary Assistance}

i ≡ city/municipality ≡ 1, 2, 3, ..., 189

s ≡ type of hydro-meteorological hazard ≡ {1- Strong winds & rain, 2 - Flood, 3 - Landslide, 4- Big waves}

15. Search & Rescue Index ($\overline{SRI}$)

The $\overline{SRI}$ is the weighted arithmetic mean of the product of the indicator variable if the LGU conducted search & rescue and the ordinal variable for no. of people rescued (Less than 100 people, 101 to 200 people, 201 to 300 people, 301 to 400 people, 401 to 500 people, More than 501 people)

First, we calculate the index for each hydro-meteorological hazard (strong winds & rain, flood, landslide and big waves),

$$SRI_{i,s} = \frac{SR_{i,s} \times P_{ij,s}}{6} \times 100\%$$

then the weighted mean of the four hydro-meteorological hazards are calculated,

$$\overline{SRI_i} = \left(\frac{167}{189} SRI_{i,1} + \frac{20}{189} SRI_{i,2} + \frac{2}{189} SRI_{i,3} + \frac{0}{189} SRI_{i,4}\right) \times 100\%$$

where

$\overline{SRI_i}$≡ Weighted Search & Rescue Index of ith city/municipality

$SRI_{i,s}$ ≡ Search & Rescue Index of ith city/municipality for s^{th} hydro-meteorological hazard

$SR_{i,s}$ ≡ Indicator variable if the ith city/municipality conducted search & rescue for the sth hydro-meteorological hazard (1 – Yes, 0 – No)

$P_{ij,s}$ ≡ Ordinal variable for the no. of people rescued by the ith city/municipality for s^{th} hydro-meteorological hazard

j ≡ no. of people rescued (no. of intervals)≡ {Less than 100 people, 101 to 200 people, 201 to 300 people,

301 to 400 people, 401 to 500 people, More than 501 people}
i ≡ city/municipality ≡ 1, 2, 3, ..., 189
s ≡ type of hydro-meteorological hazard ≡ {1- Strong winds & rain, 2 - Flood, 3 – Landslide, 4 – Big waves}

15.1. National Disaster Fund Sources & Uses Index (NDF-SUI)

The NDF-SUI is the geometric average of two components: 1.) Average of the product of the indicator variable if the LGU availed the NDF for the hydro-meteorological hazard and the indicator variable for the agency that released the fund; and 2.) Average of the product of the indicator variable if the LGU availed the NDF for the hydro-meteorological hazard and the indicator variable if the LGU availed the NDF for the hydro-meteorological hazard and the uses of the fund (Search and Rescue, Relief Goods Procurement, Soup Kitchen, Other Relief Operations, Clean-up Operations, Restoration of Lifeline Services, Employment and Livelihood, Housing & Relocation, Reconstruction of damaged buildings, Replacement & Repair of Lost Assets, Monetary Assistance, etc.).

First, we calculate the index for each hydro-meteorological hazard (strong winds & rain, flood, landslide and big waves),

$$NDF - SUI_{i,s} = \sqrt{\frac{\sum_{j=1}^{10} F_{i,s} \times A_{ij,s}}{10} + \frac{\sum_{k=1}^{11} F_{ik,s} \times U_{ik,s}}{11}} \times 100\%$$

then the weighted mean of the four hydro-meteorological hazards are calculated,

$$\overline{NDF - SUI_i} = \left(\frac{167}{189} NDF - SUI_{i,1} + \frac{20}{189} NDF - SUI_{i,2} + \frac{2}{189} NDF - SUI_{i,3} + \frac{0}{189} NDF - SUI_{i,4}\right) \times 100\%$$

where
$\overline{NDF - SUI_i}$≡ Weighted National Disaster Fund Sources & Uses Index of ith city/municipality
$NDF - SUI_{i,s}$ ≡ National Disaster Fund Sources & Uses Index of ith city/municipality for s^{th} hydro-meteorological hazard
$F_{i,s}$ ≡ Indicator variable if the ith city/municipality availed the NDF for the sth hydro-meteorological hazard
(1 – Yes, 0 – No)
$A_{ij,s}$ ≡ Indicator variable for the agency that released the NDF fund for the ith city/ municipality for the
s^{th} hydro-meteorological hazard (1 – Yes, 0 – No)
$U_{ik,s}$ ≡ Indicator variable for the type of fund use by the ith city/municipality for the sth hydro-meteorological hazard (1 – Yes, 0 – No)
j ≡ types of agencies ≡ {DSWD, DILG, DOH, DepEd, DA, DPWH, AFP-OCD, BFP, Coast Guard, PNP}
k ≡ types of fund use ≡ {Search and Rescue, Relief Goods Procurement, Soup Kitchen, Other Relief
Operations, Clean-up Operations, Restoration of Lifeline Services, Employment and Livelihood,
Housing & Relocation, Reconstruction of damaged buildings, Replacement & Repair of Lost
Assets, Monetary Assistance}
i ≡ city/municipality ≡ 1, 2, 3, ..., 189
s ≡ type of hydro-meteorological hazard ≡ {1- Strong winds & rain, 2 - Flood, 3 – Landslide, 4 – Big waves}

15.2. National Disaster Fund Monetary Assistance Index (NDF-MAI)

The NDF-MAI the geometric average of two components: 1. Product of the indicator variable if the LGU availed funding from the National Disaster Fund/ Calamity Fund and the indicator variable if the fund was used for monetary assistance; and 2. Average of the product of the indicator variable if the fund was used for monetary assistance and types of monetary assistance offered (Emergency shelter, livelihood, health, unconditional, etc).

First, we calculate the index for each hydro-meteorological hazard (strong winds & rain, flood, landslide and big waves),

$$NDF - MAI_{i,s} = \sqrt{\left(F_{i,s} \times M_{i,s}\right) + \frac{\sum_{j=1}^{4} M_{i,s} \times U_{ij,s}}{4}} \times 100\%$$

then the weighted mean of the four hydro-meteorological hazards are calculated,

$$\overline{NDF - MAI_i} = \left(\frac{167}{189} NDF - MAI_{i,1} + \frac{20}{189} NDF - MAI_{i,2} + \frac{2}{189} NDF - MAI_{i,3} + \frac{0}{189} NDF - MAI_{i,4}\right) \times 100\%$$

where

$\overline{NDF - MAI_i}$≡ Weighted National Disaster Fund Monetary Assistance Index of ith city/municipality

$NDF - MAI_{i,s}$ ≡ National Disaster Fund Monetary Assistance Index of ith city/municipality for s^{th} hydro-meteorological hazard

$F_{i,s}$ ≡ Indicator variable if the ith city/municipality availed the NDF for the sth hydro-meteorological hazard (1 – Yes, 0 – No)

$M_{i,s}$ ≡ Indicator variable if the ith city/ municipality used the fund for monetary assistance for the sth hydro-meteorological hazard (1 – Yes, 0 – No)

$U_{ij,s}$ ≡ Indicator variable for the type of fund use by the ith city/municipality for the sth hydro-meteorological hazard (1 – Yes, 0 – No)

j ≡ types of fund use ≡ {Emergency shelter, livelihood, health, unconditional}

i ≡ city/municipality ≡ 1, 2, 3, …, 189

s ≡ type of hydro-meteorological hazard ≡ {1- Strong winds & rain, 2 - Flood, 3 – Landslide, 4 – Big waves}

16. Service Interruption Index-Type ($\overline{SIT}$)

The $\overline{SIT}$ is the weighted geometric mean of three indices: 1.) Water Supply Interruption Index (WSII), 2.) Telecommunication Interruption Index (TII), and 3.) Electricity Interruption Index (EII).

First, we calculate the index for each hydro-meteorological hazard (strong winds & rain, flood, landslide and big waves),

$$SIT_{i,s} = \sqrt[3]{WSII_{i,s} \times TII_{i,s} \times EII_{i,s}} \times 100\%$$

then the weighted mean of the four hydro-meteorological hazards are calculated,

$$\overline{SIT_i} = \left(\frac{167}{189} SIT_{i,1} + \frac{20}{189} SIT_{i,2} + \frac{2}{189} SIT_{i,3} + \frac{0}{189} SIT_{i,4}\right) \times 100\%$$

where

$\overline{SIT_i}$ ≡ Type of Service Interruption Index of ith city/municipality

$SIT_{i,s}$ ≡ Type of Service Interruption Index of ith city/municipality for s^{th} hydro-meteorological hazard

$WSII_{i,s}$ ≡ Water Supply Interruption Index of ith city/municipality for s^{th} hydro-meteorological hazard

$TII_{i,s}$ ≡ Telecommunication Interruption Index of ith city/municipality for s^{th} hydro-meteorological hazard
$EII_{i,s}$ ≡ Electricity Interruption Index of ith city/municipality for s^{th} hydro-meteorological hazard
i ≡ city/municipality ≡ 1, 2, 3, ..., 189
s ≡ type of hydro-meteorological hazard ≡ {1- Strong winds & rain, 2 - Flood, 3 - Landslide, 4- Big waves}

16.1. Water Supply Interruption Index (WSII)

The WSII is the average of two components: 1. Product of the indicator variable if the LGU had water supply interruption during the hydro-meteorological hazard and when the water supply was cut-off; 2. Average of the product of the indicator variable if the LGU had water supply interruption during the hydro-meteorological hazard, length of interruption and stop-gap measures utilized (Rationing, Water Wells and Mobile Water Treatment).

First, we calculate the index for each hydro-meteorological hazard (strong winds & rain, flood, landslide and big waves),

$$WSII_{i,s} = \sqrt{\frac{W_{i,s} \times C_{i,s}}{5} \times \frac{W_{i,s} \times L_{i,s} \times \sum_{j=1}^{3} S_{ij,s}}{3 \times 7}} \times 100\%$$

then the weighted mean of the four hydro-meteorological hazards are calculated,

$$\overline{WSII_i} = \left(\frac{167}{189} WSII_{i,1} + \frac{20}{189} WSII_{i,2} + \frac{2}{189} WSII_{i,3} + \frac{0}{189} WSII_{i,4}\right) \times 100\%$$

where
$\overline{WSII_i}$ ≡ Water Supply Interruption Index of ith city/municipality
$WSII_{i,s}$ ≡ Water Supply Interruption Index of ith city/municipality for s^{th} hydro-meteorological hazard
$W_{i,s}$ ≡ Indicator variable if the ith city/municipality had water service interruption during the sth
hydro-meteorological hazard (1 – Yes, 0 – No)
$C_{i,s}$ ≡ Ordinal variable for when the water supply was cut-off in the ith city/municipality during the sth
hydro-meteorological hazard ≡ {1, 2, 3, ..., *5*}
$L_{i,s}$ ≡ Ordinal variable for the length of water supply interruption in the ith city/municipality during the sth
hydro-meteorological hazard ≡ {1, 2, 3, ...,7}
$S_{ij,s}$ ≡ Indicator variable of the stop-gap measures utilized by the ith city/municipality during the sth hydro-
meteorological hazard (1 – Yes, 0 – No)
j ≡ stop-gap measures ≡ {Rationing, Water Wells and Mobile Water Treatment}
i ≡ city/municipality ≡ 1, 2, 3, ..., 189
s ≡ type of hydro-meteorological hazard ≡ {1- Strong winds & rain, 2 - Flood, 3 - Landslide, 4- Big waves}

16.2. Telecommunication Interruption Index (TII)

The TII is the weighted geometric mean of two components: 1. Product of the indicator variable if the LGU had telecommunication interruption during the hydro-meteorological hazard and when the telecommunication service was cut-off; and 2. Average of the product of the indicator variable if the LGU had telecommunication interruption during the hydro-meteorological hazard, length of interruption and stop-gap measures utilized (Satellite phone and Two-way radio).

First, we calculate the index for each hydro-meteorological hazard (strong winds & rain, flood, landslide and big waves),

$$TII_{i,s} = \sqrt{\frac{T_{i,s} \times C_{i,s}}{5} \times \frac{T_{i,s} \times L_{i,s} \times \sum_{j=1}^{2} S_{ij,s}}{2 \times 7}} \times 100\%$$

then the weighted mean of the four hydro-meteorological hazards are calculated,

$$\overline{TII_i} = \left(\frac{167}{189} TII_{i,1} + \frac{20}{189} TII_{i,2} + \frac{2}{189} TII_{i,3} + \frac{0}{189} TII_{i,4}\right) \times 100\%$$

where

$\overline{TII_i}$ ≡ Telecommunication Interruption Index of ith city/municipality

$TII_{i,s}$ ≡ Telecommunication Interruption Index of ith city/municipality for s^{th} hydro-meteorological hazard

$T_{i,s}$ ≡ Indicator variable if the ith city/municipality had telecommunication interruption during the sth

hydro-meteorological hazard (1 – Yes, 0 – No)

$C_{i,s}$ ≡ Ordinal variable for when the telecommunication service was cut-off in the ith city/municipality

during the sth hydro-meteorological hazard ≡ {1, 2, , …, *5}*

$L_{i,s}$ ≡ Ordinal variable for the length of telecommunication interruption in the ith city/municipality during

the sth hydro-meteorological hazard ≡ {1, 2, 3, …, *7}*

$S_{ij,s}$ ≡ Indicator variable of the stop-gap measures utilized by the ith city/municipality during the sth hydro-

meteorological hazard (1 – Yes, 0 – No)

j ≡ stop-gap measures ≡ {Satellite phone and Two-way radio}

i ≡ city/municipality ≡ 1, 2, 3, …, 189

s ≡ type of hydro-meteorological hazard ≡ {1- Strong winds & rain, 2 - Flood, 3 - Landslide, 4- Big waves}

16.3. Electricity Interruption Index (EII)

The EII is the weighted geometric mean of two components: 1. Product of the indicator variable if the LGU had electricity interruption during the hydro-meteorological hazard, when the electricity service was cut-off; and 2. Average of the indicator variable if the LGU had electricity interruption during the hydro-meteorological hazard, length of interruption and stop-gap measures utilized (Gas or Diesel-powered generators and Solar panels).

First, we calculate the index for each hydro-meteorological hazard (strong winds & rain, flood, landslide and big waves),

$$EII_{i,s} = \sqrt{\frac{E_{i,s} \times C_{i,s}}{5} \times \frac{E_{i,s} \times L_{i,s} \times \sum_{j=1}^{2} S_{ij,s}}{2 \times 7}} \times 100\%$$

then the weighted mean of the four hydro-meteorological hazards are calculated,

$$\overline{EII_i} = \left(\frac{167}{189} EII_{i,1} + \frac{20}{189} EII_{i,2} + \frac{2}{189} EII_{i,3} + \frac{0}{189} EII_{i,4}\right) \times 100\%$$

where

$\overline{EII_i}$ ≡ Electricity Interruption Index of ith city/municipality

$EII_{i,s}$ ≡ Electricity Interruption Index of ith city/municipality for s^{th} hydro-meteorological hazard

$E_{i,s}$ ≡ Indicator variable if the ith city/municipality had electricity interruption during the sth hydro-

meteorological hazard (1 – Yes, 0 – No)

$C_{i,s}$ ≡ Ordinal variable for when the electricity service was cut-off in the ith city/municipality during the

s^{th} hydro-meteorological hazard ≡ {1, 2, 3, ..., *5*}
$L_{i,s}$ ≡ Ordinal variable for the length of electricity interruption in the ith city/municipality during the sth
hydro-meteorological hazard ≡ {1, 2, 3, ..., *7*}
$S_{ij,s}$ ≡ Indicator variable of the stop-gap measures utilized by the ith city/municipality during the sth hydro-
meteorological hazard (1 – Yes, 0 – No)
j ≡ stop-gap measures ≡ {Gas or Diesel-powered generators and Solar panels}
i ≡ city/municipality ≡ 1, 2, 3, ..., 189
s ≡ type of hydro-meteorological hazard ≡ {1- Strong winds & rain, 2 - Flood, 3 - Landslide, 4- Big waves}

17. Service Interruption Index ($\overline{SII}$)

The $\overline{SII}$ is the weighted average of the product of the indicator variable if the LGU had any service interruption during the hydro-meteorological hazard and the types of service interruption (Water, Telecommunication and Electricity).

First, we calculate the index for each hydro-meteorological hazard (strong winds & rain, flood, landslide and big waves),

$$SII_{i,s} = \frac{\sum_{j=1}^{3} S_{i,s} \times T_{ij,s}}{3} \times 100\%$$

then the weighted mean of the four hydro-meteorological hazards are calculated,

$$\overline{SII_i} = \left(\frac{167}{189} SII_{i,1} + \frac{20}{189} SII_{i,2} + \frac{2}{189} SII_{i,3} + \frac{0}{189} SII_{i,4}\right) \times 100\%$$

where
$\overline{SII_i}$≡ Timely Delivery of Service Index of ith city/municipality
$SII_{i,s}$ ≡ Timely Delivery of Service Index of ith city/municipality for s^{th} hydro-meteorological hazard
$S_{i,s}$ ≡ Indicator variable if the ith city/municipality had any service interruption during the sth
hydro-meteorological hazard (1 – Yes, 0 – No)
$T_{ij,s}$ ≡ Indicator variable for the type of service interruptions experienced by the ith city/
municipality during the sth hydro-meteorological hazard ≡ (1 – Yes, 0 – No)
j ≡ types of service interruption ≡ {Water, Telecommunication and Electricity}
i ≡ city/municipality ≡ 1, 2, 3, ..., 189
s ≡ type of hydro-meteorological hazard ≡ {1- Strong winds & rain, 2 - Flood, 3 - Landslide, 4- Big waves}

18. Trainings Index ($\overline{TI}$)

The TI is the geometric average of two indices: 1.) Trainings Given Index (TGI), and 2.) Trainings Received Index (TRI).

$$TI_i = \sqrt{TGI_i \times TGI_i} \times 100\%$$

where
TGI_i ≡ Trainings Given Index of ith city/municipality
TRI_i ≡ Trainings Received Index of ith city/municipality
i ≡ city/municipality ≡ 1, 2, 3, ..., 189

18.1. Trainings Received Index (TRI)

The TRI is the average of the type of training received by different individuals/ institutions and the type of training received by the municipality. The type of trainings are as follows: Prevention and Mitigation, Information and Awareness, Evacuation, Early Warning, Search and Rescue, Relief and Recovery.

$$TRI_i = \left(\sum_{j=1}^{8} \frac{B_{ij} + L_{ij} + D_{ij} + OL_{ij} + ON_{ij} + O_{ij}}{6}\right) \times \frac{T_j}{8} \times 100\%$$

where

TRI_i ≡ Trainings Received Index of ith city/municipality

B_{ij} ≡ Indicator variable if the ith city/municipality received trainings for Barangay Officials for the jth training type (1 – Yes, 0 – No)

L_{ij} ≡ Indicator variable if the ith city/municipality received trainings for Local Constituents for the jth training type (1 – Yes, 0 – No)

D_{ij} ≡ Indicator variable if the ith city/municipality received trainings for Disaster Volunteers for the jth training type (1 – Yes, 0 – No)

OL_{ij} ≡ Indicator variable if the ith city/municipality received trainings for other LGUs for the jth training type (1 – Yes, 0 – No)

ON_{ij} ≡ Indicator variable if the ith city/municipality received trainings for other NGOs for the jth training type (1 – Yes, 0 – No)

O_{ij} ≡ Indicator variable if the ith city/municipality received trainings for Others for the jth training type (1 – Yes, 0 – No)

T_j ≡ Indicator variable if the ith city/municipality conducted j^{th} training type (1 – Yes, 0 – No)

i ≡ city/municipality ≡ 1, 2, 3, ..., 189

j ≡ type of training ≡ {Prevention and Mitigation, Information and Awareness, Evacuation, Early Warning, Search and Rescue, Relief, Recovery}

18.2. Trainings Given Index (TGI)

The TGI is the average of the type of training given to different individuals/ institutions and the type of training conducted by the municipality. The type of trainings are as follows: Prevention and Mitigation, Information and Awareness, Evacuation, Early Warning, Search and Rescue, Relief and Recovery.

$$TGI_i = \left(\sum_{j=1}^{8} \frac{B_{ij} + L_{ij} + D_{ij} + OL_{ij} + ON_{ij} + O_{ij}}{6}\right) \times \frac{T_j}{8} \times 100\%$$

where

TGI_i ≡ Trainings Given Index of ith city/municipality

B_{ij} ≡ Indicator variable if the ith city/municipality gave trainings to Barangay Officials for the jth training type (1 – Yes, 0 – No)

L_{ij} ≡ Indicator variable if the ith city/municipality gave trainings to Local Constituents for the jth training type (1 – Yes, 0 – No)

D_{ij} ≡ Indicator variable if the ith city/municipality gave trainings to Disaster Volunteers for the jth training type (1 – Yes, 0 – No)

OL_{ij} ≡ Indicator variable if the ith city/municipality gave trainings to other LGUs for the jth training type (1 – Yes, 0 – No)

ON_{ij} ≡ Indicator variable if the ith city/municipality gave trainings to other NGOs for the jth training type (1 – Yes, 0 – No)

O_{ij} ≡ Indicator variable if the ith city/municipality gave trainings to Others for the jth training type (1 – Yes, 0 – No)

T_j ≡ Indicator variable if the ith city/municipality conducted j^{th} training type (1 – Yes, 0 – No)

i ≡ city/municipality ≡ 1, 2, 3, ..., 189

j ≡ type of training ≡ {The type of trainings are as follows: Prevention and Mitigation, Information and
Awareness, Evacuation, Early Warning, Search and Rescue, Relief, Recovery}

19. Warning Index ($\overline{WI}$)

The $\overline{WI}$ is the weighted geometric mean of three indices: 1.) Source of Warnings Index (SWI), 2.) Preparatory Checks Index (PCI) and 3.) Warning Issued Index (WII).
First, we calculate the mean of the index for each hydro-meteorological hazard (strong winds & rain, flood, landslide and big waves) using geometric mean.

$$WI_{i,s} = \left(\sqrt[3]{SWI_{i,s} \times PCI_{i,s} \times WII_{i,s}}\right) \times 100\%$$

then the weighted means of the four hydro-meteorological hazards are calculated,

$$\overline{WI_i} = \left(\frac{167}{189}WI_{i,1} + \frac{20}{189}WI_{i,2} + \frac{2}{189}WI_{i,3} + \frac{0}{189}WI_{i,4}\right) \times 100\%$$

where
$\overline{WI_i}$≡ Weighted Warning Index of ith city/municipality using arithmetic mean
$WI_{i,s}$ ≡ Warning Index of ith city/municipality for s^{th} hydro-meteorological hazard
$SWI_{i,s}$ ≡ Source of Warnings Index of ith city/municipality for s^{th} hydro-meteorological hazard
$PCI_{i,s}$ ≡ Preparatory Checks Index of ith city/municipality for s^{th} hydro-meteorological hazard
$WII_{i,s}$ ≡ Warning Issued Index of ith city/municipality for s^{th} hydro-meteorological hazard
i ≡ city/municipality ≡ 1, 2, 3, ..., 189
s ≡ type of hydro-meteorological hazard ≡ {1- Strong winds & rain, 2 - Flood, 3 - Landslide, 4 – Big waves}

19.1. Source of Warnings Index (SWI)

The SWI is the average of the product of presence of received warning before the hydro-meteorological hazard occurred and the sources of warning, which includes: PAGASA/DOST, Provincial DRRMO, NDRRMC, Local media, Other government agency, etc.

First, we calculate the index for each hydro-meteorological hazard (strong winds & rain, flood, landslide and big waves),

$$SWI_{i,s} = \frac{\sum_{j=1}^{4} R_{i,s} \times W_{ij,s}}{4} \times 100\%$$

then the weighted mean of the four hydro-meteorological hazards are calculated,

$$\overline{SWI_i} = \left(\frac{167}{189}SWI_{i,1} + \frac{20}{189}SWI_{i,2} + \frac{2}{189}SWI_{i,3} + \frac{0}{189}SWI_{i,4}\right) \times 100\%$$

where
$\overline{SWI_i}$≡ Weighted Source of Warnings Index of ith city/municipality
$SWI_{i,s}$ ≡ Source of Warnings Index of ith city/municipality for s^{th} hydro-meteorological hazard
$R_{i,s}$ ≡ Indicator variable if the ith city/municipality receive/ hear a warning before the hydro-meteorological
hazard occurred for s^{th} hydro-meteorological hazard (1 – Yes, 0 – No)
$W_{ij,s}$ ≡ Indicator variable of the jth source of warning received by i^{th} city/municipality for s^{th} hydro-
meteorological hazard (1 – Yes, 0 – No)
j ≡ warning sources ≡ {PAGASA/DOST, Provincial DRRMO, NDRRMC, Local media, Other government
agency, Other NGO}
i ≡ city/municipality ≡ 1, 2, 3, ..., 189
s ≡ type of hydro-meteorological hazard ≡ {1- Strong winds & rain, 2 - Flood, 3 - Landslide, 4 – Big waves}

19.2. Preparatory Checks Index (PCI)

The PCI is the average of the product of presence of preparatory checks conducted after receiving / hearing the warning and the kinds of preparatory checks conducted, which are the following: Check inventory of supplies and equipment, Check capacity of critical facilities like hospitals, Organize DRRM teams and personnel, Enlist volunteers, Prepare evacuation centers, and Prepare and preposition relief goods.

First, we calculate the index for each hydro-meteorological hazard (strong winds & rain, flood, landslide and big waves),

$$PCI_{i,s} = \frac{\sum_{j=1}^{6} C_{i,s} \times PC_{ij,s}}{6} \times 100\%$$

then the weighted mean of the four hydro-meteorological hazards are calculated,

$$\overline{PCI_i} = \left(\frac{167}{189} PCI_{i,1} + \frac{20}{189} PCI_{i,2} + \frac{2}{189} PCI_{i,3} + \frac{0}{189} PCI_{i,4}\right) \times 100\%$$

where

$\overline{PCI_i}$ ≡ Weighted Preparatory Checks Index of ith city/municipality

$PCI_{i,s}$ ≡ Preparatory Checks Index of ith city/municipality for s^{th} hydro-meteorological hazard

$C_{i,s}$ ≡ Indicator variable if the ith city/municipality conducted preparatory checks after hearing / receiving the warning for s^{th} hydro-meteorological hazard (1 – Yes, 0 – No)

$PC_{ij,s}$ ≡ Indicator variable of the jth preparatory check conducted by i^{th} city/municipality for s^{th} hydro-meteorological hazard (1 – Yes, 0 – No)

i ≡ city/municipality ≡ 1, 2, 3, ..., 189

j ≡ type of preparatory checks ≡ {Check inventory of supplies and equipment, Check capacity of critical facilities like hospitals, Organize DRRM teams and personnel, Enlist volunteers, Prepare evacuation centers, Prepare and preposition relief goods}

s ≡ type of hydro-meteorological hazard ≡ {1- Strong winds & rain, 2 - Flood, 3 - Landslide, 4 – Big waves}

19.3. Warning Issued Index (WII)

The WII is the geometric average of two components: 1. Product of presence of warning to the constituents and when it was issued; and 2. Product of presence of warning to the constituents and via what medium (Television, Radio, SMS / Calls and Social media (e.g. Facebook, Twitter, etc.)

First, we calculate the index for each hydro-meteorological hazard (strong winds & rain, flood, landslide and big waves) using arithmetic mean,

$$WII_{i,s} = \sqrt{\frac{I_{i,s} \times W_{i,s}}{5} \times \frac{\sum_{j=1}^{4} I_{i,s} \times M_{ij,s}}{4}} \times 100\%$$

then the weighted mean of the four hydro-meteorological hazards are calculated,

$$\overline{WII_i} = \left(\frac{167}{189} WII_{i,1} + \frac{20}{189} WII_{i,2} + \frac{2}{189} WII_{i,3} + \frac{0}{189} WII_{i,4}\right) \times 100\%$$

where

$\overline{WII_i}$≡ Weighted Warning Issued Index of ith city/municipality

$WII_{i,s}$ ≡ Warning Issued Index of ith city/municipality for s^{th} hydro-meteorological hazard

$I_{i,s}$ ≡ Indicator variable if the ith city/municipality issue a warning to constituents for s^{th} hydro-meteorological hazard (1 – Yes, 0 – No)

Ravago, Majah-Leah; Mapa, Claire Dennis; Sunglao, Jun Carlo; Aycardo, Angelie Grace (2020), "Data from a Survey of the Philippines' Local Governments on their Risk Management Strategies to Natural Disasters", Mendeley Data, V1, doi: 10.17632/9y2yhw45zt.1

$W_{i,s}$ ≡ Ordinal variable of when warning to constituents was issued ≡ {More than 24 hours before disaster,
24 hours or less before disaster, During disaster, Less than 24 hours after disaster, More than 24
hours after disaster}
$M_{ij,s}$ ≡ Indicator variable of the jth medium used for warning by i^{th} city/municipality for s^{th} hydro-meteorological hazard (1 – Yes, 0 – No)
j ≡ medium for warning ≡ {PAGASA/DOST, Provincial DRRMO, NDRRMC, Local media}
i ≡ city/municipality ≡ 1, 2, 3, ..., 189
s ≡ type of hydro-meteorological hazard ≡ {1- Strong winds & rain, 2 - Flood, 3 – Landslide,4 – Big waves}
